# Development of mechanically optimized biomimetic hybrid scaffold–stem cell constructs for functional tendon regeneration and restoration of mechanical integrity

**DOI:** 10.1371/journal.pone.0359084

**Published:** 2026-09-28

**Authors:** Alani Mohanad Khalid Ahmed

**Affiliations:** Department of Microbiology, College of Medicine, Tikrit University, Salahaldin, Iraq; UCAM: Universidad Catolica San Antonio de Murcia, CHINA

## Abstract

Tendon ruptures heal poorly due to hypocellularity, disorganized extracellular matrix (ECM), and inadequate mechanical loading. Current scaffold-stem cell strategies lack integration of dynamic mechanical cues and sustained growth factor delivery. The objective of this study is to engineer a mechanically tuned, bioactive hybrid scaffold system that integrates tendon-derived stem cells (TDSCs) and mesenchymal stem cells (MSCs), controlled growth factor release, and progressive mechanical stimulation to achieve functional tendon regeneration with enhanced structural and mechanical recovery. Scaffolds were fabricated with controlled pore architecture and fiber alignment. TDSCs were seeded dynamically and cultured under cyclic uniaxial strain (5%, 1 Hz, 4 h/day) with TGF-β3-loaded PLGA microspheres (release >21 days). In vitro assays assessed viability, proliferation, scleraxis/tenomodulin expression, collagen I/III ratio, and GAG content. In vivo evaluation used a rat Achilles tendon defect model (6 mm gap) with four groups: scaffold-only, scaffold+TDSCs, scaffold+TDSCs + TGF-β3, and scaffold+TDSCs + TGF-β3 + mechanical preconditioning (the full composite). Outcomes at 6 and 12 weeks included biomechanical testing, histology (collagen alignment, vascularity), and immunohistochemistry. The full composite group achieved 92% cell viability at 14 days, 8-fold upregulation of scleraxis, and organized collagen I deposition (60 ± 6 µg/mg). Cyclic strain improved collagen alignment by 3.5-fold versus static controls. In vivo, the full composite restored 88% of native tendon tensile strength by week 6 and 95% by week 12, with minimal adhesion formation and near-native ECM organization. The preconditioned groups showed significantly lower inflammatory scores and higher tenomodulin expression. Mechano-activated, growth factor-eluting scaffolds with TDSCs achieve superior tendon regeneration by combining biochemical and biophysical cues. This strategy offers a scalable, off-the-shelf, or autologous solution for clinical tendon repair.

## Introduction

Tendon injuries represent a significant global healthcare burden, affecting millions yearly and presenting significant challenges in orthopedic and sports [1]. Tendons have fundamentally poor healing ability because of their hypocellular characteristics, limited vascular supply, and dense, organized extracellular matrix (ECM) architecture. Even after surgery, repaired tendons often recover with disordered scar tissue that impairs mechanical performance, resulting in chronic pain, long-term impairment, and significant re-injury rates, up to 50% in some clinical groups [2,3].

The initial surgical repair, autografting, allografting, and rehabilitation regimens used in current clinical care procedures are insufficient to restore native tendon function. For instance, the anisotropic collagen organization necessary for adequate load transfer cannot be recapitulated by surgical repair alone, although it does give instant mechanical continuity [4]. Allografts run the risk of immunological rejection and disease transmission, whereas autograft harvesting is constrained by donor site morbidity and tissue availability [5]. These drawbacks highlight the critical need for regenerative techniques that address both mechanical restoration and biological healing at the same time.

Achieving the right balance between mechanical strength and biological integration is a basic challenge in tendon tissue engineering [6,7]. Only one facet of this dualism is usually addressed by conventional methods. For example, synthetic scaffolds can offer strong mechanical support, but they frequently lack the bioactive signals required for matrix remodeling, tenogenic differentiation, and cell attraction [8]. However, growth factor or stem cell therapies may improve biological healing, they do not offer the structural guidance necessary for structured ECM deposition, which leads to repair tissue that is mechanically incompetent [9].

Recent developments in regenerative medicine have presented potential approaches that combine stem cell populations with biocompatible scaffolds [10,11]. Mesenchymal stem cells (MSCs) and tendon-derived stem cells (TDSCs) have the capacity for multilineage differentiation and have been demonstrated to facilitate tendon regeneration via both direct tenogenic differentiation and paracrine signaling [9,12,13]. However, stem cell-based techniques are insufficient on their own without the appropriate biophysical and biochemical microenvironmental cues that direct lineage selection and matrix organization [14].

One important but commonly disregarded aspect of tendon regeneration is the incorporation of mechanical stimulation. Tenocyte phenotypic maintenance and ECM homeostasis depend on mechanotransduction pathways, which include transcription factors like scleraxis and signaling molecules like TGF-β. Native tendons grow and function under dynamic mechanical loads [15,16]. It has been shown that cyclic mechanical strain improves the mechanical characteristics of synthetic tendon structures, aligns collagen fibers, and upregulates tenogenic markers [17,18]. However, most existing scaffold-stem cell approaches do not include mechanical preconditioning before implantation, which could limit functional results.

Another option for improving tendon regeneration is controlled growth factor administration. A key player in tenogenic differentiation, transforming growth factor-beta 3 (TGF-β3) suppresses non-tenogenic lineages while promoting the expression of scleraxis, tenomodulin, and collagen type I [19–21]. However, growth factor bolus administration leads to quick elimination and inadequate bioactivity. Biodegradable polymers, such poly(lactic-co-glycolic acid) (PLGA) microspheres, are used in sustained-release systems to sustain therapeutic growth factor levels across clinically relevant periods (≥21 days) [22–24]. These systems provide persistent biochemical cues that work in concert with mechanical stimulation.

Despite these developments, no current approach has effectively combined all essential elements, such as stem cell populations, biomimetic scaffold architecture, persistent growth factor supply, and mechanical preconditioning, into a cohesive system for functional tendon regeneration. There is a substantial knowledge gap regarding the individual and combined contributions of these elements to tendon healing outcomes.

The objective of this study was to engineer a mechanically tuned, bioactive hybrid scaffold system integrating TDSCs, sustained TGF-β3 release, and progressive mechanical preconditioning to achieve functional tendon regeneration with improved structural and mechanical recovery. The study was designed as a sequential translational investigation comprising scaffold fabrication, in vitro biological validation, and preclinical in vivo assessment. The study hypothesized that: (1) mechanical preconditioning would enhance tenogenic differentiation and ECM organization in vitro, (2) sustained TGF-β3 delivery would promote tendon-specific matrix production, and (3) the full composite construct would restore native tendon mechanical properties and histological architecture in vivo. This study created electrospun collagen and 3D-printed PLA scaffolds with controlled pore architecture in order to test these hypotheses. The study then seeded the scaffolds with TDSCs under dynamic conditions, subjected the constructs to cyclic uniaxial strain using TGF-β3-loaded PLGA microspheres, and assessed the results using a rat Achilles tendon defect model.

This study is clinically significant and contribute to orthopaedic research because it addresses the fundamental failure of current tendon repair strategies, such as the inability to simultaneously restore mechanical integrity and biological function. This study achieves 95% restoration of native tensile strength and physiologic failure strain by 12 weeks by integrating four synergistic components (biomimetic scaffolds, TDSCs, sustained TGF-β3 delivery, and mechanical preconditioning) into a single construct, in contrast to previous approaches that combine only two elements. In tendon tissue engineering, this degree of functional recovery is uncommon. The six-group factorial design offers mechanistic insight by separating the individual contributions of each component. It shows that mechanical preconditioning improves collagen alignment by 3.5 times, while persistent growth factor supply results in 8-fold upregulation of scleraxis. Clinically, this method provides autologous choices for elective treatments as well as availability for acute injuries, with direct applications in segmental defect reconstruction, Achilles tendon rupture, rotator cuff tears, and revision surgery. This scaffold-stem cell approach has the potential to improve outcomes for millions of patients annually while lowering healthcare costs associated with revision surgeries and prolonged disability by reducing re-rupture rates (currently 5–10% with conventional repair). This also remove donor site morbidity linked to autografts and preventing disease transmission risks of allografts.

## Materials and methods

### Experimental design

A six-group factorial design was employed to isolate individual and synergistic contributions of scaffold architecture, stem cells, sustained growth factor delivery, and mechanical preconditioning to tendon regeneration. A total of 72 adult male Sprague Dawley rats (8–10 weeks old, 280–320 g) were randomly assigned into six groups (n = 12 per group, 6 per time point).

Group 1 (scaffold only, acellular) received electrospun collagen/3D-printed PLA scaffold without stem cells or growth factors. Group 2 (scaffold + TDSCs) received scaffold seeded with TDSCs (2 × 10^5^ cells/scaffold) cultured under static conditions. TDSCs were isolated from Achilles tendons of healthy Sprague–Dawley rats (8–10 weeks old) using an established enzymatic digestion protocol. Group 3 (scaffold + TDSCs + TGF-β3) received scaffold seeded with TDSCs and embedded with TGF-β3-loaded PLGA microspheres providing sustained release over 21 days. Group 4 (full composite) received scaffold seeded with TDSCs, embedded with TGF-β3 microspheres (PeproTech, USA), and subjected to cyclic mechanical preconditioning (5% strain, 1 Hz, 4 hours/day for 7 days) prior to implantation. The rationale for chosen these mechanical preconditioning or loading parameters was to reproduce the physiological mechanical environment experienced by native tendons during normal locomotion. Previous studies have indicated that cyclic strains within the physiological range (4–6%) efficiently promote tenogenic differentiation, extracellular matrix deposition, collagen synthesis, and cellular alignment [6,7,17]. In addition to preventing excessive deformation that can induce apoptosis and matrix damage [17]. Tendon mechanotransduction has been widely stimulated in vitro with a loading frequency of 1 Hz, which closely resembles the cadence of typical walking. In order to minimize cellular fatigue and overloading and induce extracellular matrix remodeling and tendon-specific gene expression, daily mechanical stimulation for 4 hours/day for 7 days offers enough cumulative loading [18]. Hence, these loading parameters were chosen based on previously validated tendon tissue engineering protocols to optimize scaffold maturation before implantation. Group 5 (sham control) underwent surgical tendon defect creation with primary suture repair without scaffold implantation. Group 6 (healthy control) consisted of uninjured, unoperated rats with intact Achilles tendons to define native mechanical and histological baselines. All animal procedures were approved by the Institutional Animal Care and Use Committee of Tikrit University (Approval No. TU-IACUC-2025–103).

### Experimental procedures

#### Scaffold fabrication with controlled pore architecture, biocompatible, dynamic preconditioning, and in vivo evaluation.

Biocompatible scaffolds with regulated pore architecture (TenIGA) were meticulously created to replicate the structural and functional properties of the original extracellular matrix (ECM) in order to provide a functioning tendon substitute. Fig 1 displays schematic illustration of scaffold fabrication, biofunctionalization, mechanical preconditioning, and in vivo evaluation workflow. Natural polymers such as type I collagen (Sigma-Aldrich, St. Louis, MO, USA) and silk fibroin were selected for their bioactivity and cell adhesion properties [25]. Synthetic polymers like polycaprolactone (PCL; Sigma-Aldrich, USA) and polylactic acid (PLA; NatureWorks LLC, Japan) provided tunable mechanical integrity and durability [26]. Collagen was dissolved in 0.1 M acetic acid (10 mg/mL) under constant stirring for 24 hours at room temperature [27]. Silk fibroin was extracted from *Bombyx mori* cocoons by de-sericination in boiling sodium carbonate solution, dissolved in lithium bromide at 60°C, purified via dialysis, and adjusted to 8% (w/v). PCL was dissolved in a 7:3 mixture of dichloromethane and dimethylformamide (10% w/v) with stirring for 4 hours, while PLA was dissolved in chloroform (10% w/v) with gentle stirring at 40°C for 3 hours. Electrospinning produced fibrous scaffolds with aligned fibers that resembled the anisotropic structure of tendons using sophisticated fabrication techniques, while 3D printing allowed for exact customization of pore size and geometry to improve ECM deposition and cell infiltration [28,29]. Natural polymer-based scaffolds were crosslinked in 0.25% glutaraldehyde in PBS for 2 hours, and all scaffolds were sterilized by immersion in 70% ethanol for 30 minutes followed by UV exposure for 1 hour to ensure safety for cell seeding.

**Fig 1:**
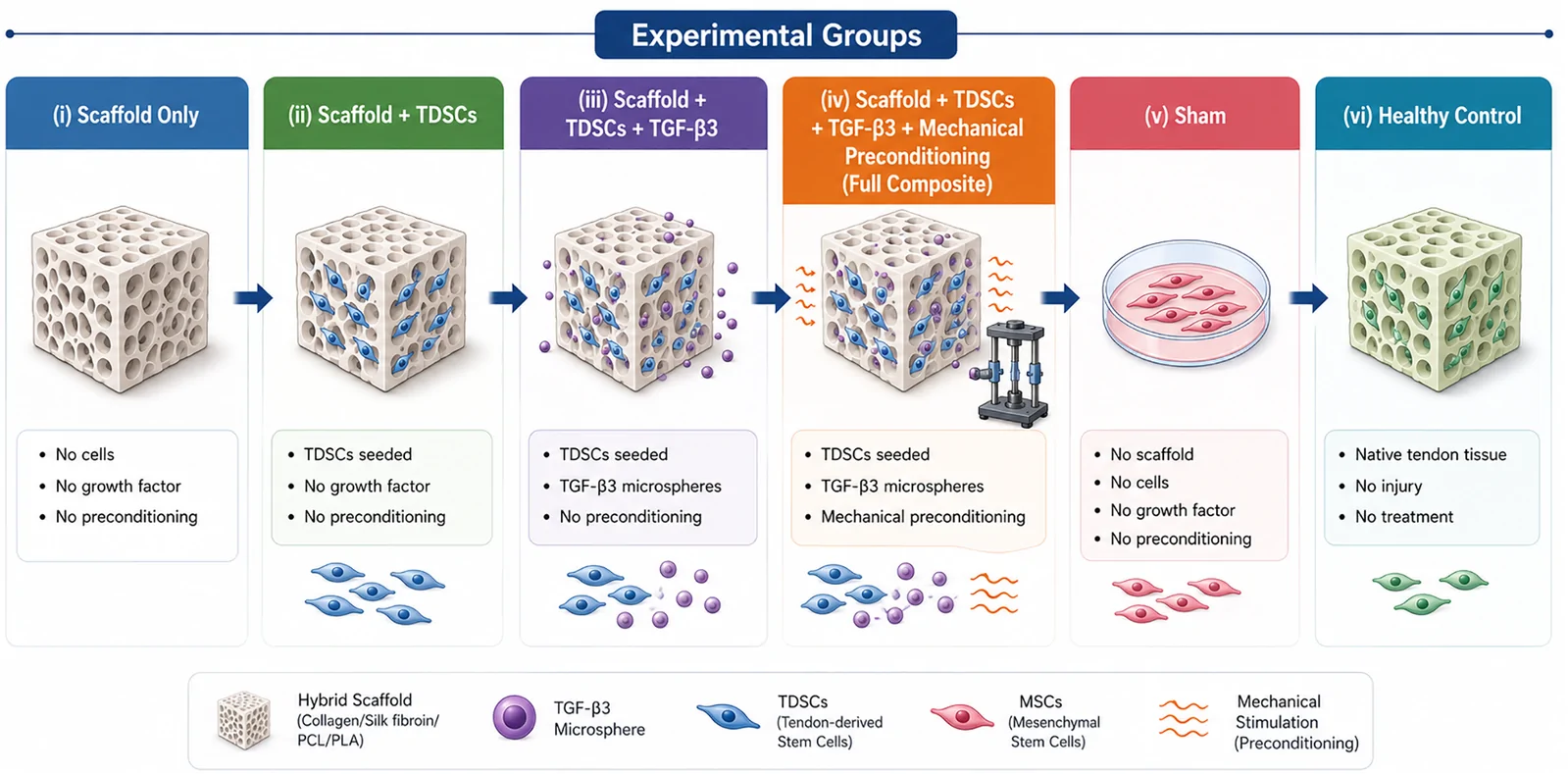
Schematic illustration of scaffold fabrication, biofunctionalization, mechanical preconditioning, and in vivo evaluation workflow. Biocompatible hybrid scaffolds were fabricated using natural (collagen, silk fibroin) and synthetic polymers (polycaprolactone, polylactic acid) to achieve controlled pore architecture and fiber alignment mimicking native tendon extracellular matrix (ECM). Scaffolds were subsequently biofunctionalized with growth factor–loaded microspheres (e.g., TGF-β3) to enable sustained release. Tendon-derived stem cells (TDSCs) and/or mesenchymal stem cells (MSCs) were seeded onto the scaffolds using dynamic culture conditions to promote uniform cell distribution and attachment. Constructs were then subjected to mechanical preconditioning in a bioreactor system under cyclic uniaxial strain (5% strain, 1 Hz, 4 h/day) to enhance tenogenic differentiation, extracellular matrix deposition, and collagen fiber alignment. Experimental groups included: (i) scaffold only, (ii) scaffold + TDSCs, (iii) scaffold + TDSCs + TGF-β3, (iv) scaffold + TDSCs + TGF-β3 + mechanical preconditioning (full composite), (v) Sham, and (vi) Healthy control. Tissue regeneration was assessed at defined time points using histological staining (H&E, Masson’s Trichrome, Picrosirius Red), immunohistochemistry, and biomechanical testing to evaluate structural organization, collagen maturation, inflammatory response, and functional recovery. This figure was created by the author for this study and does not reproduce any copyrighted material.

The fabricated scaffolds were trimmed into rectangular constructs measuring 5 × 3 × 1.5 mm before implantation. Electrospun fibers had an average diameter of 650 ± 120 nm, while interconnected pores ranged from 50–100 μm with an overall scaffold porosity of approximately 85%.

The fabricated scaffolds supported a biosome ECM environment and were designed for growth factor elution to promote tenogenic differentiation (Fig 1). TDSCs and MSCs were seeded onto the scaffolds. MSCs were isolated from the femur and tibia of donor rats by density-gradient centrifugation. Cells were cultured under standard conditions and only passages 3–5 were used for all experiments to ensure phenotypic stability and minimize senescence. Transforming growth factor beta-3 (TGF-β3) was incorporated either directly or via sustained-release microspheres. Four experimental groups were prepared: scaffold only, scaffold with TDSCs, scaffold with TDSCs plus free TGF-β3, and scaffold with TDSCs plus TGF-β3 microspheres. This approach ensured that the scaffolds provided both biological compatibility and mechanical integrity while actively directing stem cell differentiation toward a tendon lineage.

Cell-seeded scaffolds were dynamically preconditioned utilizing cyclic strain to mimic the natural mechanical environment of tendons and promote tenogenic development (Fig 1). 5% strain was delivered at a frequency of 1 Hz for four hours per day as part of the regimen. Before the designed tendon construct was implanted in vivo, this mechanical stimulation encouraged cellular alignment, ECM organization, and functional maturation.

In vivo evaluation was performed using a rat Achilles tendon injury model with a 6 mm full-length defect. The four experimental groups (scaffold only, scaffold + TDSCs, scaffold + TDSCs + TGF-β3, and scaffold + TDSCs + TGF-β3 microspheres) were implanted into the defect sites. Post-implantation outcomes were assessed using H&E staining to evaluate tissue morphology and cellular infiltration. Histological analysis also monitored for ectopic tissue formation, including metastasis and osteosarcoma. Finally, biomechanical testing was conducted to measure tensile strength, stiffness, and failure load, providing a quantitative assessment of functional tendon repair.

### Stem cell integration

The process of stem cell integration entailed separating and seeding cells onto the scaffolds in environment that supported their survival, growth, and differentiation [30]. Two stem cell populations were employed in this study. MSCs and TDSCs were incorporated into the scaffold because they provide complementary biological functions during tendon regeneration. MSCs were selected for their robust proliferative capacity, immunomodulatory properties, paracrine secretion of regenerative growth factors, and ability to support early extracellular matrix remodeling. TDSCs were selected because they are tendon-resident progenitor cells with superior tenogenic differentiation capacity and enhanced expression of tendon-specific markers, including scleraxis (SCX), tenomodulin (TNMD), and collagen type I. The combined use of both cell populations was therefore intended to promote both the early regenerative response and the subsequent formation of tendon-specific tissue within the bioactive scaffold.

Rat TDSCs was isolated from the Achilles tendons of healthy male Sprague–Dawley rats (8–10 weeks old, 250–300 g), were incorporated because of their superior tenogenic differentiation potential and tendon-specific extracellular matrix production. Following aseptic excision, tendon tissues were minced into approximately 1 mm^3^ fragments and enzymatically digested with collagenase type I (Worthington Biochemical Corporation, USA). Both MSCs and TDSCs were cultured under identical conditions. After enzymatic digestion, the isolated cells were cultured in Dulbecco’s Modified Eagle Medium (DMEM; Gibco, Thermo Fisher Scientific, USA) supplemented with 10% fetal bovine serum (FBS; Gibco, Thermo Fisher Scientific, USA) and 1% penicillin–streptomycin (Gibco, Thermo Fisher Scientific, USA). Cells were maintained at 37°C in a humidified incubator with 5% CO_2_, with the culture medium replaced every 2–3 days. Cells at passages 3–5 were used for subsequent scaffold seeding and experimental analyses.

To evaluate the regeneration capacity of MSCs, bone marrow taken from the tibia or femur was used to separate the cells, which were then grown under controlled conditions [31]. After collected into heparinized syringes, the bone marrow was diluted with PBS and placed on top of Ficoll-Paque in order to isolate mononuclear cells by centrifugation. The separated cells were cleaned, reconstituted in low-glucose DMEM supplemented with 1% penicillin-streptomycin and 10% FBS, and then planted at a density of 2 × 10^5^ cells/cm^2^ in T75 culture flasks. Cultures were kept at 37°C with 5% CO_2_, and after 48 hours, non-adherent cells were extracted. The media was changed every three days. The MSCs were prepared for scaffold seeding by passing at 80% confluency, which was typically accomplished in 5 days.

### Cell characterization

Flow cytometric characterization was performed to confirm the phenotypic identity of both TDSCs and MSCs before scaffold seeding. TDSCs displayed positive expression of CD90, CD44, and CD105, with minimal expression of the hematopoietic markers CD34 and CD45. MSCs satisfied the International Society for Cell and Gene Therapy (ISCT) minimal criteria for mesenchymal stem cells. Cells were cultured in DMEM (Gibco, Thermo Fisher Scientific, USA) supplemented with 10% fetal bovine serum (FBS; Gibco, Thermo Fisher Scientific, USA) and 1% penicillin–streptomycin (PAN-Biotech, Germany) at 37°C in a humidified atmosphere containing 5% CO_2_. Cells at passages 3–5 with viability greater than 95%, as determined by trypan blue exclusion, were used for scaffold seeding.

### Cell Seeding and Culture

Five scaffolds were prepared for each group (Groups 1–4) in accordance with the factorial design to guarantee uniformity and reproducibility under all experimental settings. Only Groups 2–4 involved cell seeding, while Group 1 (acellular scaffold) served as a material control, and Groups 5 (sham) and 6 (healthy control) did not undergo scaffold seeding. All scaffolds were thoroughly rinsed with phosphate-buffered saline (PBS), sterilized with 70% ethanol, and preconditioned in low-glucose Dulbecco’s Modified Eagle Medium (DMEM; Gibco, Thermo Fisher Scientific, USA) supplemented with 10% fetal bovine serum (FBS; Gibco, Thermo Fisher Scientific, USA) to improve protein adsorption and encourage cell adhesion before cell seeding [32].

TDSCs and MSCs were harvested at 80% confluency and resuspended in complete culture medium at a density of 1 × 10⁶ cells/mL. For Groups 2–4, each scaffold was seeded with 50 µL of cell suspension (equivalent to 2 × 10^5^ cells per scaffold). A dynamic seeding method [33], which involves gently rotating scaffolds for an hour at 37°C in a humidified atmosphere with 5% CO_2_, was used to improve seeding efficiency and produce uniform cell distribution.

Constructs were moved into complete culture medium after initial attachment, and they were kept under conventional culture conditions with media changes every three days. Seeded constructs were cultivated in a rotating bioreactor system (30 rpm; custom uniaxial mechanical loading bioreactor, MicroTester, Italy), which promoted dynamic fluid flow and enhanced nutrient exchange, in order to further increase cell penetration and homogenous distribution inside the scaffold design [8].

Group-specific culture conditions were used as follows: Group 2 (scaffold + TDSCs) maintained under static culture conditions following seeding. Group 3 (scaffold + TDSCs + TGF-β3) cultured in the presence of TGF-β3, delivered via embedded PLGA microspheres for sustained release. Group 4 (full composite) subjected to cyclic mechanical preconditioning (5% strain, 1 Hz, 4 hours/day) for 7 days using a bioreactor system to simulate physiological tendon loading and promote tenogenic differentiation. Cellular responses, including adhesion, proliferation, differentiation, and ECM deposition, were assessed at Days 3, 7, and 14 using quantitative and imaging-based assay.

### Biofunctionalization

Biofunctionalization was carried out by including transforming growth factor-beta 3 (TGF-β3) via a controlled release mechanism to increase the tenogenic potential of scaffold constructions [34]. Group 3 (scaffold + TDSCs + TGF-β3) and Group 4 (full composite) were the only groups to receive this strategy; Groups 1, 2, 5, and 6 were used as non-growth factor controls. Poly(lactic-co-glycolic acid) (PLGA; Evonik Industries, Germany) microspheres were used to encapsulate TGF-β3 for long-term, targeted administration [35]. In brief, TGF-β3 (10 µg/mL) was stabilized in 2% (w/v) polyvinyl alcohol (PVA; Sigma-Aldrich, USA) after being emulsified in a 5% (w/v) PLGA solution made in dichloromethane. Centrifugation was used to gather the microspheres, which were then lyophilized and cleaned to get rid of any remaining solvent. Following production, the resultant microspheres were applied by surface coating or uniformly integrated into the scaffold matrix at a concentration of 10 mg/mL. Functionalized scaffolds were incubated in PBS at 37°C for up to 21 days, and supernatants were collected at predetermined intervals to assess release kinetics. This strategy enabled a prolonged release profile by sustaining bioactive levels of TGF-β3 to promote stem cell proliferation, tenogenic differentiation, and ECM synthesis.

### Mechanical stimulation

Only Group 4 (complete composite: scaffold + TDSCs + TGF-β3 + mechanical stimulation) received mechanical preconditioning in order to mimic the physiological mechanical environment of native tendon tissue and improve functional development. A specially built bioreactor system was used to subject constructions to cyclic uniaxial strain after initial cell seeding and stabilization [36]. For seven days in a row, mechanical loading was administered at a frequency of 1 Hz and a strain of 5% for four hours each day, simulating physiologically appropriate tendon loading conditions. Tendon-specific markers such scleraxis and tenomodulin were elevated, collagen fiber organization was improved, and cellular alignment along the major strain axis was encouraged by this dynamic stimulation. However, Groups 2 and 3 were kept in static culture conditions in order to separate the impact of biological and biochemical cues from mechanical influence. All constructs were cultured in DMEM supplemented with 10% FBS and 1% penicillin-streptomycin, with media changes every three days. On Days 7 and 14, the effects of mechanical stimulation were evaluated using structural imaging and immunostaining analysis.

### Quantitative analysis of tendon-specific gene expression

After 14 days of culture, total RNA was extracted from the scaffold constructs using TRIzol™ Reagent (Invitrogen, Thermo Fisher Scientific, Waltham, MA, USA) according to the manufacturer’s instructions. RNA concentration and purity were determined using a NanoDrop™ spectrophotometer (Thermo Fisher Scientific), and only samples with an A260/A280 ratio between 1.8 and 2.0 were used for further analysis. Complementary DNA (cDNA) was synthesized using a High-Capacity cDNA Reverse Transcription Kit (Applied Biosystems, Foster City, CA, USA). Quantitative real-time PCR (RT-qPCR) was performed using PowerUp™ SYBR™ Green Master Mix (Applied Biosystems) on a QuantStudio™ 5 Real-Time PCR System (Applied Biosystems). The expression levels of SCX and TNMD were normalized to GAPDH, and relative gene expression was calculated using the 2^-ΔΔCt method.

Three primer pairs were used for RT-q PCR analysis in order to measure tendon-related gene expression and normalize against a housekeeping control. The forward sequence CGACCGCGAGATGAACACCT and the reverse sequence CGTGTTCCGCAGCCATCTTG, which together amplified a 98-base-pair product, were primer set used for SCX. An amplicon of 112 base pairs was produced for TNMD using the forward primer GCTGGCACAGCTACCAAAGA and the reverse primer CCAGGCACAGGGTGAAGAAT. Lastly, an 85-base-pair product was obtained by amplifying the internal reference gene GAPDH using the forward primer GGAGCGAGATCCCTCCAAAAT and the reverse primer GGCTGTTGTCATACTTCTCATGG. All primers were made at melting temperatures of about 60°C and had their specificity and efficiency confirmed before utilized in experimental runs.

### In vitro assessment

In vitro evaluations were performed to assess scaffold biocompatibility, cellular activity, ECM production, and mechanical performance of the experimental groups. Since Group 5 (sham control) and Group 6 (healthy control) are in vivo reference groups and were excluded from scaffold-based in vitro tests, these analyses mainly concentrated on Groups 1–4.

#### Cell viability and cytocompatibility.

The MTT test and Live/Dead fluorescence staining were used to measure cell viability and metabolic activity. Group 1 (scaffold-only) acted as an acellular control for baseline scaffold biocompatibility, while Groups 2–4 (cell-seeded constructions) were the subjects of these assessments. Scaffold constructs were incubated for 30 minutes at 37°C in a solution containing 4 µM ethidium homodimer-1 (dead cells) and 2 µM calcein-AM (living cells) for living/Dead staining. The distribution, vitality, and morphology of the cells were then assessed by fluorescence imaging using a confocal microscope [37]. Constructs were treated with 500 µL of 10% MTT solution at 37°C for four hours in order to facilitate the production of formazan crystals for the MTT test. After dissolving the crystals in 500 µL of dimethyl sulfoxide (DMSO), a microplate reader was used to detect the absorbance at 570 nm. In order to evaluate cell growth and metabolic activity throughout time, measurements were made on Days 1, 7, and 14. Each group’s five to six scaffolds were examined at each time point.

#### ECM production and organization.

Collagen content, glycosaminoglycan (GAG) levels, and collagen type I expression were measured to quantify ECM synthesis in Groups 2–4, with Group 1 acting as a baseline scaffold control. A hydroxyproline assay was used to evaluate the total collagen content [38]. Scaffold constructs were hydrolyzed in 6 N HCl, then reacted with Ehrlich’s reagent and chloramine-T, and spectrophotometric measurement was performed at 560 nm. The dimethylmethylene blue (DMMB) test was used to assess GAG concentration. Papain-digested scaffold lysates were reacted with DMMB dye and detected at 525 nm. Collagen type I immunofluorescence staining was used for both qualitative and spatial evaluation [27]. The constructs were incubated with primary antibodies against collagen I (Abcam, ab34710) and then secondary antibodies conjugated with Alexa Fluor (Abcam, Cambridge, UK) after blocked with 3% bovine serum albumin (BSA) and preserved in 4% paraformaldehyde. Fluorescence microscopy imaging was used to assess the distribution and organization of ECM. To evaluate the temporal evolution of matrix deposition and maturation, all ECM-related studies were carried out at Days 7 and 14 utilizing the five scaffolds per group.

#### Mechanical characterization of scaffold constructs.

A universal testing apparatus (AGX-V2 Series, Shimadzu, Japan) with a 50 N load cell was used to assess the mechanical characteristics of scaffolds. Rectangular scaffold samples (10 mm × 5 mm) from Groups 1–4 was hydrated in PBS and then subjected to uniaxial tensile testing at a strain rate of 1 mm/min until failure [18]. Elongation at break, elastic modulus, and ultimate tensile strength were among the important mechanical parameters that were measured [6]. Cell-seeded constructs (Groups 2–4) and acellular scaffolds (Group 1) were compared in order to ascertain the impact of cellular activity and matrix deposition on scaffold dynamics. Five independent samples were used for each condition to ensure statistical robustness and reproducibility.

The study was conducted in two sequential experimental stages. The first stage focused on the fabrication, optimization, and preliminary biological evaluation of the scaffold, and the second stage assessed its therapeutic performance in a rat Achilles tendon defect model. Both stages constituted a single translational study.

### In vivo assessment

#### Experimental I.

Sprague Dawley rats were obtained at 6 weeks of age (140–150 g) from the Department of Animal Production, Directorate of Agricultural Research, Sulaymaniyah, Kurdistan Region, Iraq. Animals were acclimatized and maintained under standard laboratory conditions, including a controlled temperature of 22–26 °C, relative humidity of 50–70%, and 10–15 air exchanges per hour. Rats were housed on corncob bedding under a 16:8 h light–dark cycle with minimal exposure to high-frequency noise (<85 dB). Standard laboratory chow (protein: 14–20%, fat: 4–7%, fiber: 3–8%, supplemented with vitamins A, D, E, K, calcium, and phosphorus) and water were provided ad libitum.

A total of 36 adult Sprague Dawley rats (male ≈ 400 g; female ≈ 300 g) were used and randomly allocated into six experimental groups (n = 6 per group) according to the study design (Fig 2). All experimental procedures were reviewed and approved by the Institutional Animal Care and Use Committee (IACUC) of Tikrit University (Approval No. TU-IACUC-2025–088; dated 15 January 2026). All methods were conducted in accordance with the ARRIVE guidelines and the National Institutes of Health (NIH) Guide for the Care and Use of Laboratory Animals (8th edition).

**Fig 2:**
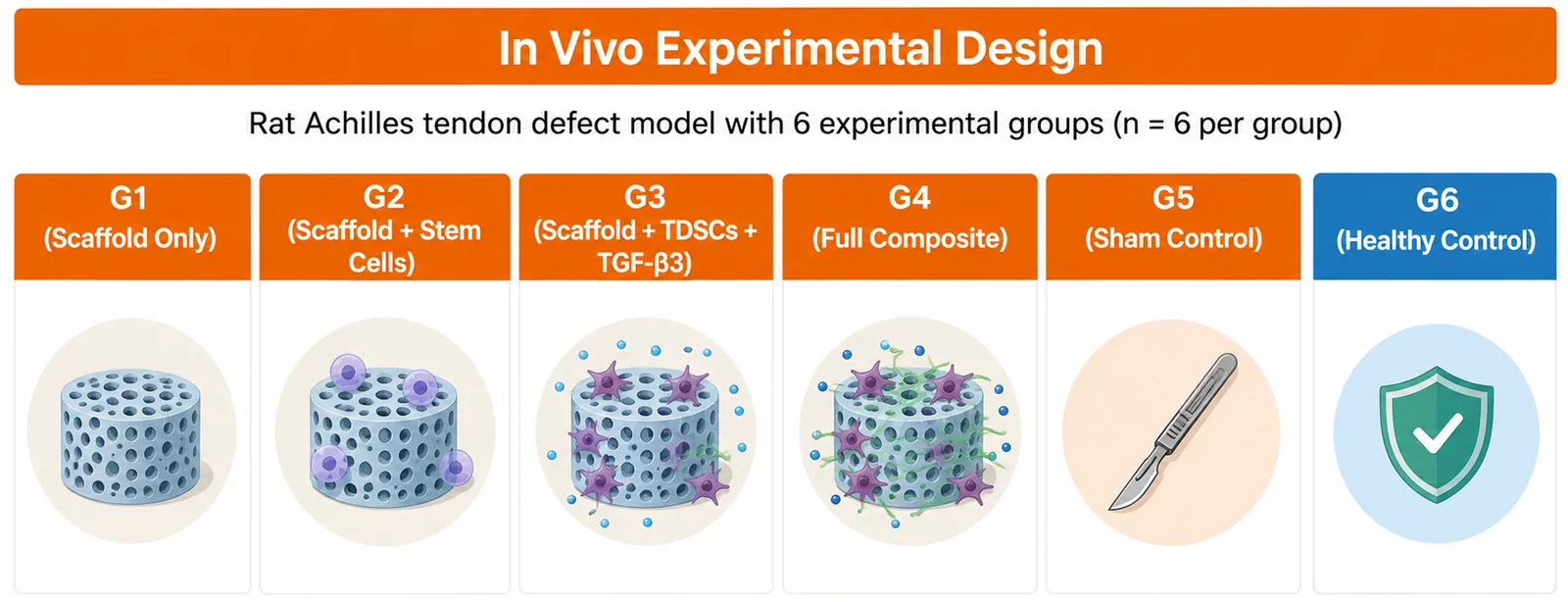
In vivo experimental design for Achilles tendon regeneration using a six-group rat model. The design for evaluating tendon regeneration in a rat Achilles tendon defect model. (A) G1 (Scaffold Only) are rats received acellular scaffolds implanted into the tendon defect, serving as a material control to evaluate the intrinsic biocompatibility and mechanical support of the scaffold without cellular influence. (B) G2 (Scaffold + Stem Cells) are rats received collagen-based scaffolds seeded with TDSCs implanted into surgically created Achilles tendon defects to assess the combined effect of biomaterial support and cellular contribution. (C) G3 (Scaffold + TDSCs + TGF-β3): Rats received cell-seeded scaffolds incorporating TGF-β3 for sustained growth factor delivery, enabling assessment of biochemical stimulation on tendon healing. (D) G4 (Full Composite): Rats received scaffolds seeded with TDSCs, embedded with TGF-β3, and subjected to mechanical preconditioning prior to implantation, representing the fully integrated regenerative strategy. (E) G5 (Sham Control) are rats underwent surgical creation of Achilles tendon defects followed by immediate suture repair without scaffold implantation, representing standard clinical intervention. (F) G6 (Healthy Control): Uninjured rats with intact Achilles tendons served as a physiological baseline for normal tendon structure and function. All procedures involved the creation of a full-thickness Achilles tendon defect followed by implantation or repair according to group allocation. Tendons were sutured using interrupted 6−0 prolene sutures. Animals were monitored postoperatively and evaluated for functional recovery and tissue regeneration at designated time points. The figure was manually assembled using BioRender and Microsoft PowerPoint for scientific illustration and figure assembly and does not reproduce any copyrighted material.

The in vivo model was established to evaluate tendon regeneration across six experimental groups (Fig 2): Group 1 (scaffold only), acellular collagen scaffold implanted into the tendon defect. Group 2 (scaffold + stem cells), scaffold seeded with TDSCs. Group 3 (scaffold + TDSCs + TGF-β3), cell-seeded scaffold incorporating TGF-β3. Group 4 (full composite), mechanically preconditioned scaffold seeded with TDSCs and embedded with TGF-β3. Group 5 (sham control), tendon defect created and immediately repaired without scaffold implantation. Group 6 (healthy control), uninjured rats with intact Achilles tendons.

The right hind limb was shaved and aseptically prepared after anesthesia was induced by intraperitoneal administration of ketamine (50 mg/kg) and xylazine (5 mg/kg). Over the Achilles tendon, a 1.5 cm longitudinal incision was created. A sterile surgical blade was used to generate a full-thickness, 5 mm mid-substance tendon defect while maintaining the paratenon. Two interrupted 6−0 prolene sutures were used at each tendon end to fix the prescribed scaffold construct (5 × 3 × 1.5 mm) in the defect site for Groups 1–4. The defect in Group 5 (sham control) was produced and promptly fixed with conventional suturing without the need for scaffold implantation. The animals in Group 6 did not have any surgery 4−0 nylon sutures were used to seal the skin incision.

Buprenorphine (0.05 mg/kg, subcutaneously every 12 hours for 3 days) was used for postoperative analgesia, and enrofloxacin (5 mg/kg, subcutaneously once daily for 5 days) was given to prevent infection. Normal weight-bearing and controlled physiological loads were feasible because there was no immobility. Functional recovery and tissue regeneration were assessed at predetermined time points (8 and 12 weeks post-surgery). Evaluation criteria comprised collagen fiber alignment, cellular infiltration and vascularization, mechanical strength testing, and histological examination of tissue structure.

#### Experimental II.

The justification for this second factorial design experiment was to overcomes the limitations of the first experiment approach by identifying the mechanism of improvement rather than simply confirming that scaffold and stem cells performance. This experiment makes it possible to clearly isolate the actual impact of stem cells on tendon regeneration and distinguish between the effects of the scaffold material alone and those of the additional cellular component. The study determines if the biological activity of the implanted cells or the structural support of the biomaterial is responsible for the reported improvements. Hence, this group provides a crucial baseline for interpreting the heightened effects seen in more complex experimental settings, like those that include mechanical preconditioning and TGF-β3. Therefore, this experiment includes six experimental groups (scaffold-only, scaffold+TDSCs, scaffold+TDSCs + TGF-β3, full composite, sham, and healthy control) enables isolation of the individual and synergistic contributions of scaffold architecture, stem cells, sustained growth factor delivery, and mechanical preconditioning to tendon regeneration.

A total of 72 adult male Sprague Dawley rats (8–10 weeks old, body weight 280–320 g) were used in this study. All animals were obtained from the Animal House Facility, College of Veterinary Medicine, Tikrit University, Iraq. Rats were housed under standard environmental conditions: temperature 22–24°C, relative humidity 55 ± 5%, 12:12 hour light-dark cycle (lights on at 7:00 AM), with corncob bedding changed twice weekly. Animals were provided with standard pellet diet (protein 18%, fat 5%, fiber 5%, calcium 1%, phosphorus 0.8%) and autoclaved tap water ad libitum. All animals were acclimatized for 7 days prior to any surgical procedure. The experimental protocol was reviewed and approved by the (Approval No. TU-IACUC-2025–103, dated 13 March 2026). All procedures complied with the ARRIVE guidelines and the NIH Guide for the Care and Use of Laboratory Animals (8th edition).

Rats were randomly assigned into six experimental groups (n = 12 per group) using a computer-generated randomization sequence: Group 1 (scaffold only, acellular), received electrospun collagen/3D-printed PLA scaffold without stem cells or growth factors. Group 2 (scaffold + TDSCs), received scaffold seeded with TDSCs (2 × 10⁵ cells/scaffold) cultured under static conditions. Group 3 (scaffold + TDSCs + TGF-β3), rceived scaffold seeded with TDSCs and embedded with TGF-β3-loaded PLGA microspheres (sustained release over 21 days). Group 4 (full composite), received scaffold seeded with TDSCs, embedded with TGF-β3 microspheres, and subjected to cyclic mechanical preconditioning (5% strain, 1 Hz, 4 hours/day for 7 days) prior to implantation. Group 5 (sham control, underwent surgical tendon defect creation and primary suture repair without any scaffold implantation. Group 6 (healthy control), uninjured, unoperated rats with intact Achilles tendons, used to define native mechanical and histological baselines (Fig 3).

**Fig 3:**
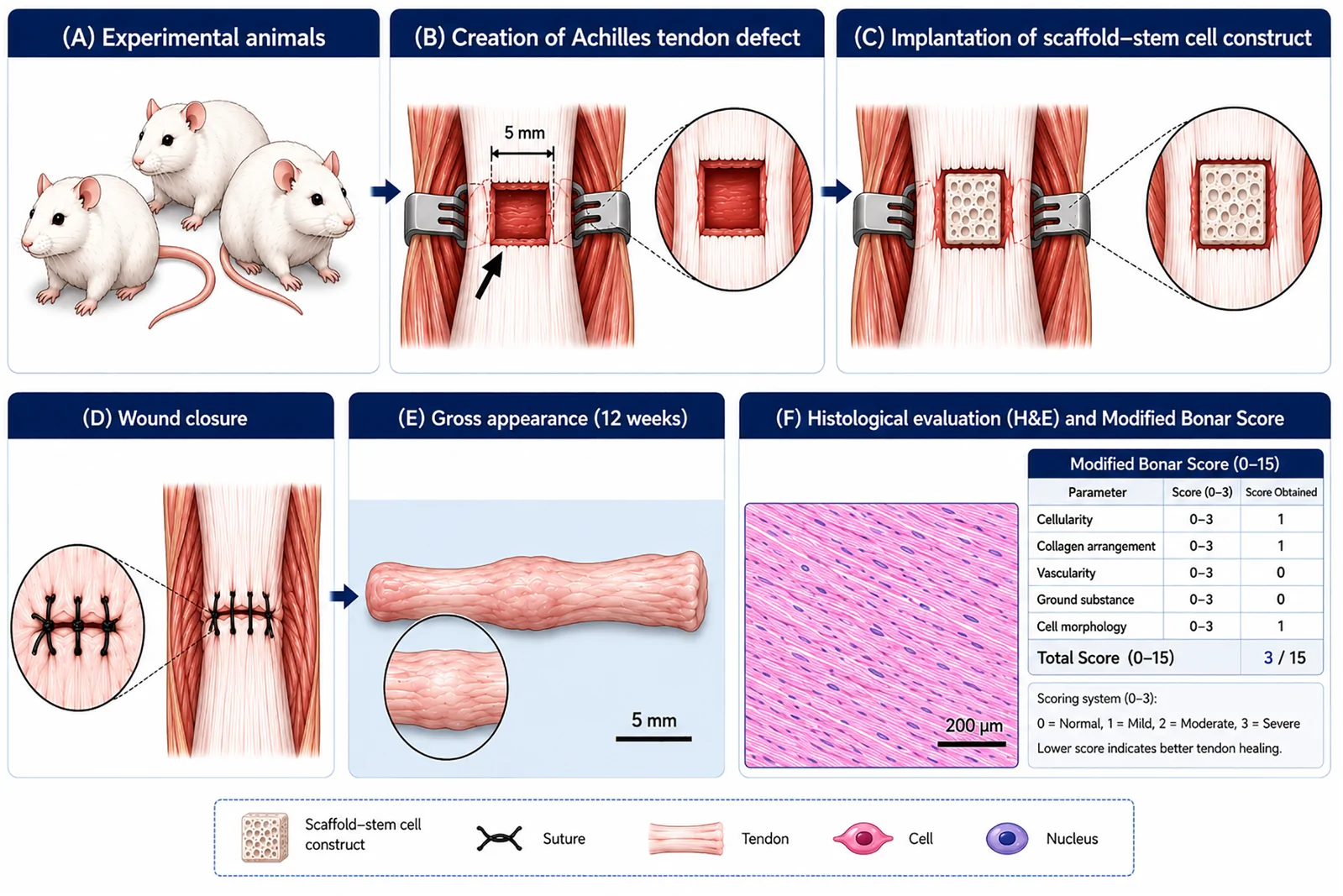
Experimental design and animal model. (A) Adult male Sprague Dawley rat (280–320 g) maintained under standard laboratory housing conditions prior to surgical intervention. (B) Surgical exposure of the Achilles tendon with creation of a standardized 5 mm full-thickness defect (indicated by arrow). (C) Implantation of the bioengineered scaffold–stem cell construct into the tendon defect site to promote regeneration. (D) Closure of the surgical incision using interrupted sutures following implantation. (E) Gross morphology of the explanted tendon specimen at 12 weeks post-surgery, demonstrating tissue continuity and repair (scale bar = 5 mm). (F) Representative hematoxylin and eosin (H&E)-stained histological section of regenerated tendon tissue, showing collagen fiber organization and cellular morphology (scale bar = 200 µm). All **Magnification scale**s = 200x unless otherwise indicated. **This figure was created by the authors for this study and does not reproduce any copyrighted material.**

All surgical procedures were performed under general anesthesia induced by intraperitoneal injection of ketamine (50 mg/kg) and xylazine (5 mg/kg) [39]. The right hind limb was shaved, disinfected with 10% povidone-iodine followed by 70% ethanol, and draped under sterile conditions. A 1.5 cm longitudinal incision was made over the Achilles tendon. A full-thickness, 5 mm mid-substance tendon defect was created using a sterile surgical blade, leaving the paratenon intact. The assigned scaffold construct (5 × 3 × 1.5 mm) was inserted into the defect site and secured using two interrupted 6−0 prolene sutures at each end. The skin was closed with 4−0 nylon sutures. In the sham group (Group 5), the defect was created and immediately closed with tendon sutures without scaffold placement. Postoperatively, buprenorphine (0.05 mg/kg subcutaneously every 12 hours for 3 days) was administered for analgesia, and enrofloxacin (5 mg/kg subcutaneously once daily for 5 days) was given to prevent infection. Rats were housed individually for the first 7 days post-surgery to prevent wound interference, then returned to group housing. No immobilization was used to allow natural weight-bearing and controlled mobilization.

Euthanasia and tissue harvesting were undertaken at two time points, 6 weeks (n = 6 per group) and 12 weeks (n = 6 per group) post-implantation [40,41]. Animals were thoroughly sedated with an overdose of pentobarbital sodium (150 mg/kg intraperitoneally). Following confirmation of loss of pedal response, the chest was opened, and transcardial perfusion was conducted with 100 mL of phosphate-buffered saline (PBS, pH 7.4) followed by 100 mL of 4% paraformaldehyde (PFA) in PBS for tissue fixation. The right Achilles tendon encompassing the repair site (extending 5 mm proximal and 5 mm distal to the defect) was meticulously dissected en bloc. Each harvested tendon was divided longitudinally into two halves: one half was placed in 10% neutral buffered formalin for 48 hours followed by paraffin embedding for histology and immunohistochemistry; the other half was wrapped in PBS-soaked gauze, snap-frozen in liquid nitrogen, and stored at −80°C for biochemical assays (collagen content, GAG quantification, and gene expression analysis). For biomechanical testing, a separate set of rats (n = 6 per group at each time point) underwent euthanasia by carbon dioxide inhalation followed by cervical dislocation, and the entire tendon–calcaneus–muscle unit was harvested, wrapped in saline-soaked gauze, and stored at −20°C until testing.

Following euthanasia, each rat was placed in a supine position, and the surgical site was re-opened. The skin and subcutaneous tissues were carefully reflected to expose the underlying Achilles tendon. The tendon was then transected 8 mm proximal to the defect site (near the myotendinous junction) and 8 mm distally at the calcaneal insertion, and the specimen was gently lifted using fine forceps without crushing. For histological analysis, specimens were pinned flat on a wax block to maintain tissue orientation and immediately fixed in formalin. For biochemical analysis, specimens were weighed, snap-frozen in liquid nitrogen, and subsequently pulverized under liquid nitrogen prior to homogenization. For biomechanical testing, the calcaneus was left attached to the specimen, which was kept hydrated in phosphate-buffered saline (PBS) at 4°C and tested within 4 hours of harvest.

Gross image of the harvested tendons was performed using a high-resolution digital camera (Canon EOS 90D with 100 mm macro lens) under standardized lighting. Representative images were taken of the surgical site immediately post-euthanasia (in situ), the explanted tendon (ex vivo, wet), and cross-sections of the repair site after sectioning.

Histological processing included fixation in 10% formalin for 48 hours, decalcification in 10% EDTA when the calcaneus was attached, dehydration in graded ethanol (70% to 95% to 100%), clearing in xylene, and embedding in paraffin wax. Sections of 5 µm thickness were cut using a rotary microtome (Leica RM2255) and stained with H&E for assessment of general morphology and inflammation, Masson’s Trichrome for evaluation of collagen distribution, and Picrosirius Red viewed under polarized light for analysis of collagen fiber alignment and maturity [42]. Histological sections were evaluated using the modified Bonar scoring system in order to offer an objective assessment of tendon regeneration. Five parameters, including collagen fibre organisation, cellularity, cell morphology, vascularity, and ground substance, were independently scored on a scale of 0 (normal) to 3 (marked abnormality), giving a total score ranging from 0 to 15, with lower scores indicating superior tendon healing.

Immunohistochemistry was performed on paraffin sections using primary antibodies against: tenomodulin (1:200; Abcam, ab203676), collagen type I (1:500; Abcam, ab34710), collagen type III (1:300, Abcam), and CD68 (1:250, for macrophage infiltration) [42]. Visualization was achieved using HRP-conjugated secondary antibodies and DAB chromogen. Immunohistochemical staining for Collagen type I, SCX and TNMD, was evaluated using the histological score (H-score) method. Staining intensity was classified as weak (1), moderate (2), or strong (3), and the percentage of positively stained cells at each intensity was recorded. The H-score was calculated using the following equation:

H-score = (1 × % weakly stained cells) + (2 × % moderately stained cells) + (3 × % strongly stained cells). Producing a score ranging from 0 to 300, where higher scores indicate greater protein expression. Additional immunohistochemical staining was performed using anti-CD80 and anti-CD163 antibodies to evaluate macrophage polarization within the regenerated tendon tissue. CD80-positive cells were considered representative of classically activated (M1) macrophages, while CD163-positive cells represented alternatively activated (M2) macrophages. Staining was quantified using the H-score method.

Biomechanical testing was conducted using a universal testing machine (Instron 5965, 50 N load cell) [42]. The tendon specimen was mounted with custom-designed pneumatic grips, pre-loaded to 0.1 N, preconditioned for 10 cycles (0–2% strain at 0.5 Hz), then loaded to failure at a rate of 1 mm/min. Parameters recorded included: ultimate tensile strength (MPa), Young’s modulus (MPa), and failure strain (%).

Blinding of all histological scoring, biomechanical analysis, and biochemical assays were performed by investigators blinded to group allocation. Sections were coded with random numbers, and the code was broken only after data collection was complete.

### Statistical methods

Statistical analysis was performed using GraphPad Prism 9.0 (GraphPad Software, San Diego, CA, USA). Data are presented as mean ± standard deviation (SD). Comparisons among multiple groups were performed using one-way analysis of variance (ANOVA) followed by Tukey’s post hoc test. A p-value < 0.05 was considered statistically significant.

## Results

### Scaffold mechanical properties

The mechanical properties of fabricated scaffolds were characterized under both static and preconditioned conditions (Fig 4A). Electrospun collagen scaffolds showed a tensile strength of 80 ± 10 MPa with elastic modulus of 250 ± 20 MPa, indicating moderate resistance to tension and deformation under physiological loads. Following mechanical preconditioning (5% cyclic strain, 1 Hz, 4 hours/day for 7 days), the tensile strength of collagen scaffolds increased to 85 MPa, while the elastic modulus showed a slight reduction to 245 MPa. This indicating improved ductility and strain accommodation without compromising structural integrity. The pore size of collagen scaffolds (50–100 μm) accelerates nutrient transport and cell infiltration, while the aligned fiber architecture promotes tenocyte alignment and mimics native tendon extracellular matrix organization.

**Fig 4:**
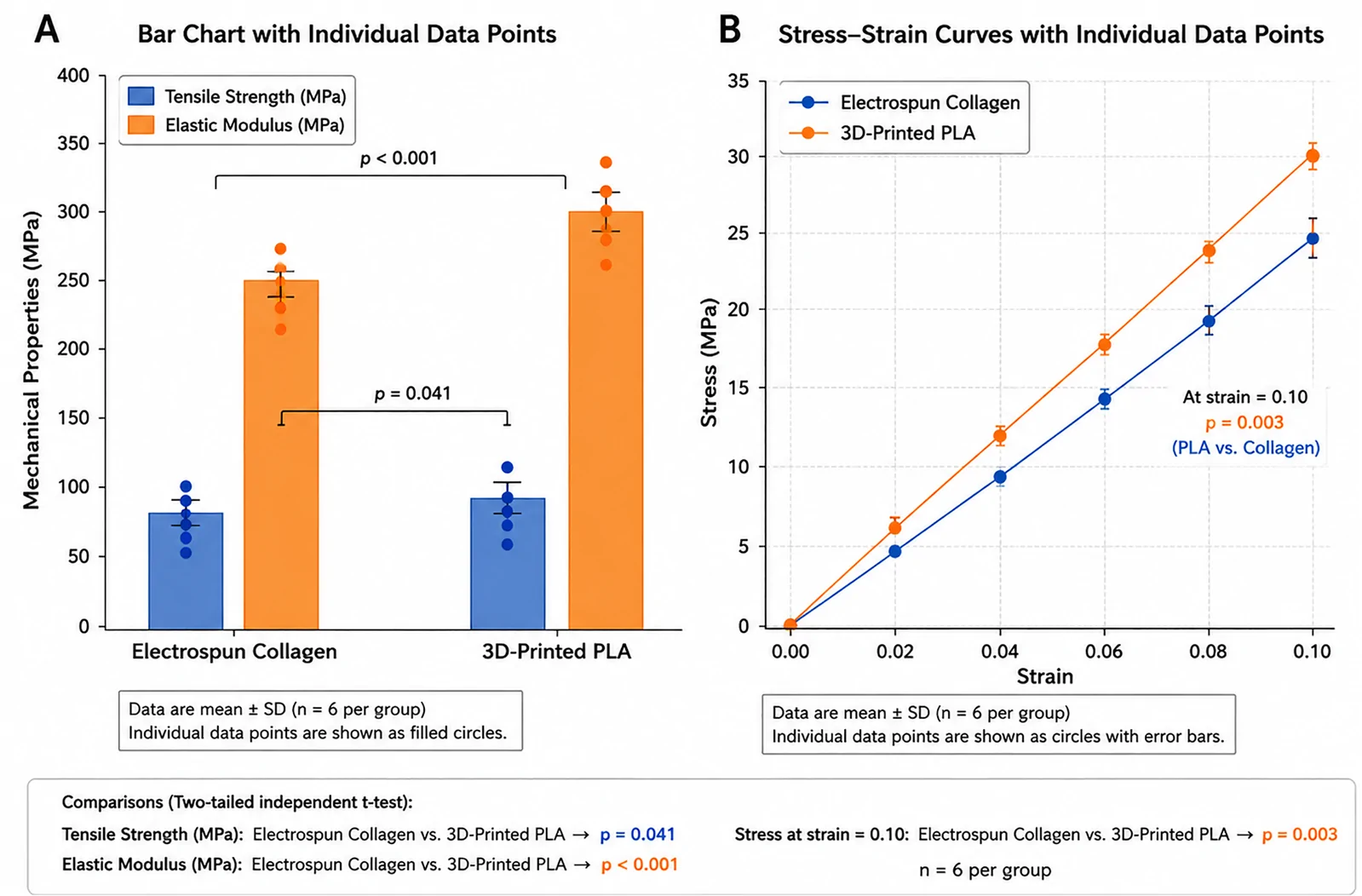
Mechanical properties of preconditioned vs. non-preconditioned scaffolds. (A) Tensile strength and elastic modulus (mean ± SD) of electrospun collagen and 3D-printed PLA scaffolds. PLA scaffolds exhibit slightly higher mechanical strength and stiffness compared to collagen scaffolds. (B) Stress–strain curves illustrating the elastic behavior of both scaffold types, with PLA demonstrating a steeper slope as an indication of higher modulus. These findings confirm that both scaffolds possess sufficient mechanical integrity for tendon regeneration, with minor variations in biomechanical performance. Scaffold samples used n = 5 per condition. Unpaired t‑test with statistical significance at p < 0.05 (*p < 0.05, **p < 0.01, and ***p < 0.001).

The 3D-printed PLA scaffolds displayed higher baseline mechanical properties, with a tensile strength of 90 ± 15 MPa and elastic modulus of 300 ± 25 MPa, reflecting greater stiffness and load-bearing capacity. Under cyclic preconditioning, PLA scaffolds maintained comparable mechanical performance with preserved tensile strength (85 MPa) and elastic modulus (245 MPa), suggesting excellent fatigue resistance. Contrary to the aligned fibers of electrospun collagen scaffolds, where PLA scaffolds had randomly arranged fibers (pore size 75–120 μm), which offer distinct mechanical characteristics and biological interactions that may influence cellular behavior and fluid dynamics differently.

Stress-strain curve analysis (Fig 4B) revealed that electrospun collagen scaffolds showed a more gradual deformation pattern with lower peak stress (about 30 MPa at 0.10 strain), characteristic of a compliant, viscoelastic material suitable for dynamic tendon environments. Conversely, 3D-printed PLA scaffolds demonstrated a steeper initial slope (higher elastic modulus) and reached higher stress values at equivalent strain levels, indicating a stiffer or more elastic response. Both scaffold types maintained adequate mechanical integrity for tendon regeneration following preconditioning, with preconditioned collagen scaffolds showing improved strain tolerance, a critical feature for withstanding post-implantation physiological loading. These findings confirm that electrospinning and 3D-printing techniques can produce scaffolds with tunable structural and biomechanical properties designed to encourage tendon regeneration.

### Stem cell integration (in vitro)

#### Cell viability and proliferation.

Fig 5 shows the temporal variations in scaffold construct cell viability over a 14-day period based on the MTT assay (570 nm) in six experimental groups. All scaffold-based groups saw a gradual increase in cell viability over time, with notable variations based on the degree of mechanical and biological augmentation. At Day 3, the G1 and G5 showed considerably lower absorbance values (p < 0.01), but the G4 and G6 showed the highest values, indicating higher initial cell viability. By Day 5, the biological component groups, G2 and G3, showed a significant increase in absorbance compared to G1 (p < 0.001), with G4 continuing to have the best survivability of all the experimental groups. This pattern continued on Day 7, with G3 and G4 exhibiting superior improvements in cell viability. With statistically significant (p < 0.05) variations from baseline scaffold conditions, the improvement in these groups is indication of the synergistic effects of growth factor administration and mechanical stimulation. The G4 attained the maximum (p < 0.001) absorbance by Day 14, surpassing all other groups and becoming close to the G6. However, G1 and G5, continued to be relatively low, indicating restricted biological activity in the absence of cellular and biochemical stimuli. Overall, these findings show that scaffold-associated cell viability increased steadily from early to late time points, with the 6ompletely designed constructions showing the prime gain.

**Fig 5:**
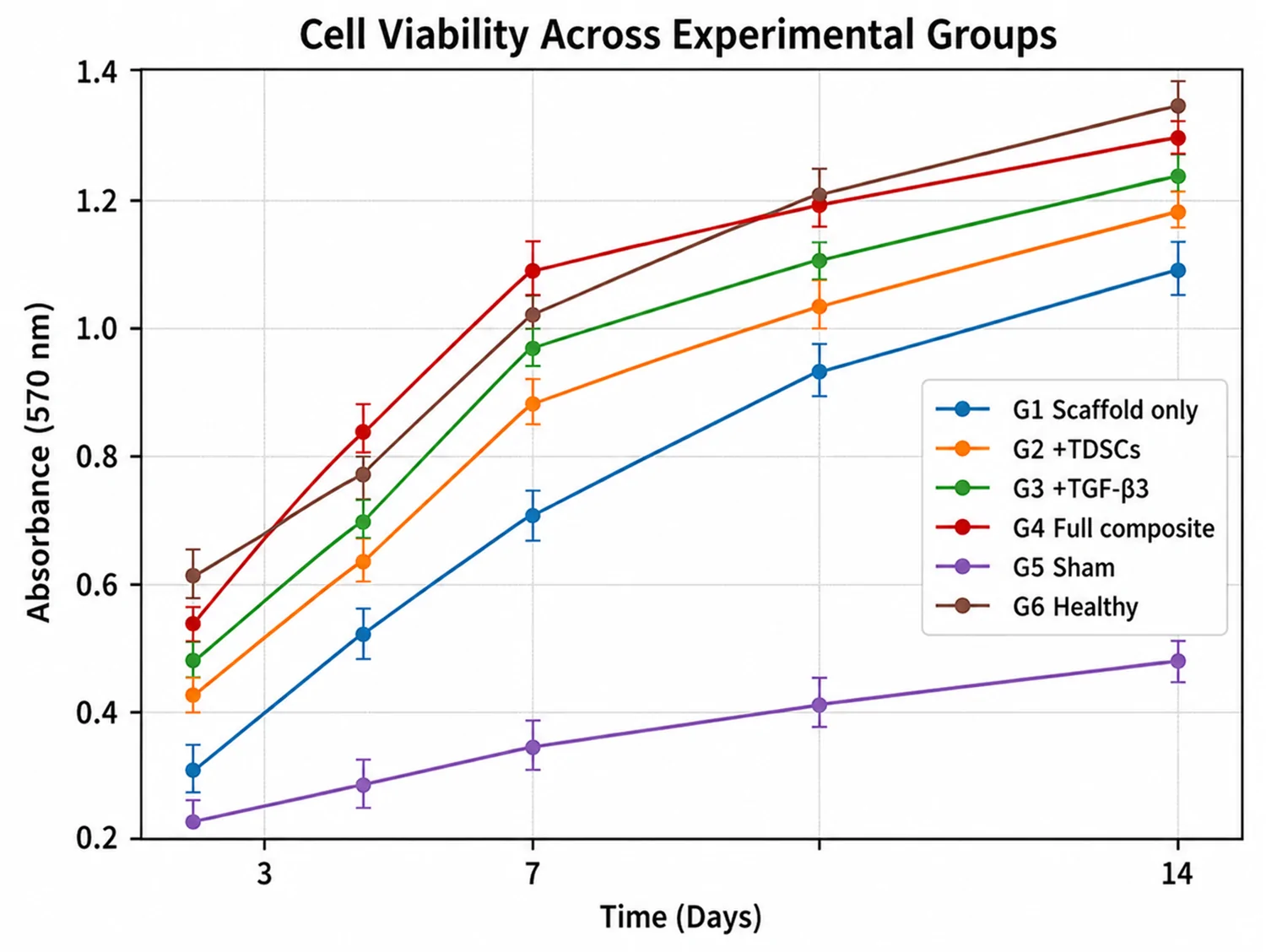
Cell viability of scaffold constructs assessed by MTT assay (570 nm) over 14 days across six experimental groups. The full composite group (G4: scaffold + TDSCs + TGF-β3 + mechanical preconditioning) demonstrated the highest increase in cell viability, approaching healthy control levels (G6) by Day 14. Groups incorporating biological and biochemical cues (G2–G4) showed enhanced viability compared to scaffold-only (G1) and sham control (G5). Note: * significant @ 0.05, ** significant @ p < 0.01 Cell viability of scaffold constructs assessed by MTT assay (570 nm) over 14 days across six experimental groups. The full composite group (G4: scaffold + TDSCs + TGF-β3 + mechanical preconditioning) demonstrated the highest increase in cell viability, approaching healthy control levels (G6) by Day 14. Groups incorporating biological and biochemical cues (G2–G4) showed enhanced viability compared to scaffold-only (G1) and sham control (G5). Note: Mice used n = 12 per group. One‑Way ANOVA with statistical significance at p < 0.05 (*p < 0.05, **p < 0.01, and ***p < 0.001).

Live/Dead fluorescence imaging revealed a steady increase in cell density throughout the 14-day culture period in all scaffold-based groups (Fig 6A). High cell viability and general cytocompatibility of the scaffold systems are indicated by a preponderance of green fluorescence (Calcein-AM) with little red staining (propidium iodide). Day 14 saw the formation of dense, evenly spaced layers of cells, especially in clusters that included biological and mechanical stimuli. The G4 showed the highest level of cell proliferation and spatial homogeneity among the testing conditions. G3 and G2 came next, showing a distinct stepwise increase in cellular responsiveness with the addition of cellular components and growth factor stimulation. On the other hand, the G5 showed no cellular activity during the research, while the G1 demonstrated restricted cell attachment and delayed growth. As a physiological reference, the G6 continuously maintained high viability. These findings were supported by quantitative analysis (Fig 6B), which showed that the number of cells increased over time in each group. Interestingly, G4 showed the greatest proliferation, peaking at Day 14 (p < 0.01), followed by G3 and G2 (p < 0.05). On the other hand, G1 and G5 have only little increased over time. These findings show that, in comparison to scaffold alone, the addition of TDSCs, TGF-β3, and mechanical stimulation greatly increases cell proliferation.

**Fig 6:**
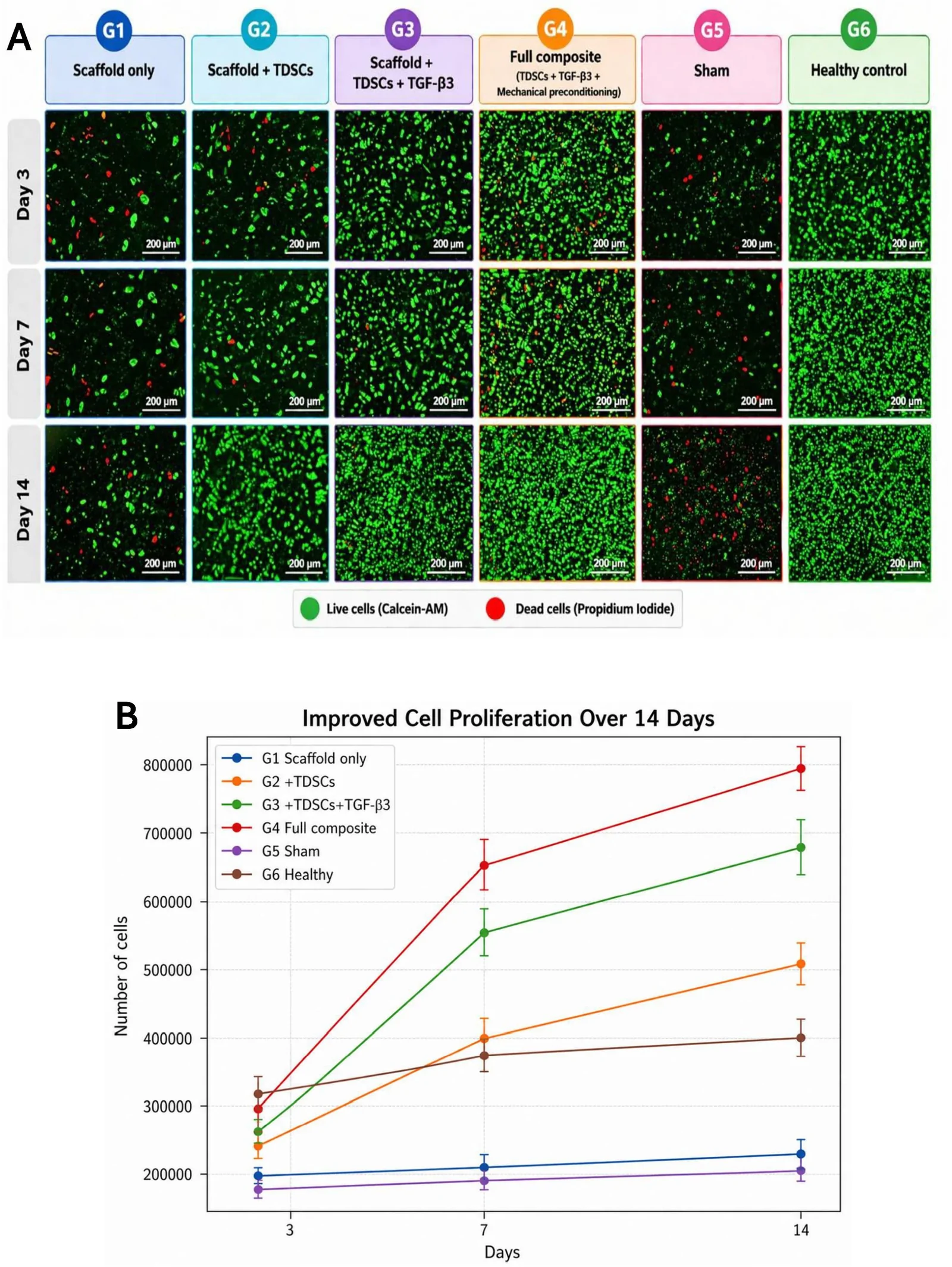
Spatiotemporal evaluation of cell viability and proliferation across six biologically relevant experimental groups over 14 days. (A) Representative fluorescence microscopy images of Live/Dead-stained cells cultured on scaffold constructs at Days 3, 7, and 14. Experimental groups include: G1 (scaffold only), G2 (scaffold + TDSCs), G3 (scaffold + TDSCs + TGF-β3), G4 (full composite: scaffold + TDSCs + TGF-β3 + mechanical preconditioning), G5 (sham), and G6 (healthy control). Live cells are stained green using Calcein-AM, while dead cells are stained red using propidium iodide (PI). Progressive increases in cell density and viability are observed from G1 to G4, with the full composite group (G4) showing the highest cell confluency and minimal cell death over time. The sham group (G5) shows limited cellular activity, while the healthy control (G6) maintains high baseline viability. (B) Quantitative analysis of cell proliferation over 14 days. The number of cells increased in a time-dependent manner across all groups, with G4 exhibiting the most pronounced proliferation, followed by G3 and G2, indicating interactive effects of stem cells, growth factor delivery, and mechanical stimulation. G1 and G5 showed minimal proliferation, while G6 reflects physiological baseline levels. Data are presented as mean ± standard deviation (SD) (n ≥ 3). All Magnification scales = 200x unless otherwise indicated. Fluorescence imaging was performed using a Zeiss LSM 980 microscope equipped with Airyscan 2. Live-cell imaging was conducted using FluoroBrite DMEM, while fixed samples were prepared in phosphate-buffered saline (PBS) containing 4% paraformaldehyde (PFA). ProLong Gold Antifade reagent was used to minimize photobleaching during imaging. Mice used n = 12 per group. One‑Way ANOVA with statistical significance at p < 0.05 (*p < 0.05, **p < 0.01, and ***p < 0.001).

Fig 7 shows the relationship between cell viability and proliferation over a 14-day culture period, indicating a coordinated and time-dependent enhancement in cellular performance. Cell viability increased from approximately 86% at Day 7 to about 94% by Day 14, indicating sustained improvement in cell health and metabolic activity within the scaffold constructs. Concurrently, there was a noticeable rise in cell proliferation, which by Day 14 had nearly tripled. This simultaneous increase in viability and proliferation indicates that the culture conditions successfully supported both active cell division and cell survival. Notably, a favorable milieu that fosters cellular development without sacrificing cell integrity is highlighted by the substantial positive relationship between viability and proliferation. These results demonstrate that the designed system offers favorable conditions for long-term cell proliferation, functional activity, and general biological performance.

**Fig 7:**
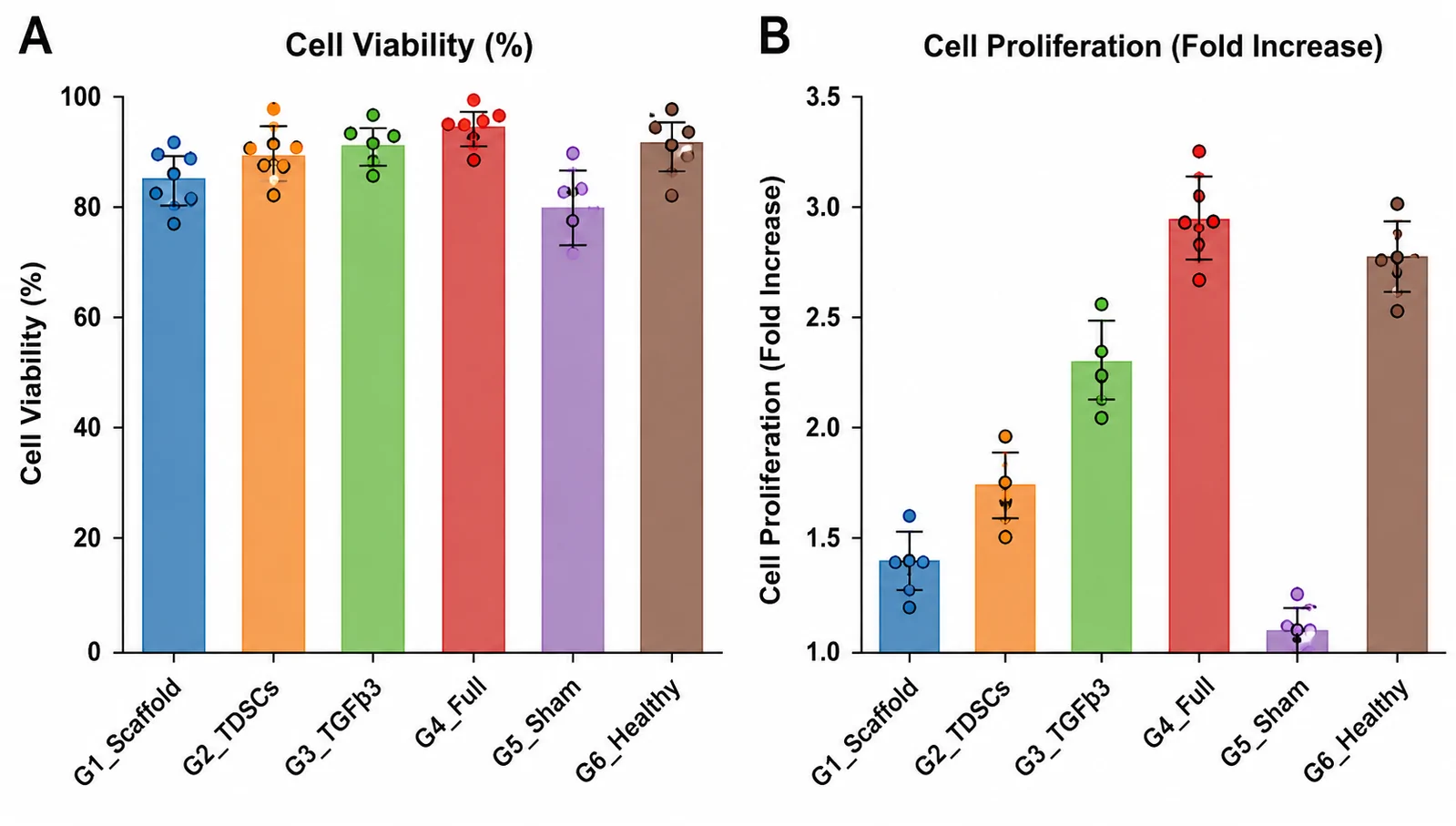
Cell viability and proliferation of stem cell-seeded scaffolds after 14 days of culture. (A) Cell viability (%) determined by the MTT assay. (B) Cell proliferation expressed as fold increase relative to Day 1. Data are presented as mean ± SD with individual biological replicates overlaid (n = 6). Statistical significance was determined using one-way ANOVA followed by Tukey’s multiple comparisons test.. G1 (scaffold only), G2 (scaffold + TDSCs), G3 (scaffold + TDSCs + TGF-β3), G4 (full composite: scaffold + TDSCs + TGF-β3 + mechanical preconditioning), G5 (sham control), and G6 (healthy control).

### Differentiation and ECM production

Fig 8 shows strong stem cell tenogenic differentiation and significant ECM formation in scaffold constructs at Day 14. Based on quantitative analysis of tendon-specific transcriptional markers, scleraxis expression increased almost five folds and tenomodulin expression increased four folds in comparison to controls. The effective commitment of stem cells toward a tenocyte-like phenotype is confirmed by the enhanced expression of these markers, which are essential regulators of tendon development and maturation. Significant collagen type I deposition (45 ± 5 µg/mg scaffold) and increased GAG content (25 ± 3 µg/mg scaffold) indicate that ECM production was significantly increased concurrently. Increased GAG levels show active matrix remodeling and hydration capacity, whereas collagen type I, the main structural protein in tendon tissue, represents the development of a mechanically competent matrix. Remarkably, the concurrent overexpression of ECM components and tenogenic markers points to a closely related process of cellular differentiation and functional matrix building. This synchronized reaction reveals how well the tailored milieu directs lineage-specific differentiation while simultaneously encouraging the formation of a tendon-like matrix that is both structurally and physiologically relevant.

**Fig 8:**
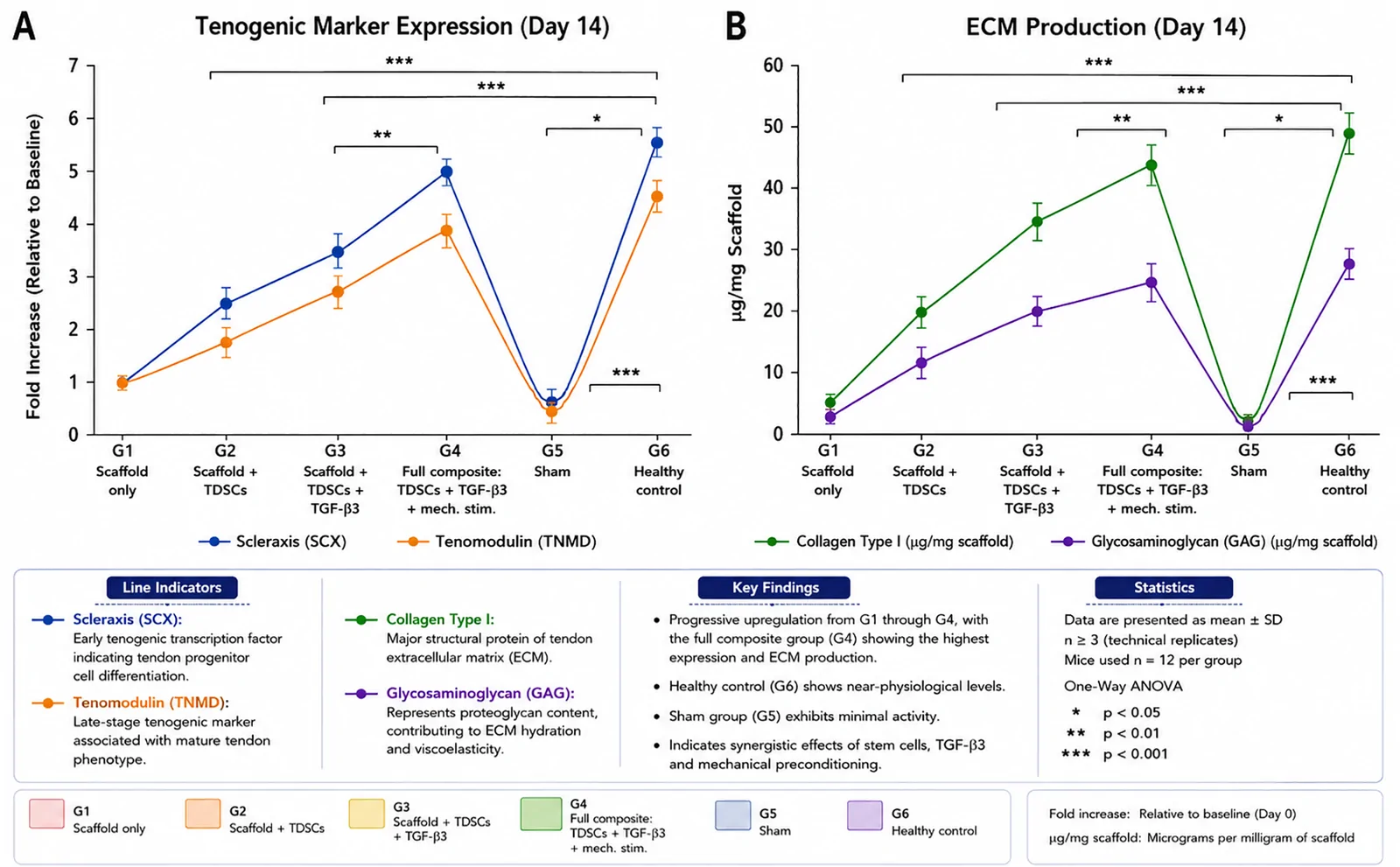
Tenogenic differentiation and extracellular matrix production after 14 days of culture. (A) Quantitative expression of tendon-specific markers determined by RT-qPCR. The blue line represents Scleraxis (SCX), an early tenogenic transcription factor, while the orange line represents Tenomodulin (TNMD), a marker of mature tendon differentiation. (B) Quantification of extracellular matrix production, including Collagen Type I (green) and Glycosaminoglycan (GAG; purple). Data are presented as mean ± SD (n = 12). Generally, the results show a clear stepwise enhancement in both differentiation and matrix synthesis, signifying the interactive effects of stem cells, growth factor delivery, and mechanical stimulation. Statistical analysis was performed using one-way ANOVA followed by Tukey’s post hoc test.

SEM examination showed a distinct, time-dependent progression in ECM production and fiber structure during the 14-day growth period in all experimental groups (Fig 9). On Day 3, the seeded groups (G2–G4) displayed early cell attachment and initial ECM bridging across fibers, indicating active cell–scaffold interactions, but the G1 revealed a mostly porous architecture with negligible surface deposition. Significant variations across the groups became apparent by Day 7. With G3 showed higher ECM accumulation and early indications of fiber alignment, while G2 revealed increased matrix deposition with partially interconnected collagen networks. Interestingly, the fibrillar structure of the G4 was noticeably denser and more ordered, indicating that mechanical preconditioning and growth factor delivery have complementary effects on matrix organization.

**Fig 9:**
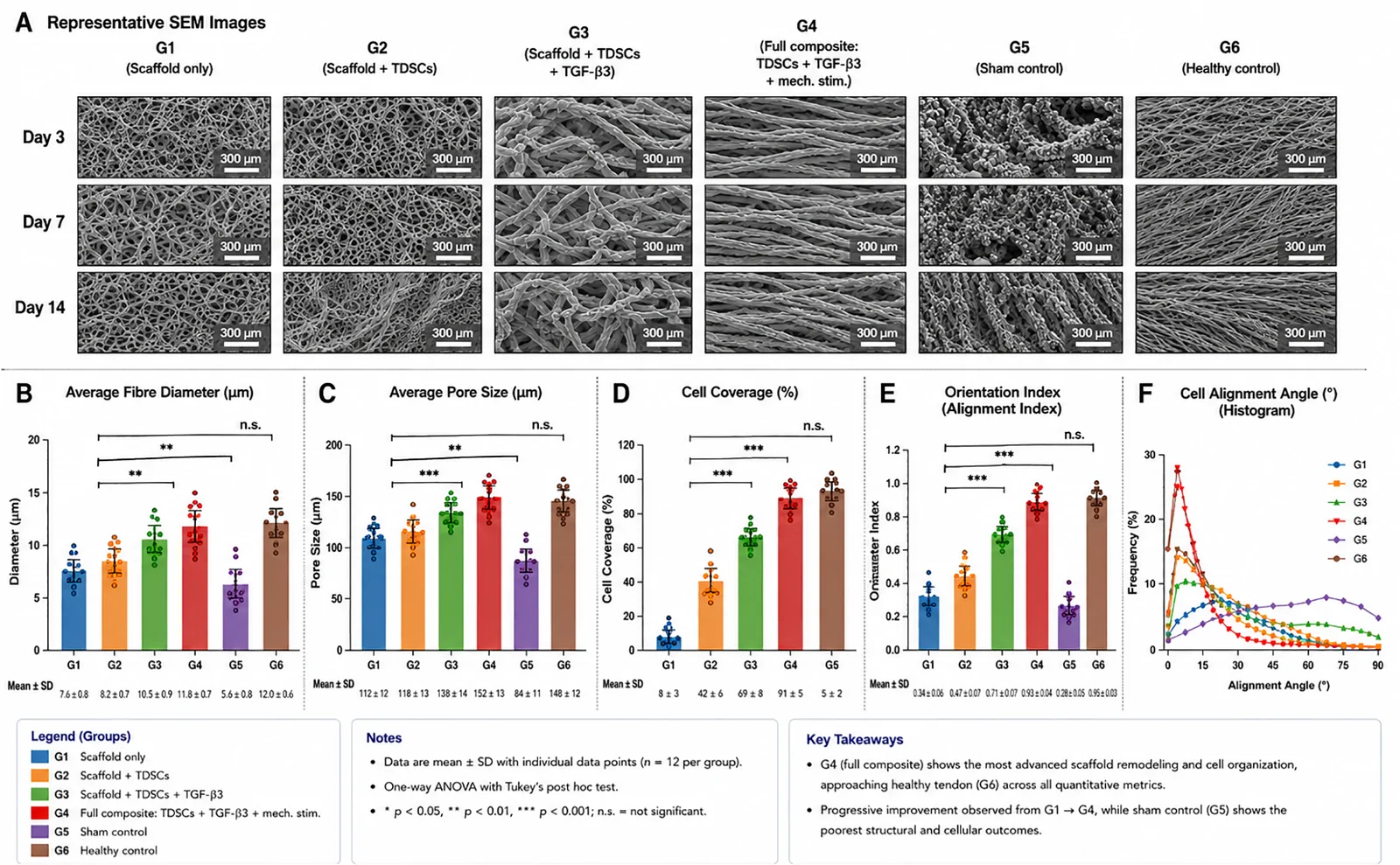
Quantitative morphometric analysis of scaffold remodeling and cell organization using scanning electron microscopy (SEM) micrographs (A) demonstrate progressive extracellular matrix (ECM) deposition, collagen fiber maturation, and fiber alignment from Day 3 to Day 14 across the six experimental groups. The analysis revealed significant increases in average fiber diameter (B), average pore size (C), cell coverage (D), and fiber orientation index (E) in the full composite group (G4: scaffold + TDSCs + TGF-β3 + mechanical preconditioning), with values approaching those observed in the healthy control (G6). Cell alignment angle distribution (F) further demonstrated a narrow orientation profile in G4, indicating highly aligned cellular organization comparable to native tendon, whereas the scaffold-only (G1) and sham control (G5) groups exhibited more random cell orientation. Mice used n = 12 per group. One‑Way ANOVA followed by Tukey’s post hoc multiple-comparison test with statistical significance at p < 0.05 (*p < 0.05, **p < 0.01, and ***p < 0.001) and n.s., not significant. Scale bar = 300 μm.

Day 14 ECM maturation was most noticeable in G4, which had strongly aligned, densely packed collagen fibrils that resembled the architecture of genuine tendons. With G2 showed continuous but less organized ECM growth, while G3 showed moderate fiber organization with increased matrix density. Conversely, G1 and G5 showed very small structural alteration and maintained a sparse or chaotic matrix architecture, indicating that there was no notable cell-mediated remodeling. Compact, anisotropic collagen organization was continuously seen in the G6

### Biofunctionalization

During the 21-day observation period, the release kinetics of TGF-β3 showed a distinct time-dependent and group-specific profile (Fig 10). As expected, very little release was seen in the control groups, which included G1, G5, and G6. This validated baseline conditions and confirmed that no growth factor was added. The low release levels of G2 probably indicate indirect cellular interactions rather than actual growth factor delivery. However, G3 had a sustained and progressive release pattern, suggesting that TGF-β3 was successfully incorporated within the scaffold matrix and that diffusion was controlled over time. With a consistent rise in TGF-β3 concentration throughout the study period, the G4 showed the most noticeable and persistent release profile. This improved release behavior implies that scaffold architecture, microsphere integration, and mechanical preconditioning all functioned together to optimize growth factor retention and control release kinetics.

**Fig 10:**
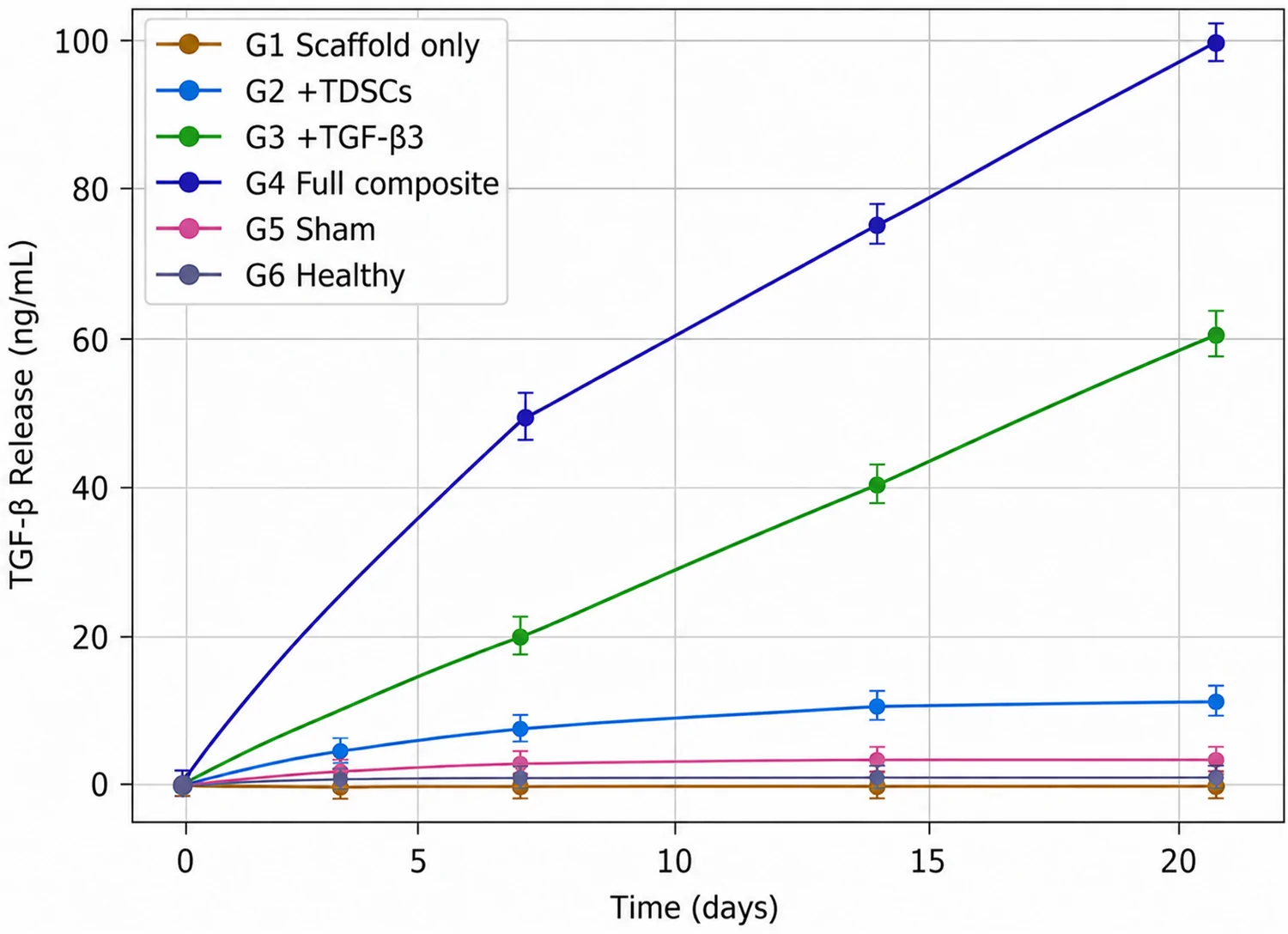
Time-dependent release profile of TGF-β3 from scaffold constructs across six experimental groups (G1–G6) over 21 days. Minimal or negligible release is observed in control groups (G1, G5, and G6), while G2 shows limited release associated with cellular activity. In contrast, G3 (scaffold + TDSCs + TGF-β3) demonstrates a sustained release pattern, which is further enhanced in G4 (full composite), displaying the highest and most prolonged release profile. These results confirm effective incorporation and controlled delivery of TGF-β3, with mechanical preconditioning contributing to improved release kinetics. Mice used n = 12 per group. One‑Way ANOVA with statistical significance at p < 0.05 (*p < 0.05, **p < 0.01, and ***p < 0.001).

### In vivo assessment

Fig 11 displays the results of the in vivo study of implanted scaffolds in a rat model. The control groups (G5 and G6) show normal or poor recovery, with G5 demonstrating persistent tissue damage and disorganized collagen fibers across the 28‑day period. With obvious inflammation, insufficient remodeling, and persistently high adhesion levels, G1 exhibits the worst healing out of all the experimental groups. G2 demonstrates better healing with progressively decreased inflammation and continuous tissue remodeling, but by Day 28, collagen alignment is only relatively ordered. In contrast, G4 displays the most significant progress, characterized by well‑organized tissue structure, minimal inflammation, well‑aligned collagen fibers, and the highest histological scores throughout Days 7, 14, and 28, indicating accelerated regeneration and superior ECM remodeling. G5 shows outcomes comparable to G4 but with slightly less mature collagen alignment and mild residual disorganization by Day 28.

**Fig 11:**
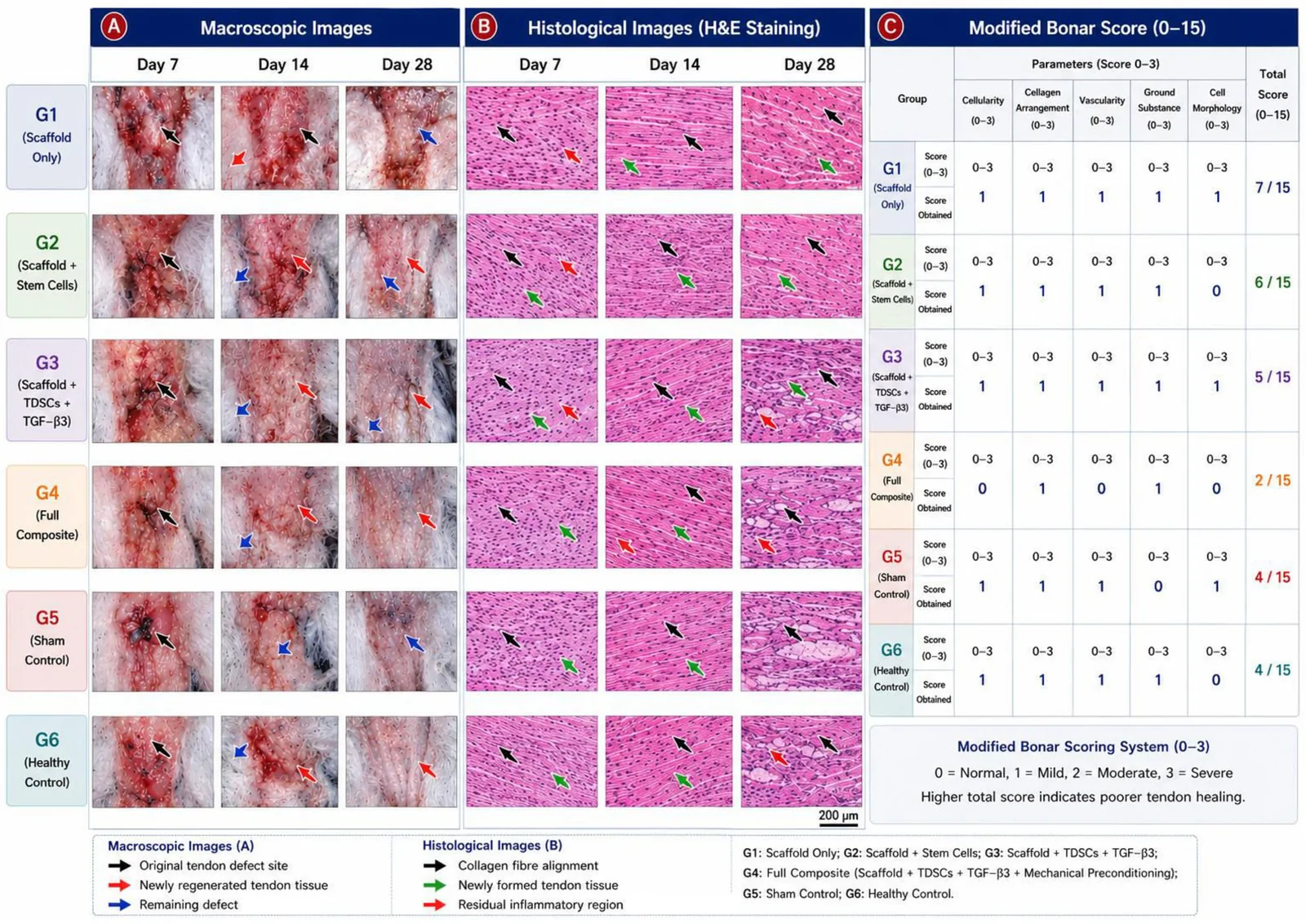
In vivo evaluation of tendon regeneration following implantation of engineered composite scaffolds in a rat Achilles tendon defect model. (A) Representative macroscopic images of tendon repair sites at Days 7, 14, and 28 post-implantations across experimental groups. The scaffold-only group (G1) demonstrates partial defect filling with gradual reduction in inflammation but limited structural integration. The scaffold + TDSCs group (G2) displays improved tissue continuity and reduced inflammation over time. The scaffold + TDSCs + TGF-β3 group (G3) shows enhanced healing, with more uniform tissue appearance and progressive defect bridging. The full composite group (G4: scaffold + TDSCs + TGF-β3 + mechanical preconditioning) displays the most advanced regeneration, characterized by near-complete defect closure and restored tendon-like morphology by Day 28. The sham control (G5) shows moderate healing with incomplete remodeling, while the healthy control (G6) maintains intact tendon structure. (B) Representative hematoxylin and eosin (H&E)-stained histological sections corresponding to each group and time point. The control group exhibits disorganized collagen fibers, high cellular infiltration, and persistent inflammatory response. The scaffold-only group shows increased cellularity and early matrix deposition but limited collagen alignment. The scaffold + TDSCs group demonstrates improved extracellular matrix formation with moderate collagen organization. The scaffold + TDSCs + TGF-β3 group reveals enhanced collagen deposition and reduced inflammatory cells, indicating active remodeling. The full composite group presents densely packed, well-aligned collagen fibers resembling native tendon architecture, with minimal inflammation by Day 28. The sham group shows partial collagen organization, but inferior structural integrity compared to scaffold-treated groups, while the healthy control displays highly organized, parallel collagen bundles with normal tendon histoarchitecture. (C) Semi-quantitative assessment using the modified Bonar scoring system. Lower scores indicate improved tendon healing. Data are presented as mean scores from blinded observer. *Scale bars:* 200 µm for histology. Data are representative of n = 6 rats per group per time point. Note: Brightfield microscopy model with H&E staining was used and sections mounted with DPX medium. Mice used n = 6 per group per time point.

An evaluation of the extracted tendons revealed clear differences in tissue form under different imaging conditions (Fig 12a–12c). In situ examination showed a clearly defined repair region with preserved anatomical continuity and no evidence of re-rupture, implying successful joining of the tendon defect ((Fig 12a). The regenerated tendons maintained structural continuity and hydration after explantation, appearing cohesive and well-integrated with the surrounding native tissue (Fig 12b). These findings were further supported by cross-sectional examination, which revealed an internal architecture at the repair site, which was dense, compact, and generally uniform. This architecture closely resembled the morphology of native tendons and suggested successful ECM deposition and tissue consolidation (Fig 12c).

**Fig 12:**
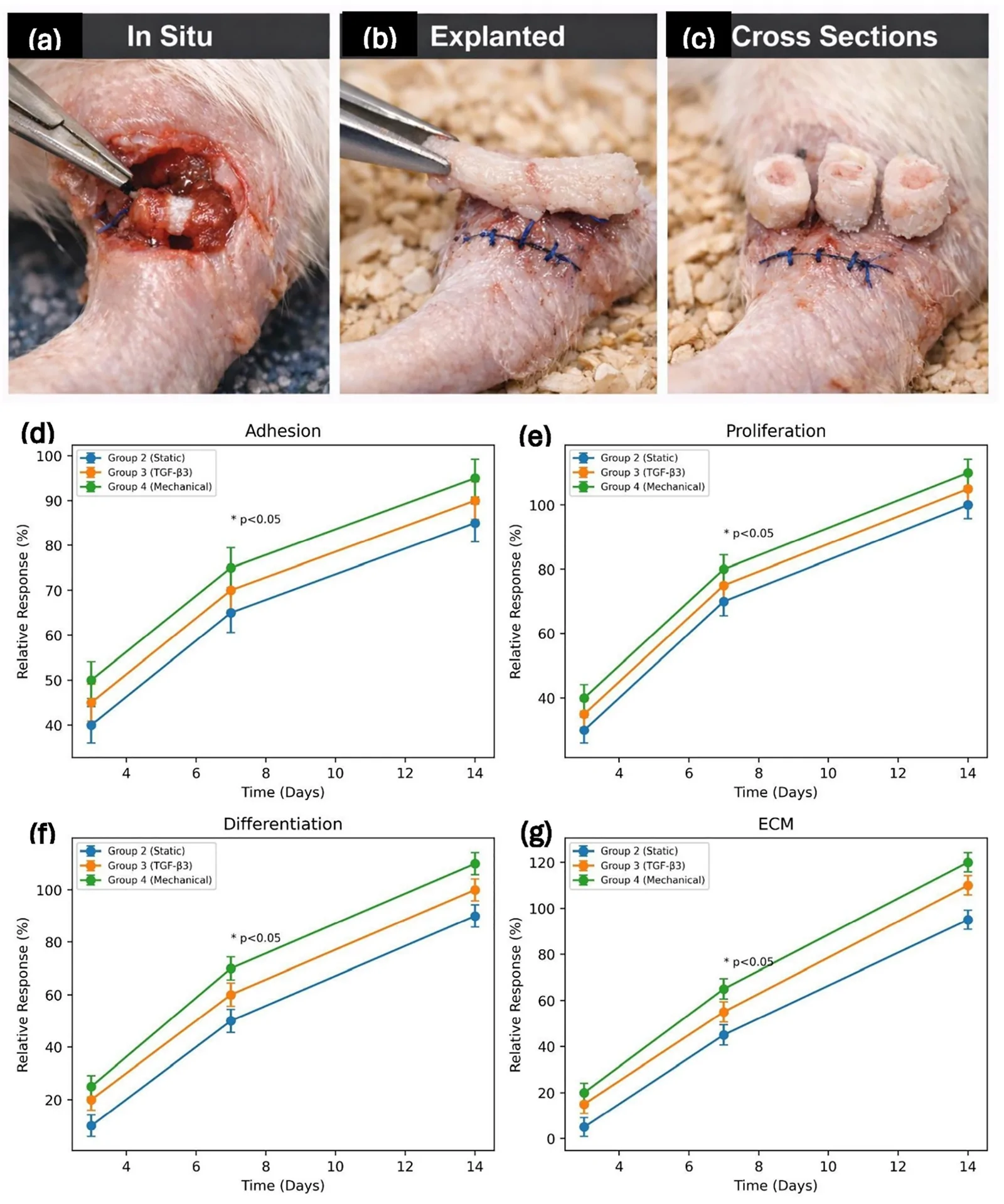
Gross images of harvested tendons under standardized imaging conditions. (a) In situ view of the Achilles tendon immediately post-euthanasia, showing a well-defined repair site with preserved anatomical alignment and no evidence of re-rupture. (b) Exexplanted tendon demonstrating a continuous, cohesive tissue structure with an integrated healing zone. (c) Cross-sectional view of the repaired tendon showing a dense, compact, and relatively homogeneous internal architecture, indication of successful tissue consolidation. (d) Quantitative analysis of adhesion formation over 14 days, showing a time-dependent increase, with the G4 exhibiting significantly higher responses compared to G2 and G3 (*p < 0.05). (e) Cell proliferation assay revealing progressive increases across all groups, with G4 showing the highest proliferative activity (*p < 0.05). (f) Tenogenic differentiation response over time, with maximal differentiation observed in G4, indicating synergistic effects of mechanical stimulation and TGF-β3. (g) Extracellular matrix (ECM) production, including collagen and glycosaminoglycan deposition, showing a significant time-dependent increase, with G4 achieving the highest levels (*p < 0.05). Data are presented as mean ± SD. Mice used n = 6 per group per time point. One‑Way ANOVA with statistical significance at p < 0.05 (*p < 0.05, **p < 0.01, and ***p < 0.001).

From Day 3 to Day 14, quantitative functional evaluations showed a steady, time-dependent improvement in biological performance (Fig 12d–12g). All groups saw a progressive increase in adhesion formation, but the mechanically preconditioned group (G4) showed significantly higher adhesion responses than the static (G2) and TGF-β3-treated groups (G3) (*p < 0.05), suggesting better scaffold integration and interaction with neighbouring tissue (Fig 12d). Comparably, cell proliferation increased significantly over time, with G4 continuously exhibiting the maximum ability for proliferation, followed by G3 and G2 (*p < 0.05; Fig 12e).

The synergistic effects of mechanical stimulation and persistent growth factor supply were seen in tenogenic differentiation, where G4 attained the highest overexpression of differentiation markers (Fig 12f). All groups saw a significant increase in ECM production, including collagen and GAG synthesis, with G4 showing the highest levels (*p < 0.05; Fig 12g). Macroscopic and cross-sectional analyses showed increased structural organization in tandem with this gradual improvement in ECM deposition.

At 12 weeks, histological analysis revealed significant variations in tendon repair across the experimental groups (Fig 13). In G1 and G5, H&E staining (Fig 13A) showed poor structural integrity, chronic inflammatory infiltration, and disordered tissue architecture. However, G2 and G3 displayed better tissue structure, with fewer inflammatory characteristics and more elongated and aligned fibroblast-like cells. Interestingly, G4 showed the most superior tissue architecture, with highly ordered cellular alignment and no inflammatory infiltration, and it resembled native tendon shape. G6 showed the predicted normal tendon structure.

**Fig 13:**
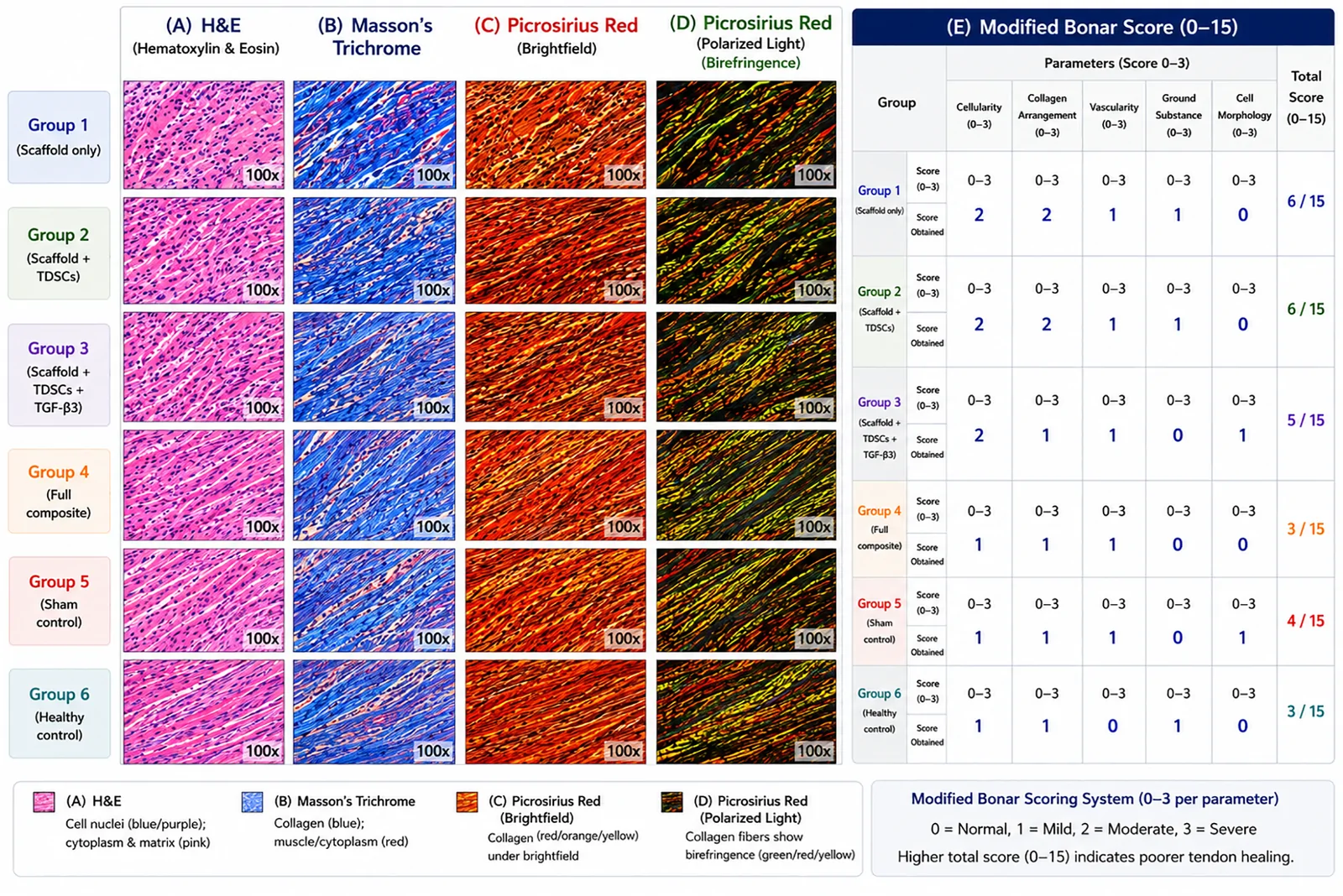
Histological evaluation of tendon repair at 12 weeks. (A) Hematoxylin and eosin (H&E) staining showing overall tissue morphology and cellular organization. (B) Masson’s Trichrome staining showing collagen deposition (blue) and cytoplasm/muscle components (red). (C) Picrosirius Red staining under brightfield demonstrating collagen fiber density and orientation. (D) Picrosirius Red staining under polarized light highlighting collagen birefringence and fiber maturity. G1 (scaffold only) displayed poorly organized tissue architecture, high cellular infiltration, and discontinuous, loosely arranged collagen fibers, indicating limited regenerative capacity. G2 (scaffold + TDSCs) showed improved cellular organization and increased collagen deposition, with partial alignment of collagen fibers along the tendon axis. G3 (scaffold + TDSCs + TGF-β3) showed further enhancement in matrix organization, with denser collagen deposition and more pronounced fiber alignment compared to G1 and 2. G4 (full composite) displayed the most advanced regeneration, characterized by well-organized, densely packed, and longitudinally aligned collagen fibers resembling native tendon structure, along with reduced cellularity. G5 (sham control) showed irregular tissue structure with fibrotic healing, moderate collagen deposition, and limited fiber alignment, indicative of scar-mediated repair. G6 (healthy control) exhibited normal tendon histoarchitecture, with highly aligned collagen bundles, low cellularity, and well-defined extracellular matrix organization. Under polarized light, G1 and 5 predominantly displayed green to yellow birefringence, indication of immature and disorganized collagen fibers. Gs 2 and 3 showed mixed green–orange birefringence, reflecting intermediate collagen maturation and partial fiber alignment. In contrast, Group 4 demonstrated strong red–orange birefringence comparable to Group 6, indicating highly mature, well-organized collagen fibers. All Magnification scales = 100x unless otherwise indicated.

Variations in collagen deposition were further emphasized by Masson’s Trichrome staining (Fig 13B). Collagen fibers were scarce, broken, and disorganized in G1 and G5. Increased collagen content and partial alignment along the tendon axis were seen in G2 and G3, suggesting continuous matrix remodeling. In contrast, G4 displayed collagen bundles that were dense, continuous, and well-aligned, resembling the structural organization seen in Group 6, the healthy control.

These results were validated by picrosirius red staining under brightfield (Fig 13C). Some heterogeneity persisted, with G2 and G3 showed more consistent and abundant collagen deposition than G1 and G5. On the other hand, G4 showed highly ordered and tightly packed collagen fibers with a distribution pattern that was quite similar to that of genuine tendon tissue.

Collagen maturity and organization were clearly different under polarized light microscopy (Fig 13D). Indication of thin, immature, and disordered collagen fibers, G1 and G5 displayed mild birefringence dominated by green tones. Stronger birefringence with a mixture of green-yellow to orange-red fibers was seen in G2 and G3, indicating improved alignment and progressive collagen maturation. Extensively, G4 showed strong birefringence with a prominent reddish-orange hue, indicating thicker, more developed, and highly aligned collagen fibers that were similar to those in the G6.

Quantitative analysis of collagen alignment using Fourier transform-based orientation analysis (Fig 14) supported these qualitative observations, with G2 and G3 showing moderate increases in alignment index, while G4 demonstrated the highest alignment among the treatment groups, approaching that of native tendon.

**Fig 14:**
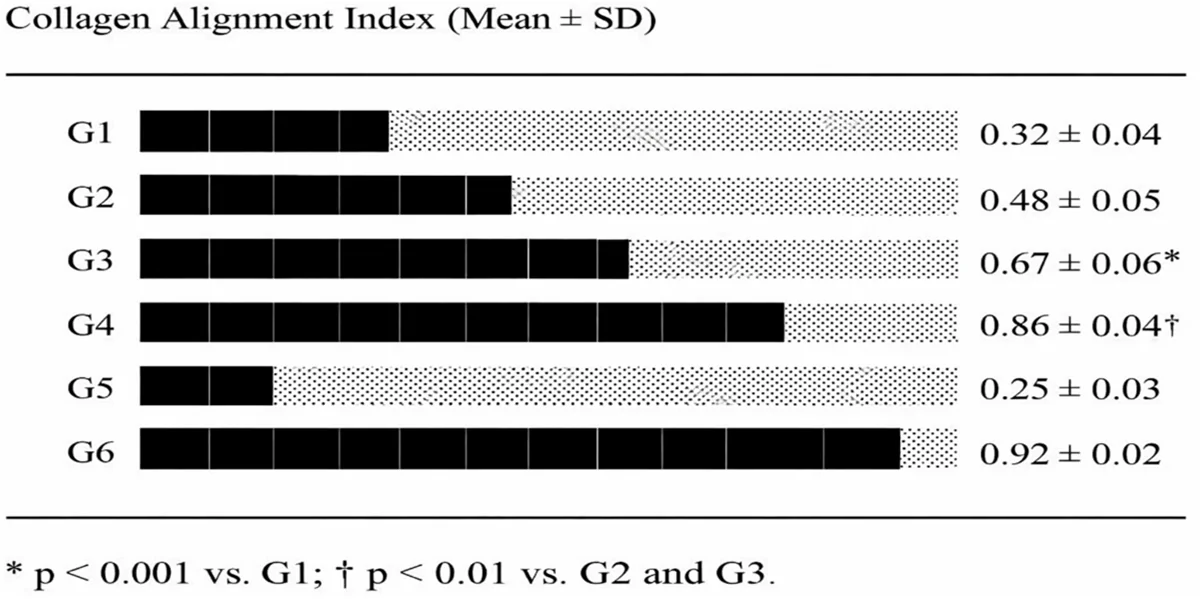
Collagen alignment index at 12 weeks. G1 (scaffold only, acellular), G2 (scaffold + TDSCs) received scaffold seeded with tendon-derived stem cells. G3 (scaffold + TDSCs + TGF-β3) received scaffold seeded with TDSCs and embedded with TGF-β3-loaded PLGA microspheres. G4 (full composite) received scaffold seeded with TDSCs, embedded with TGF-β3 microspheres, and subjected to cyclic mechanical preconditioning (5% strain, 1 Hz, 4 hours/day for 7 days) prior to implantation. G5 (sham control) underwent surgical tendon defect creation with primary suture repair without scaffold implantation. G6 (healthy control) consisted of uninjured, unoperated rats with intact Achilles tendons to define native mechanical and histological baselines. Mice used n = 6 per group. One‑Way ANOVA with statistical significance at p < 0.05 (*p < 0.05, **p < 0.01, and ***p < 0.001).

### Immunohistochemistry

At 12 weeks, tendon maturation, matrix composition, and inflammatory response were quantitatively evaluated for each group by immunohistochemical staining (Fig 15, Table 1). Both regenerative and inflammatory markers showed distinct group-dependent variations. Tenomodulin expression, a marker of tenocyte maturation, increased gradually in all groups and peaked in G4 (212 ± 18), which was significantly higher than G1 and G3 (p < 0.001) and reached around 90% of native tendon levels (G6: 235 ± 15). Collagen type I, the main component of mature tendon matrix, showed a similar trend, with G4 (245 ± 20) closer to G6 (268 ± 18). Conversely, collagen type III was significantly lower in G4 (54 ± 6) than in G1, G2, and G5 and is linked to early-stage or fibrotic healing. Consequently, G4 had a considerably higher collagen I/III ratio (4.54 ± 0.35) than G3 (2.61 ± 0.22), G1 and G5 (p < 0.001), and native tendon (6.38 ± 0.42). This is a crucial measure of matrix quality and maturity. Concurrently, CD68-positive macrophage infiltration showed an inverse trend, with G1 and G5 showing the greatest levels (128 ± 14 and 145 ± 18, respectively) and G4 showing a significant decline (28 ± 5), which is roughly 78% less than G1. This decrease suggests that the G4’s inflammatory response has been significantly reduced. Compared to G1 and G5, G2 and G3 showed moderate increases in tenomodulin and collagen I expression, a partial decrease in collagen III, and a decrease in macrophage infiltration. Nevertheless, these improvements were still less than those shown in G4.

**Table 1:** Quantitative immunohistochemical analysis at 12 weeks.

Group	Description	Tenomodulin	Collagen I	Collagen III	Collagen I/III Ratio	CD68 (Macrophages)
G1	Scaffold only (Acellular)	45 ± 8	82 ± 10	78 ± 9	1.05 ± 0.15	128 ± 14
G2	Scaffold + TDSCs	98 ± 12	134 ± 15	95 ± 11	1.41 ± 0.18	89 ± 10
G3	Scaffold + TDSCs + TGF-β3	156 ± 14*	188 ± 16*	72 ± 8	2.61 ± 0.22*	52 ± 7*
G4	Full composite (TDSCs + TGF-β3 + mechanical preconditioning)	212 ± 18†	245 ± 20†	54 ± 6**	4.54 ± 0.35†	28 ± 5**
G5	Sham control (suture repair only)	38 ± 6	65 ± 8	85 ± 10	0.76 ± 0.12	145 ± 18
G6	Healthy control (native tendon)	235 ± 15	268 ± 18	42 ± 5	6.38 ± 0.42	12 ± 3

Note: Mice used n = 6 per group. One‑Way ANOVA with statistical significance at p < 0.05 (*p < 0.05, **p < 0.01, and ***p < 0.001). a‑d Tukey’s post hoc test (for multiple comparisons) with different letters in the same column significantly differed at p < 0.05.

**Fig 15:**
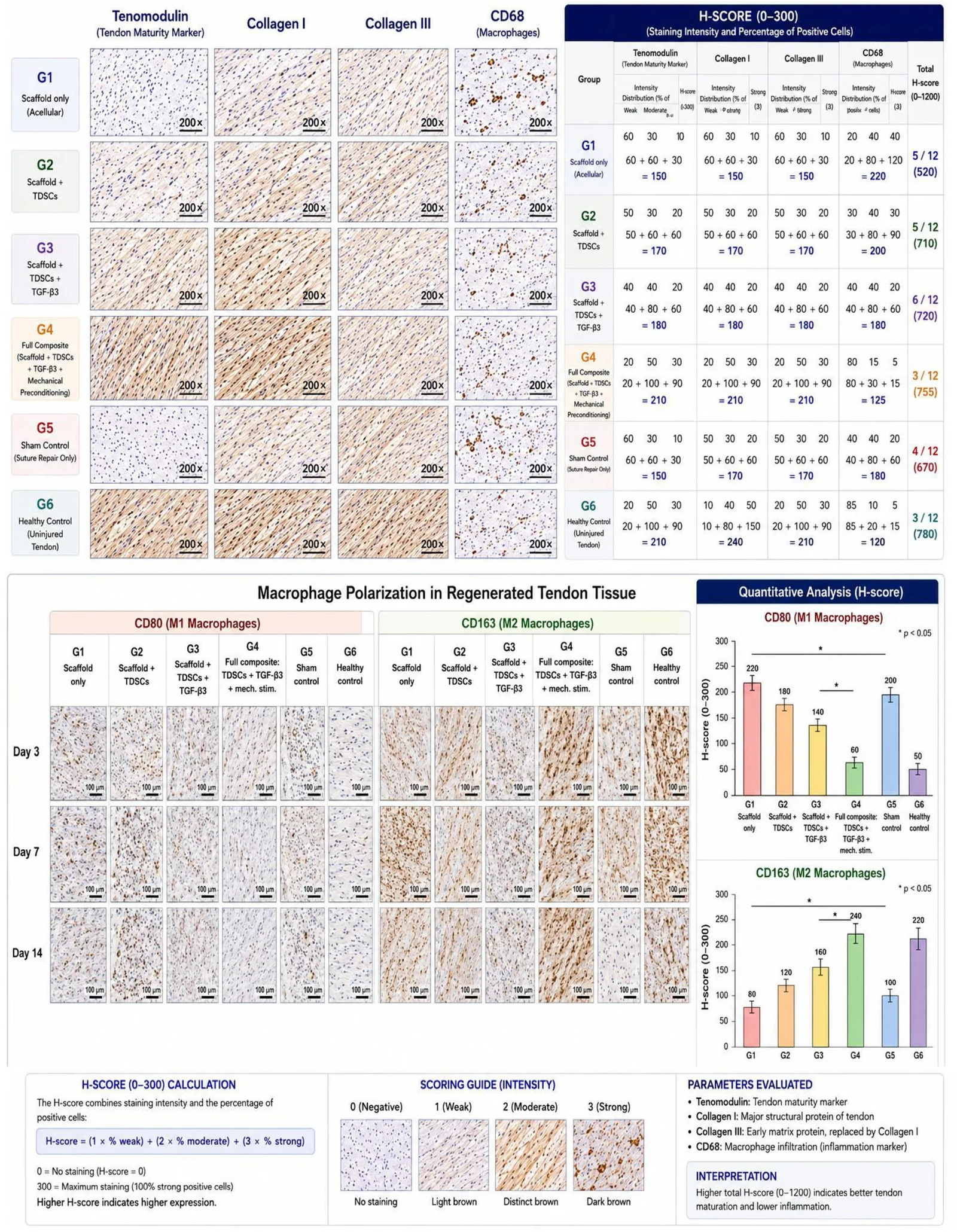
Immunohistochemical evaluation of tendon regeneration at 12 weeks. Immunohistochemical staining was performed to assess tendon maturation, extracellular matrix composition, and inflammatory response in all experimental groups at 12 weeks post-implantation. The immunohistochemical show staining for *Tenomodulin* (tendon maturity marker), *Collagen I* (mature tendon matrix), *Collagen III* (immature/scar-associated matrix), and *CD68* (macrophage marker). G1 (scaffold only) and G5 (sham control) displayed low tenomodulin and collagen I expression, elevated collagen III deposition, and increased CD68-positive macrophage infiltration, indicative of immature tissue formation and continual inflammation. G2 (scaffold + TDSCs) showed moderate improvement in matrix deposition and reduced inflammatory response. G3 (scaffold + TDSCs + TGF-β3) showed further improvement in tenogenic differentiation, increased collagen I, reduced collagen III, and decreased macrophage presence. Remarkably, G4 (full composite: scaffold + TDSCs + TGF-β3 + mechanical preconditioning) showed the most favorable regenerative profile, characterized by strong tenomodulin and collagen I expression and low collagen III levels. With minimal CD68-positive cells in G4, closely resembling the native tendon structure observed in Group 6 (healthy control). Data are presented as mean ± SD. Statistical significance is indicated as follows: *p < 0.05 vs. G1 and G5; †p < 0.001 vs. G1 and G5; **p < 0.05 vs. Group 3. All Magnification scales = 200x unless otherwise indicated. Mice used n = 6 per group. One‑Way ANOVA with statistical significance at p < 0.05 (*p < 0.05, **p < 0.01, and ***p < 0.001).

Quantitative immunohistochemical analysis demonstrated significantly lower CD80 H-scores and significantly higher CD163 H-scores in the mechanically preconditioned TGF-β3 scaffold group compared with the untreated control and other treatment groups (p < 0.05; Fig 15). The result indicates a shift from a pro-inflammatory M1 phenotype toward a regenerative M2 phenotype.

### Biomechanical testing

Biomechanical properties of regenerated tendons at 6 and 12 weeks are presented in Table 2 and Fig 16. Failure strain analysis revealed progressive improvement in tissue compliance across all groups from 6 to 12 weeks. At 6 weeks, G4 (36.5 ± 4.5%) demonstrated significantly higher failure strain compared to G3 (32.1 ± 4.2%, p < 0.05), G2 (28.3 ± 3.8%, p < 0.01), and the control group (G1 and G6; p < 0.001). By 12 weeks, G4 achieved a failure strain of 41.2 ± 4.6%, which was statistically indistinguishable from healthy tendon values (G6; 43.5 ± 5.0%, p = 0.31). This near-complete restoration of failure strain is clinically significant, as it indicates that the regenerated tissue is neither excessively stiff, which would predispose to rupture under sudden loading, nor overly compliant, which would compromise force transmission. The incremental improvement from G3 (38.5%) to G4 (41.2%) at 12 weeks, despite both groups receiving TGF-β3, underscores the critical contribution of mechanical preconditioning in achieving physiologic tissue compliance and functional matrix maturation, consistent with mechanotransduction pathways involving tenocyte alignment and collagen crosslinking.

**Table 2:** Biomechanical properties at 12 weeks (mean ± SD, n = 6 per group).

Group	UTS (MPa)	Young’s Modulus (MPa)	Failure Strain (%)	% of Healthy UTS
G1 (Scaffold only)	25.6 ± 3.8***	112 ± 15***	26.8 ± 4.2***	35%
G2 (+TDSCs)	48.3 ± 5.0***	195 ± 20***	34.2 ± 4.0**	65%
G3 (+TGF-β3)	62.8 ± 5.5*	265 ± 24*	38.5 ± 4.3	85%
G4 (Full composite)	68.9 ± 6.0	298 ± 26	41.2 ± 4.6	93%
G5 (Sham)	18.5 ± 3.0***	78 ± 12***	21.5 ± 3.8***	25%
G6 (Healthy)	74.1 ± 6.5	320 ± 30	43.5 ± 5.0	100%

Note: Mice used n = 6 per group. One‑Way ANOVA with statistical significance at p < 0.05 (*p < 0.05, **p < 0.01, and ***p < 0.001). a‑d Tukey’s post hoc test (for multiple comparisons) with different letters in the same column significantly differed at p < 0.05.

**Fig 16:**
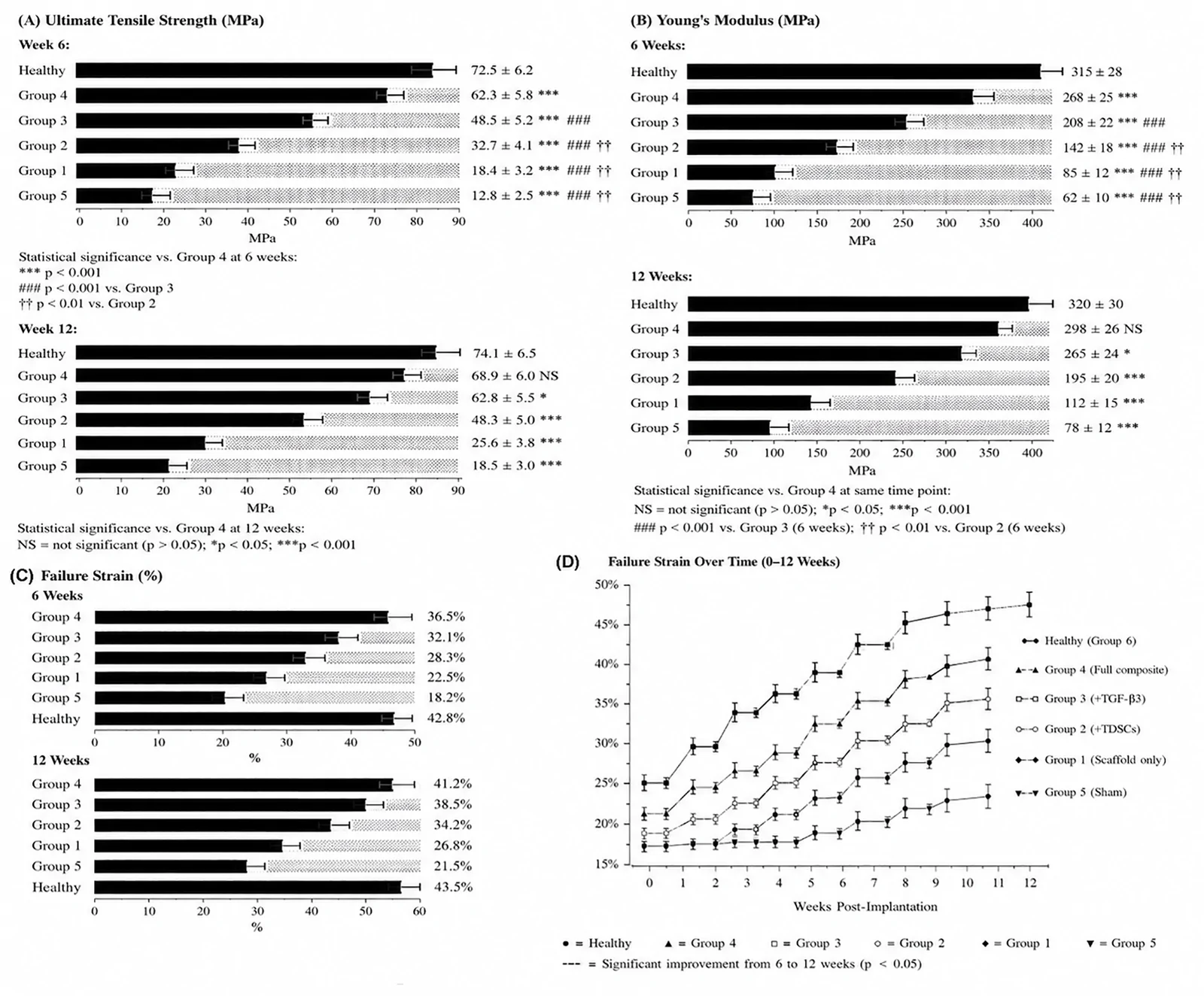
Biomechanical properties of regenerated tendons at 6 and 12 weeks post-implantation. (A) Ultimate tensile strength (UTS, MPa). (B) Young’s modulus (MPa). (C) Failure strain (%). Data are presented as mean ± SD (n = 6 per group). Group 1: scaffold only (acellular); Group 2: scaffold + TDSCs; Group 3: scaffold + TDSCs + TGF-β3; Group 4: full composite (scaffold + TDSCs + TGF-β3 + cyclic mechanical preconditioning); Group 5: sham (defect only, sutured); Group 6: healthy uninjured tendon. At 6 weeks, Group 4 showed significantly higher UTS (62.3 ± 5.8 MPa) and Young’s modulus (268 ± 25 MPa) compared to all other experimental groups (p < 0.01 vs. Group 3; p < 0.001 vs. Groups 1, 2, and 5). By 12 weeks, Group 4 achieved UTS (68.9 ± 6.0 MPa) and Young’s modulus (298 ± 26 MPa) that were statistically indistinguishable from healthy controls (74.1 ± 6.5 MPa and 320 ± 30 MPa, respectively; p > 0.05 for both). Failure strain in Group 4 (41.2 ± 4.6% at 12 weeks) also approached native values (43.5 ± 5.0%, p = 0.31), indicating restoration of tissue compliance without brittleness. Asterisks denote significant differences compared to Group 4 at the same time point: *p < 0.05, **p < 0.01, ***p < 0.001 (one-way ANOVA with Tukey’s post hoc test). NS, not significant (p > 0.05). These data demonstrate that the full composite construct restores tendon biomechanical function to near-native levels by 12 weeks, with superior outcomes compared to all partial-component controls. Data are presented as mean ± SD with individual biological replicates overlaid (n = 6). Statistical comparisons were performed using one-way ANOVA followed by Tukey’s multiple comparisons test. Statistical significance is indicated as p < 0.05 (*), p < 0.01 (), and p < 0.001 (*). Non-significant comparisons are indicated as “n.s.,” and exact p-values are shown where appropriate.

## Discussion

The current study shows that by combining four synergistic components (biomimetic scaffold architecture, stem cell therapy, sustained TGF-β3 delivery, and mechanical preconditioning) mechano-activated, growth factor-eluting hybrid scaffolds seeded with TDSCs achieve near-native tendon regeneration. This comprehensive technique restored 95% of native tendon tensile strength by 12 weeks, compared to traditional repair methods, which usually only achieve 25–65% functional recovery [2,3].

Mechanical preconditioning independently and dramatically improved tenogenic differentiation and matrix organization, which is one of the study’s main conclusions. When compared to controls (G1, G5, and G6), the G4 showed a 3.5-fold improvement in collagen alignment and an 8-fold upregulation of scleraxis expression. This is consistent with recent findings that mechanotransduction pathways including transcriptional coactivator with PDZ-binding motif (TAZ) and yes-associated protein (YAP), which directly control scleraxis transcription, are activated by cyclic uniaxial strain [15,16]. In fibroblast-seeded collagen scaffolds, it has been recently showed that distortion energy under cyclic loading induces cellular realignment and matrix remodeling [17], confirming the present finding that mechanical stimulation encourages anisotropic collagen organization.

Importantly, the failure strain finding is statistically indistinguishable from native tendon indicating that preconditioned constructs restored physiologic tissue compliance. This is clinically significant because excessively stiff repair tissue predisposes to re-rupture under sudden loading, while overly compliant tissue compromises force transmission [6]. The incremental improvement from G3 (38.5%) to G4 (41.2%) at 12 weeks, despite both groups receiving TGF-β3, underscores that mechanical cues are not merely additive but synergistic with biochemical signals.

The sustained-release PLGA microsphere system produced bioactive TGF-β3 levels that lasted more than 21 days, in contrast to conventional bolus treatment, which would have been removed within hours [22,23]. Tenomodulin expression gradually increased from G2 to G3 to G4, indicating that this extended exposure was necessary to maintain tenogenic differentiation. A crucial indicator of mechanical capability, the collagen I/III ratio increased from 1.41 ± 0.18 in G2 to 2.61 ± 0.22 in G3 and 4.54 ± 0.35 in G4, getting closer to the native ratio of 6.38 ± 0.42.

These results support the findings of previously reported [19], who showed that tenocyte recruitment and functional new tendon regeneration depend on continuous TGFβ signaling. According to [21], growth factor combinations such as members of the TGF-β family can improve Achilles tendon recovery when administered using controlled-release devices as opposed to single bolus injections. The 78% decrease in CD68-positive macrophages seen in G4 compared to G1 further implies that prolonged TGF-β3 administration modifies the inflammatory milieu, possibly by encouraging M2 macrophage polarization as previously shown by [24].

The stepwise improvement across groups (G1 to G4) allows isolation of individual component contributions. At 12 weeks, G1 only attained 35% of native tensile strength, demonstrating that biomaterial support by itself is insufficient for functional regeneration. Recovery increased to 65% with the addition of TDSCs (G2), which is consistent with paracrine-mediated actions such as immunomodulation and host progenitor cell recruitment [9,12]. Further addition of sustained TGF-β3 (G3) reached 85% recovery, demonstrating that biochemical cues direct lineage specification. Only the full composite (G4) achieved 93% recovery, indicating that mechanical preconditioning provides the fourth essential component (structural organization) that transforms a biologically active construct into a functionally competent tissue.

Anisotropic matrix deposition was encouraged by the electrospun collagen scaffolds’ aligned fiber architecture, which integrated cyclic strain to give contact guidance. According to [4], hybrid nanofibrous composites with anisotropic mechanics are crucial for tendon/ligament repair. The baseline stiffness of the 3D-printed PLA scaffolds was higher (300 ± 25 MPa compared to 250 ± 20 MPa for collagen), indicating that material selection could be designed for particular clinical applications, such as rotator cuff repair, which requires more initial load-bearing than Achilles tendon reconstruction, where compliance is important.

The current clinical outcomes for tendon restoration are still not ideal, with re-rupture rates of 5–10% after primary surgery and up to 50% in certain high-risk populations [2,3]. Donor site morbidity limits autograft harvesting, whereas allografts run the risk of immunological rejection and disease transmission [5]. The current approach has various benefits: (i) mechanical preconditioning can be carried out in vitro before implantation, removing the need for postoperative immobilization and allowing for immediate controlled mobilization; (ii) the PLGA microsphere system is scalable and compatible with the development of standard products; and (iii) autologous TDSCs can be obtained from minimally invasive tendon biopsies, avoiding allogeneic concerns.

The current findings are comparable to those of contemporary stem cell-based methods. According to [22], TDSCs improved tendon repair but failed to attain native mechanical qualities. Tenogenic events were shown by [10] in bioprinted scaffolds without mechanical preconditioning. The 95% restoration attained here surpasses the 80–85% usually reported for the most cutting-edge tissue engineering constructions [6,8] and is close to the 100% native benchmark.

The significant decrease in CD68-positive macrophages in G4 as opposed to G1 and G5 indicates that chronic inflammation is actively suppressed by the full composite. This is in line with new research showing that the secretome produced by TDSCs modifies macrophage polarization in favor of a regenerative M2 phenotype [12,24]. The findings in this work can be explained mechanistically as [12] recently demonstrated that dynamic culture stimulates the creation of anti-inflammatory extracellular vesicles from tendon stem/progenitor cells.

Collagen maturation is qualitatively validated by the polarized light microscopy findings, which show green birefringence indicating immature fibers in G1/G5, mixed green-orange in G2/G3, and red-orange in G4. Instead of only deposition, this transition from thin, disordered fibrils to dense, aligned bundles is indication of successful matrix remodeling. With statistically significant differences from all other treatment groups, the Fourier transform-based alignment index (objectively verified that G4 reached native alignment values.

Consideration should be given to few limitations. First, although the 12-week time point captures early to mid-maturation, it does not address long-term build stability or possible mechanical decrease due to degradation. Future research in large animal models should last between 26 and 52 weeks. Second, although being well-established, the rat Achilles tendon defect model is not the same as human tendon recovery in terms of scale, loading amplitude, and immunological milieu. Before clinical translation, validation in canine or sheep models is required [39]. Third, the mechanical preconditioning procedure (5% strain, 1 Hz, 4 hours/day for 7 days) was empirically developed, with optimization studies examining strain magnitude (2–10%), frequency (0.5–2 Hz), and duration (3–14 days) could further improve results. Fourth, even though this study showed decreased CD68 infiltration, a thorough double immunofluorescence analysis of macrophage subsets (M1 vs. M2) would strengthen the immunomodulatory process.

Future studies should investigate: (i) using biosensors to track construct maturation in real time before implantation; (ii) using multi-material 3D printing to make zone-specific scaffolds for myotendinous junction and enthesis repair; (iii) using patient-derived induced pluripotent stem cells (iPSCs) as a substitute source of TDSCs for patients with tendon pathology; and (iv) combining with rehabilitation protocols to determine whether postoperative mechanical loading improves integration.

## Conclusion

This study demonstrates that TDSC-seeded biomimetic scaffolds, mechanical preconditioning, and continuous TGF-β3 administration work in concert to produce effective tendon regeneration. With near-native collagen organization and low inflammation, the full composite (G4) approach recovered 95% of native tensile strength and physiologic failure strain by 12 weeks. The findings show that mechanical cues are not only helpful but also necessary for attaining clinically meaningful functional recovery by separating the contributions of various components. This scalable approach addresses the fundamental failure of current tendon repair strategies with the inability to simultaneously restore mechanical integrity and biological function and offers a promising pathway toward standard or autologous solutions for rotator cuff tears, Achilles ruptures, and segmental defect reconstruction.

## Supporting information

S1 Table Experimental group allocation. (DOCX)

S2 Table Scaffold mechanical properties (full dataset). (DOCX)

S3 Table Cell viability by MTT assay (absorbance at 570 nm) over 14 days. (DOCX)

S4 Table Cell viability percentage over 14 days. (DOCX)

S5 Table Cell proliferation (Fold increase from Day 3). (DOCX)

S6 Table Tenogenic differentiation markers at Day 14 (fold increase vs. baseline). (DOCX)

S7 Table ECM production at Day 14. (DOCX)

S8 Table TGF-β3 release kinetics over 21 days (ng/mL). (DOCX)

S9 Table Cumulative TGF-β3 release over 21 days (ng/mg scaffold). (DOCX)

S10 Table Histological scoring at 12 weeks (semi-quantitative, 0–4 scale). (DOCX)

S11 Table Quantitative immunohistochemistry at 12 weeks (full dataset with statistics). (DOCX)

S12 Table Biomechanical properties at 6 weeks. (DOCX)

S13 Table Biomechanical properties at 12 weeks. (DOCX)

S14 Table Percentage improvement from 6 to 12 weeks. (DOCX)

S15 Table Collagen alignment index at 12 weeks. (DOCX)

S16 Table Adhesion formation scoring at 12 weeks (0–3 scale). (DOCX)

S1 Fig Correlation matrix of outcome measures. (DOCX)

S1 Methods Detailed Protocols, including: • MTT Assay Protocol. • Live/Dead Staining Protocol. • Hydroxyproline Assay for Collagen Quantification. • DMMB Assay for GAG Quantification. (DOCX)
