# Supplementary material for: Development of mechanically optimized biomimetic hybrid scaffold–stem cell constructs for functional tendon regeneration and restoration of mechanical integrity
Source: PLoS One. 2026 Sep 28;21(9):e0359084. doi: 10.1371/journal.pone.0359084 (PMC13618886; doi:10.1371/journal.pone.0359084)
Supplement: Supplementary file 1 [file pone.0359084.s001.docx]

**S1 Table**

| Group | Code | Description | n (6 wk) | n (12 wk) | Total |
| --- | --- | --- | --- | --- | --- |
| 1 | G1 | Scaffold only (acellular) | 6 | 6 | 12 |
| 2 | G2 | Scaffold + TDSCs | 6 | 6 | 12 |
| 3 | G3 | Scaffold + TDSCs + TGF-β3 | 6 | 6 | 12 |
| 4 | G4 | Full composite (TDSCs + TGF-β3 + mechanical preconditioning) | 6 | 6 | 12 |
| 5 | G5 | Sham control (suture repair only) | 6 | 6 | 12 |
| 6 | G6 | Healthy control (uninjured) | 6 | 6 | 12 |
| **Total** |  |  | **36** | **36** | **72** |
