# Supplementary material for: Development of mechanically optimized biomimetic hybrid scaffold–stem cell constructs for functional tendon regeneration and restoration of mechanical integrity
Source: PLoS One. 2026 Sep 28;21(9):e0359084. doi: 10.1371/journal.pone.0359084 (PMC13618886; doi:10.1371/journal.pone.0359084)
Supplement: Supplementary file 2 [file pone.0359084.s002.docx]

**S2 Table**

| Scaffold Type | Condition | Tensile Strength (MPa) | Elastic Modulus (MPa) | Pore Size (μm) | Fiber Alignment |
| --- | --- | --- | --- | --- | --- |
| Electrospun Collagen | Static | 80 ± 10 | 250 ± 20 | 50-100 | Aligned |
| Electrospun Collagen | Preconditioned (7 days) | 85 ± 8 | 245 ± 18 | 50-100 | Highly aligned |
| 3D-printed PLA | Static | 90 ± 15 | 300 ± 25 | 75-120 | Random |
| 3D-printed PLA | Preconditioned (7 days) | 85 ± 12 | 245 ± 22 | 75-120 | Partially aligned |

*Note: Preconditioning = 5% cyclic strain, 1 Hz, 4 hours/day for 7 days*
