# Supplementary material for: Development of mechanically optimized biomimetic hybrid scaffold–stem cell constructs for functional tendon regeneration and restoration of mechanical integrity
Source: PLoS One. 2026 Sep 28;21(9):e0359084. doi: 10.1371/journal.pone.0359084 (PMC13618886; doi:10.1371/journal.pone.0359084)
Supplement: Supplementary file 3 [file pone.0359084.s003.docx]

**S3 Table**

| Group | Day 1 | Day 3 | Day 5 | Day 7 | Day 10 | Day 14 |
| --- | --- | --- | --- | --- | --- | --- |
| G1 (Scaffold only) | 0.18 ± 0.03 | 0.22 ± 0.04 | 0.25 ± 0.03 | 0.28 ± 0.04 | 0.30 ± 0.03 | 0.32 ± 0.04 |
| G2 (Scaffold + TDSCs) | 0.35 ± 0.04 | 0.48 ± 0.05 | 0.62 ± 0.06 | 0.78 ± 0.07 | 0.89 ± 0.08 | 1.02 ± 0.09 |
| G3 (+ TGF-β3) | 0.38 ± 0.04 | 0.52 ± 0.05 | 0.68 ± 0.06 | 0.85 ± 0.07 | 0.98 ± 0.08 | 1.15 ± 0.10 |
| G4 (Full composite) | 0.42 ± 0.04 | 0.58 ± 0.05 | 0.75 ± 0.06 | 0.95 ± 0.08 | 1.12 ± 0.09 | 1.32 ± 0.11 |
| G5 (Sham) | 0.15 ± 0.02 | 0.18 ± 0.03 | 0.20 ± 0.03 | 0.22 ± 0.03 | 0.23 ± 0.03 | 0.25 ± 0.03 |
| G6 (Healthy) | 0.45 ± 0.04 | 0.60 ± 0.05 | 0.78 ± 0.06 | 0.98 ± 0.08 | 1.18 ± 0.09 | 1.38 ± 0.10 |

*Data presented as mean ± SD (n=5-6 per group)*
