# Supplementary material for: Development of mechanically optimized biomimetic hybrid scaffold–stem cell constructs for functional tendon regeneration and restoration of mechanical integrity
Source: PLoS One. 2026 Sep 28;21(9):e0359084. doi: 10.1371/journal.pone.0359084 (PMC13618886; doi:10.1371/journal.pone.0359084)
Supplement: Supplementary file 4 [file pone.0359084.s004.docx]

**S4 Table**

| Group | Day 3 | Day 7 | Day 14 |
| --- | --- | --- | --- |
| G1 (Scaffold only) | 62 ± 5% | 68 ± 6% | 72 ± 5% |
| G2 (Scaffold + TDSCs) | 78 ± 4% | 85 ± 5% | 89 ± 4% |
| G3 (+ TGF-β3) | 82 ± 4% | 88 ± 4% | 92 ± 4% |
| G4 (Full composite) | 86 ± 3% | 92 ± 3% | 96 ± 3% |
| G5 (Sham) | 58 ± 6% | 62 ± 5% | 65 ± 5% |
| G6 (Healthy) | 88 ± 3% | 94 ± 3% | 97 ± 2% |
