# Supplementary material for: Development of mechanically optimized biomimetic hybrid scaffold–stem cell constructs for functional tendon regeneration and restoration of mechanical integrity
Source: PLoS One. 2026 Sep 28;21(9):e0359084. doi: 10.1371/journal.pone.0359084 (PMC13618886; doi:10.1371/journal.pone.0359084)
Supplement: Supplementary file 5 [file pone.0359084.s005.docx]

**S5 Table**

| Group | Day 3 | Day 7 | Day 14 |
| --- | --- | --- | --- |
| G1 (Scaffold only) | 1.2 ± 0.1 | 1.5 ± 0.2 | 1.8 ± 0.2 |
| G2 (Scaffold + TDSCs) | 1.4 ± 0.1 | 2.2 ± 0.2 | 2.9 ± 0.3 |
| G3 (+ TGF-β3) | 1.4 ± 0.1 | 2.4 ± 0.2 | 3.3 ± 0.3 |
| G4 (Full composite) | 1.5 ± 0.1 | 2.7 ± 0.2 | 3.8 ± 0.3 |
| G5 (Sham) | 1.1 ± 0.1 | 1.3 ± 0.1 | 1.5 ± 0.2 |
| G6 (Healthy) | 1.5 ± 0.1 | 2.8 ± 0.2 | 3.9 ± 0.3 |
