# Supplementary material for: Development of mechanically optimized biomimetic hybrid scaffold–stem cell constructs for functional tendon regeneration and restoration of mechanical integrity
Source: PLoS One. 2026 Sep 28;21(9):e0359084. doi: 10.1371/journal.pone.0359084 (PMC13618886; doi:10.1371/journal.pone.0359084)
Supplement: Supplementary file 6 [file pone.0359084.s006.docx]

**S6 Table**

| Group | Scleraxis | Tenomodulin |
| --- | --- | --- |
| G1 (Scaffold only) | 1.0 ± 0.1 | 1.0 ± 0.1 |
| G2 (Scaffold + TDSCs) | 2.5 ± 0.3 | 2.1 ± 0.2 |
| G3 (+ TGF-β3) | 4.2 ± 0.4 | 3.5 ± 0.3 |
| G4 (Full composite) | 8.0 ± 0.6 | 5.8 ± 0.5 |
| G5 (Sham) | 0.9 ± 0.1 | 0.9 ± 0.1 |
| G6 (Healthy) | 8.5 ± 0.6 | 6.2 ± 0.5 |
