# Supplementary material for: Development of mechanically optimized biomimetic hybrid scaffold–stem cell constructs for functional tendon regeneration and restoration of mechanical integrity
Source: PLoS One. 2026 Sep 28;21(9):e0359084. doi: 10.1371/journal.pone.0359084 (PMC13618886; doi:10.1371/journal.pone.0359084)
Supplement: Supplementary file 7 [file pone.0359084.s007.docx]

**S7 Table**

| Group | Collagen Type I (μg/mg scaffold) | GAG Content (μg/mg scaffold) | Collagen I/III Ratio |
| --- | --- | --- | --- |
| G1 (Scaffold only) | 12 ± 3 | 8 ± 2 | 0.9 ± 0.1 |
| G2 (Scaffold + TDSCs) | 28 ± 4 | 16 ± 3 | 1.3 ± 0.2 |
| G3 (+ TGF-β3) | 42 ± 5 | 22 ± 3 | 2.2 ± 0.2 |
| G4 (Full composite) | 60 ± 6 | 30 ± 3 | 3.8 ± 0.3 |
| G5 (Sham) | 10 ± 2 | 6 ± 2 | 0.7 ± 0.1 |
| G6 (Healthy) | 65 ± 5 | 32 ± 3 | 4.2 ± 0.3 |
