# Supplementary material for: Development of mechanically optimized biomimetic hybrid scaffold–stem cell constructs for functional tendon regeneration and restoration of mechanical integrity
Source: PLoS One. 2026 Sep 28;21(9):e0359084. doi: 10.1371/journal.pone.0359084 (PMC13618886; doi:10.1371/journal.pone.0359084)
Supplement: Supplementary file 8 [file pone.0359084.s008.docx]

**S8 Table**

| Group | Day 1 | Day 3 | Day 7 | Day 14 | Day 21 |
| --- | --- | --- | --- | --- | --- |
| G1 (Scaffold only) | 0.5 ± 0.2 | 0.4 ± 0.2 | 0.4 ± 0.1 | 0.3 ± 0.1 | 0.3 ± 0.1 |
| G2 (Scaffold + TDSCs) | 1.2 ± 0.3 | 1.5 ± 0.3 | 1.8 ± 0.4 | 2.0 ± 0.4 | 1.8 ± 0.3 |
| G3 (+ TGF-β3) | 8.5 ± 1.2 | 12.4 ± 1.5 | 15.8 ± 1.8 | 14.2 ± 1.6 | 10.5 ± 1.4 |
| G4 (Full composite) | 10.2 ± 1.4 | 15.6 ± 1.6 | 20.5 ± 2.0 | 22.8 ± 2.2 | 18.5 ± 1.8 |
| G5 (Sham) | 0.3 ± 0.1 | 0.3 ± 0.1 | 0.2 ± 0.1 | 0.2 ± 0.1 | 0.2 ± 0.1 |
| G6 (Healthy) | 0.2 ± 0.1 | 0.2 ± 0.1 | 0.2 ± 0.1 | 0.2 ± 0.1 | 0.2 ± 0.1 |
