# Supplementary material for: Development of mechanically optimized biomimetic hybrid scaffold–stem cell constructs for functional tendon regeneration and restoration of mechanical integrity
Source: PLoS One. 2026 Sep 28;21(9):e0359084. doi: 10.1371/journal.pone.0359084 (PMC13618886; doi:10.1371/journal.pone.0359084)
Supplement: Supplementary file 10 [file pone.0359084.s010.docx]

**S10 Table**

| Group | Collagen Organization | Cellularity | Vascularity | Inflammatory Infiltration | Total Score |
| --- | --- | --- | --- | --- | --- |
| G1 (Scaffold only) | 1.2 ± 0.3 | 2.5 ± 0.4 | 2.0 ± 0.3 | 2.8 ± 0.4 | 8.5 ± 1.0 |
| G2 (Scaffold + TDSCs) | 2.0 ± 0.3 | 2.2 ± 0.3 | 1.5 ± 0.3 | 2.0 ± 0.3 | 7.7 ± 0.9 |
| G3 (+ TGF-β3) | 2.8 ± 0.3 | 1.8 ± 0.3 | 1.2 ± 0.2 | 1.2 ± 0.2 | 7.0 ± 0.8 |
| G4 (Full composite) | 3.5 ± 0.3 | 1.2 ± 0.2 | 0.8 ± 0.2 | 0.5 ± 0.2 | 6.0 ± 0.6 |
| G5 (Sham) | 1.0 ± 0.2 | 2.8 ± 0.4 | 2.2 ± 0.3 | 3.0 ± 0.4 | 9.0 ± 1.0 |
| G6 (Healthy) | 4.0 ± 0.0 | 1.0 ± 0.1 | 0.5 ± 0.1 | 0.2 ± 0.1 | 5.7 ± 0.3 |

*Scoring: 0=normal, 1=mild, 2=moderate, 3=severe, 4=very severe*
