# Supplementary material for: Development of mechanically optimized biomimetic hybrid scaffold–stem cell constructs for functional tendon regeneration and restoration of mechanical integrity
Source: PLoS One. 2026 Sep 28;21(9):e0359084. doi: 10.1371/journal.pone.0359084 (PMC13618886; doi:10.1371/journal.pone.0359084)
Supplement: Supplementary file 11 [file pone.0359084.s011.docx]

**S11 Table**

| Group | Tenomodulin (IHC score) | Collagen I (IHC score) | Collagen III (IHC score) | Collagen I/III Ratio | CD68+ Cells (per HPF) |
| --- | --- | --- | --- | --- | --- |
| G1 | 45 ± 8 | 82 ± 10 | 78 ± 9 | 1.05 ± 0.15 | 128 ± 14 |
| G2 | 98 ± 12 | 134 ± 15 | 95 ± 11 | 1.41 ± 0.18 | 89 ± 10 |
| G3 | 156 ± 14* | 188 ± 16* | 72 ± 8 | 2.61 ± 0.22* | 52 ± 7* |
| G4 | 212 ± 18† | 245 ± 20† | 54 ± 6** | 4.54 ± 0.35† | 28 ± 5** |
| G5 | 38 ± 6 | 65 ± 8 | 85 ± 10 | 0.76 ± 0.12 | 145 ± 18 |
| G6 | 235 ± 15 | 268 ± 18 | 42 ± 5 | 6.38 ± 0.42 | 12 ± 3 |

*Statistical significance: *p < 0.05 vs. G1 and G5; †p < 0.001 vs. G1 and G5; **p < 0.05 vs. G3; HPF = high-power field (400×)*
