# Supplementary material for: Development of mechanically optimized biomimetic hybrid scaffold–stem cell constructs for functional tendon regeneration and restoration of mechanical integrity
Source: PLoS One. 2026 Sep 28;21(9):e0359084. doi: 10.1371/journal.pone.0359084 (PMC13618886; doi:10.1371/journal.pone.0359084)
Supplement: Supplementary file 12 [file pone.0359084.s012.docx]

**S12 Table**

| Group | Ultimate Tensile Strength (MPa) | Young's Modulus (MPa) | Failure Strain (%) | % of Healthy UTS |
| --- | --- | --- | --- | --- |
| G1 (Scaffold only) | 18.5 ± 3.2*** | 85 ± 12*** | 20.5 ± 3.5*** | 25% |
| G2 (Scaffold + TDSCs) | 35.6 ± 4.5*** | 145 ± 18*** | 28.3 ± 3.8** | 48% |
| G3 (+ TGF-β3) | 48.2 ± 5.0** | 210 ± 22** | 32.1 ± 4.2* | 65% |
| G4 (Full composite) | 62.3 ± 5.8 | 268 ± 25 | 36.5 ± 4.5 | 84% |
| G5 (Sham) | 12.5 ± 2.8*** | 58 ± 10*** | 16.5 ± 3.0*** | 17% |
| G6 (Healthy) | 74.1 ± 6.5 | 320 ± 30 | 43.5 ± 5.0 | 100% |

*Statistical significance vs. G4: ***p < 0.001; **p < 0.01; *p < 0.05*
