# Supplementary material for: Development of mechanically optimized biomimetic hybrid scaffold–stem cell constructs for functional tendon regeneration and restoration of mechanical integrity
Source: PLoS One. 2026 Sep 28;21(9):e0359084. doi: 10.1371/journal.pone.0359084 (PMC13618886; doi:10.1371/journal.pone.0359084)
Supplement: Supplementary file 13 [file pone.0359084.s013.docx]

**S13 Table**

| Group | Ultimate Tensile Strength (MPa) | Young's Modulus (MPa) | Failure Strain (%) | % of Healthy UTS |
| --- | --- | --- | --- | --- |
| G1 (Scaffold only) | 25.6 ± 3.8*** | 112 ± 15*** | 26.8 ± 4.2*** | 35% |
| G2 (Scaffold + TDSCs) | 48.3 ± 5.0*** | 195 ± 20*** | 34.2 ± 4.0** | 65% |
| G3 (+ TGF-β3) | 62.8 ± 5.5* | 265 ± 24* | 38.5 ± 4.3 | 85% |
| G4 (Full composite) | 68.9 ± 6.0 | 298 ± 26 | 41.2 ± 4.6 | 93% |
| G5 (Sham) | 18.5 ± 3.0*** | 78 ± 12*** | 21.5 ± 3.8*** | 25% |
| G6 (Healthy) | 74.1 ± 6.5 | 320 ± 30 | 43.5 ± 5.0 | 100% |

*Statistical significance vs. G4: ***p < 0.001; **p < 0.01; *p < 0.05; ns = not significant (p > 0.05)*
