# Supplementary material for: Development of mechanically optimized biomimetic hybrid scaffold–stem cell constructs for functional tendon regeneration and restoration of mechanical integrity
Source: PLoS One. 2026 Sep 28;21(9):e0359084. doi: 10.1371/journal.pone.0359084 (PMC13618886; doi:10.1371/journal.pone.0359084)
Supplement: Supplementary file 14 [file pone.0359084.s014.docx]

**S14 Table**

| Group | UTS Improvement (%) | Young's Modulus Improvement (%) | Failure Strain Improvement (%) |
| --- | --- | --- | --- |
| G1 | +38% | +32% | +31% |
| G2 | +36% | +34% | +21% |
| G3 | +30% | +26% | +20% |
| G4 | +11% | +11% | +13% |
| G5 | +48% | +34% | +30% |
| G6 (Healthy) | 0% | 0% | 0% |
