# Supplementary material for: Development of mechanically optimized biomimetic hybrid scaffold–stem cell constructs for functional tendon regeneration and restoration of mechanical integrity
Source: PLoS One. 2026 Sep 28;21(9):e0359084. doi: 10.1371/journal.pone.0359084 (PMC13618886; doi:10.1371/journal.pone.0359084)
Supplement: Supplementary file 15 [file pone.0359084.s015.docx]

**S15 Table**

| Group | Alignment Index (0-1 scale) | % of Healthy |
| --- | --- | --- |
| G1 (Scaffold only) | 0.35 ± 0.08 | 38% |
| G2 (Scaffold + TDSCs) | 0.52 ± 0.07 | 57% |
| G3 (+ TGF-β3) | 0.68 ± 0.06 | 74% |
| G4 (Full composite) | 0.85 ± 0.05 | 92% |
| G5 (Sham) | 0.28 ± 0.07 | 30% |
| G6 (Healthy) | 0.92 ± 0.04 | 100% |
