# Supplementary material for: Development of mechanically optimized biomimetic hybrid scaffold–stem cell constructs for functional tendon regeneration and restoration of mechanical integrity
Source: PLoS One. 2026 Sep 28;21(9):e0359084. doi: 10.1371/journal.pone.0359084 (PMC13618886; doi:10.1371/journal.pone.0359084)
Supplement: Supplementary file 16 [file pone.0359084.s016.docx]

**S16 Table**

| Group | Peritendinous Adhesion | Intratendinous Adhesion | Total Adhesion Score |
| --- | --- | --- | --- |
| G1 | 2.2 ± 0.4 | 1.8 ± 0.4 | 4.0 ± 0.6 |
| G2 | 1.5 ± 0.3 | 1.2 ± 0.3 | 2.7 ± 0.5 |
| G3 | 1.0 ± 0.2 | 0.8 ± 0.2 | 1.8 ± 0.4 |
| G4 | 0.5 ± 0.2 | 0.3 ± 0.1 | 0.8 ± 0.3 |
| G5 | 2.5 ± 0.4 | 2.0 ± 0.4 | 4.5 ± 0.6 |
| G6 | 0.0 ± 0.0 | 0.0 ± 0.0 | 0.0 ± 0.0 |

*Scoring: 0=none, 1=mild, 2=moderate, 3=severe*
