# Supplementary material for: Development of mechanically optimized biomimetic hybrid scaffold–stem cell constructs for functional tendon regeneration and restoration of mechanical integrity
Source: PLoS One. 2026 Sep 28;21(9):e0359084. doi: 10.1371/journal.pone.0359084 (PMC13618886; doi:10.1371/journal.pone.0359084)
Supplement: Supplementary file 17 [file pone.0359084.s017.docx]

**S1 Fig**

| Parameter | UTS | Collagen I/III | Alignment | Tenomodulin | CD68 | Adhesion |
| --- | --- | --- | --- | --- | --- | --- |
| UTS | 1.00 | 0.92 | 0.88 | 0.91 | -0.85 | -0.89 |
| Collagen I/III | 0.92 | 1.00 | 0.94 | 0.95 | -0.90 | -0.91 |
| Alignment | 0.88 | 0.94 | 1.00 | 0.89 | -0.87 | -0.88 |
| Tenomodulin | 0.91 | 0.95 | 0.89 | 1.00 | -0.92 | -0.90 |
| CD68 | -0.85 | -0.90 | -0.87 | -0.92 | 1.00 | 0.88 |
| Adhesion | -0.89 | -0.91 | -0.88 | -0.90 | 0.88 | 1.00 |

*Strong positive correlations (≥0.85) between mechanical and histological parameters; strong negative correlations between inflammation markers and functional outcomes.*
