# Supplementary material for: Development of mechanically optimized biomimetic hybrid scaffold–stem cell constructs for functional tendon regeneration and restoration of mechanical integrity
Source: PLoS One. 2026 Sep 28;21(9):e0359084. doi: 10.1371/journal.pone.0359084 (PMC13618886; doi:10.1371/journal.pone.0359084)
Supplement: Supplementary file 18 [file pone.0359084.s018.docx]

**S1 Methods**

**Method 1. MTT Assay Protocol**

- Scaffold constructs washed with PBS
- Incubated with 500 μL of 0.5 mg/mL MTT solution (Sigma-Aldrich) in culture medium
- Incubation at 37°C for 4 hours
- Formazan crystals dissolved in 500 μL DMSO
- Absorbance measured at 570 nm using microplate reader (BioTek Synergy H1)
- Background subtraction at 690 nm

**Method 2. Live/Dead Staining Protocol**

- Staining solution: 2 μM Calcein-AM + 4 μM Ethidium homodimer-1 in PBS
- Incubation at room temperature for 30 minutes (protected from light)
- Imaging using Zeiss LSM 980 confocal microscope
- Excitation/emission: 488/515 nm (live), 530/635 nm (dead)
- Minimum 5 fields per scaffold analyzed using ImageJ

**Method 3. Hydroxyproline Assay for Collagen Quantification**

- Scaffold hydrolyzed in 6N HCl at 110°C for 18 hours
- Dried and reconstituted in dH₂O
- Chloramine-T oxidation (20 min at RT)
- Ehrlich's reagent incubation (15 min at 60°C)
- Absorbance at 560 nm
- Collagen content = hydroxyproline × 7.69

**Method 4. DMMB Assay for GAG Quantification**

- Papain digestion (125 μg/mL in phosphate buffer, pH 6.5, 60°C, 16 hours)
- Reaction with DMMB dye (16 mg DMMB in 1 L dH₂O containing 3.04 g glycine, 2.37 g NaCl, 95 mL 0.1 M HCl)
- Absorbance at 525 nm immediately after mixing
- Chondroitin-6-sulfate standard curve (0-50 μg/mL)
