# Supplementary material for: Development of mechanically optimized biomimetic hybrid scaffold–stem cell constructs for functional tendon regeneration and restoration of mechanical integrity
Source: PLoS One. 2026 Sep 28;21(9):e0359084. doi: 10.1371/journal.pone.0359084 (PMC13618886; doi:10.1371/journal.pone.0359084)
Supplement: Supplementary file 19 [file pone.0359084.s020.docx]

**Response to Editor Comments**

When submitting your revision, we need you to address these additional requirements.

**Comments 1:**

1. Please ensure that your manuscript meets PLOS ONE's style requirements, including those for file naming. The PLOS ONE style templates can be found at

<https://journals.plos.org/plosone/s/file?id=wjVg/PLOSOne_formatting_sample_main_body.pdf> and

[https://journals.plos.org/plosone/s/file?id=ba62/PLOSOne_formatting_sample_title_authors_affiliations.pdf](https://track.editorialmanager.com/CL0/https:%2F%2Fjournals.plos.org%2Fplosone%2Fs%2Ffile%3Fid=ba62%2FPLOSOne_formatting_sample_title_authors_affiliations.pdf/1/010f019f3ab04cd9-caa55c55-41d1-4fe1-9fd2-0dc8a599dac3-000000/2ky-6AUggQzRGg5RO66t7L6abnhaZ_esT31cc0u60LI=258)

**Response 1**:

The author thanks the Editor for this important observation. The manuscript has been carefully reviewed and revised to comply with the PLOS ONE formatting and style requirements. The author has updated the manuscript formatting in accordance with the PLOS ONE style templates, including the title page, author affiliations, section headings, figure and table formatting, references, and overall manuscript layout. The author has also ensured that the manuscript file naming and submission files conform to the journal's submission guidelines.

**Comments 2:**

2. Please include captions for your Supporting Information files at the end of your manuscript, and update any in-text citations to match accordingly. Please see our Supporting Information guidelines for more information: <http://journals.plos.org/plosone/s/supporting-information>.

**Response 2**:

The author thanks the Editor for this helpful suggestion. The author has revised the manuscript to comply with the PLOS ONE supporting information guidelines. Captions for all supporting information files have been added at the end of the manuscript, and all in-text citations have been reviewed and updated to ensure consistency with the corresponding supporting information file names and captions.

**Comments 3:**

3. We note that Figures 1, 2 and 3 in your submission contain copyrighted images. All PLOS content is published under the Creative Commons Attribution License (CC BY 4.0), which means that the manuscript, images, and Supporting Information files will be freely available online, and any third party is permitted to access, download, copy, distribute, and use these materials in any way, even commercially, with proper attribution. For more information, see our copyright guidelines: <http://journals.plos.org/plosone/s/licenses-and-copyright>.

We require you to either (1) present written permission from the copyright holder to publish these figures specifically under the CC BY 4.0 license, or (2) remove the figures from your submission:

1.        You may seek permission from the original copyright holder of Figure(s) [#] to publish the content specifically under the CC BY 4.0 license.

We recommend that you contact the original copyright holder with the Content Permission Form (<http://journals.plos.org/plosone/s/file?id=7c09/content-permission-form.pdf>) and the following text:

“I request permission for the open-access journal PLOS ONE to publish XXX under the Creative Commons Attribution License (CCAL) CC BY 4.0 (<http://creativecommons.org/licenses/by/4.0/>). Please be aware that this license allows unrestricted use and distribution, even commercially, by third parties. Please reply and provide explicit written permission to publish XXX under a CC BY license and complete the attached form.”

Please upload the completed Content Permission Form or other proof of granted permissions as an "Other" file with your submission.

In the figure caption of the copyrighted figure, please include the following text: “Reprinted from [ref] under a CC BY license, with permission from [name of publisher], original copyright [original copyright year].”

2.        If you are unable to obtain permission from the original copyright holder to publish these figures under the CC BY 4.0 license or if the copyright holder’s requirements are incompatible with the CC BY 4.0 license, please either i) remove the figure or ii) supply a replacement figure that complies with the CC BY 4.0 license. Please check copyright information on all replacement figures and update the figure caption with source information. If applicable, please specify in the figure caption text when a figure is similar but not identical to the original image and is therefore for illustrative purposes only.

**Response 3**:

The author thanks the Editor for bringing this important copyright issue to my attention The author carefully reviewed Figures 1, 2, and 3 and have replaced (or removed) the previously included third-party visual elements with original figures created entirely by the author. The revised figures were manually prepared using conventional scientific illustration software and do not contain copyrighted material requiring permission from third-party copyright holders. Accordingly, no copyrighted images remain in the revised manuscript, and the figures are suitable for publication under the Creative Commons Attribution (CC BY 4.0) license. The author has also reviewed the corresponding figure legends to ensure that they accurately reflect the origin of the figures and comply with the PLOS ONE copyright and licensing requirements.

**Comments 4:**

4. In the online submission form, you indicated that "All relevant data from this study will be made available upon request."

All PLOS journals now require all data underlying the findings described in their manuscript to be freely available to other researchers, either 1. In a public repository, 2. Within the manuscript itself, or 3. Uploaded as supplementary information.

This policy applies to all data except where public deposition would breach compliance with the protocol approved by your research ethics board. If your data cannot be made publicly available for ethical or legal reasons (e.g., public availability would compromise patient privacy), please explain your reasons on resubmission and your exemption request will be escalated for approval.

**Response 4**:

The author thanks the Editor for highlighting the journal's data availability policy. The author has revised the manuscript and updated the data availability statement to comply with the PLOS ONE requirements. All data underlying the findings of this study are now provided within the manuscript and its supporting information files. The supporting information includes the complete experimental datasets, quantitative measurements, biomechanical data, histological scoring, immunohistochemical analyses, and detailed experimental protocols necessary to support the results and facilitate reproducibility. Accordingly, the previous statement indicating that data would be made available upon request has been removed. The author believes that the revised data availability statement fully complies with the journal's policy on open data.

**Comments 5:**

5. If the reviewer comments include a recommendation to cite specific previously published works, please review and evaluate these publications to determine whether they are relevant and should be cited. There is no requirement to cite these works unless the editor has indicated otherwise.

**Response 5**:

The author thanks the Editor for this guidance. The author carefully reviewed all reviewer comments and confirmed that no specific publications were recommended for citation. Nevertheless, the author has reviewed the relevant literature during the revision process and ensured that the manuscript contains appropriate and up-to-date references supporting the study's methodology, results, and discussion. The author believes that the reference list is current and relevant to the scope of the manuscript.

**Comments 6:**

Additional Editor Comments:

Please revise the MS based on the reviewers' comments and suggestion and resubmit for further consideration

**Response 6**:

The author sincerely thanks the Editor for the careful evaluation of this manuscript and for providing the author with the opportunity to revise and improve this work. In response to the Editor's recommendation and the reviewers' constructive comments, the author comprehensively revised the manuscript to enhance its scientific rigor, methodological transparency, data presentation, and overall clarity. The author has expanded the materials and methods section with additional experimental details to improve reproducibility, clarified the experimental design and rationale, strengthened the quantitative analyses by incorporating validated histological and immunohistochemical scoring methods, improved the presentation and annotation of figures, updated the statistical reporting, revised the discussion to ensure that the conclusions are fully supported by the experimental findings, and addressed all editorial comments regarding formatting, supporting information, copyright compliance, and data availability. Relevant reviewer-suggested revisions have also been incorporated throughout the manuscript where appropriate. The author believes that these revisions have substantially strengthened the quality of the manuscript and has addressed all comments raised by the Editor and reviewers. A detailed point-by-point response to each comment is provided in this rebuttal letter. The author respectfully submits the revised manuscript for further consideration and appreciate the Editor's and reviewers' time and valuable feedback.
