# Supplementary material for: Development of mechanically optimized biomimetic hybrid scaffold–stem cell constructs for functional tendon regeneration and restoration of mechanical integrity
Source: PLoS One. 2026 Sep 28;21(9):e0359084. doi: 10.1371/journal.pone.0359084 (PMC13618886; doi:10.1371/journal.pone.0359084)
Supplement: Supplementary file 20 [file pone.0359084.s009.docx]

**S9 Table**

| Group | Day 7 | Day 14 | Day 21 |
| --- | --- | --- | --- |
| G3 (+ TGF-β3) | 85 ± 8 | 165 ± 12 | 210 ± 15 |
| G4 (Full composite) | 110 ± 10 | 210 ± 15 | 275 ± 18 |
