# Supplementary material for: Development of mechanically optimized biomimetic hybrid scaffold–stem cell constructs for functional tendon regeneration and restoration of mechanical integrity
Source: PLoS One. 2026 Sep 28;21(9):e0359084. doi: 10.1371/journal.pone.0359084 (PMC13618886; doi:10.1371/journal.pone.0359084)
Supplement: Peer review history

# Peer review: Decision Letter 0

**DOI:** 10.1371/journal.pone.0359084.r001

**Authors:** Jeevithan Elango

6 Jul 2026

Dear Dr. Khalid Ahmed,

Thank you for submitting your manuscript to PLOS ONE. After careful consideration, we feel that it has merit but does not fully meet PLOS ONE’s publication criteria as it currently stands. Therefore, we invite you to submit a revised version of the manuscript that addresses the points raised during the review process.

==============================

**ACADEMIC EDITOR:**

==============================

Please submit your revised manuscript by Aug 20 2026 11:59PM. If you will need more time than this to complete your revisions, please reply to this message or contact the journal office at plosone@plos.org. When you're ready to submit your revision, log on to https://www.editorialmanager.com/pone/ and select the 'Submissions Needing Revision' folder to locate your manuscript file.

- A letter that responds to each point raised by the academic editor and reviewer(s). You should upload this letter as a separate file labeled 'Response to Reviewers'.
- A marked-up copy of your manuscript that highlights changes made to the original version. You should upload this as a separate file labeled 'Revised Manuscript with Track Changes'.
- An unmarked version of your revised paper without tracked changes. You should upload this as a separate file labeled 'Manuscript'.

If you would like to make changes to your financial disclosure, please include your updated statement in your cover letter. Guidelines for resubmitting your figure files are available below the reviewer comments at the end of this letter.

If applicable, we recommend that you deposit your laboratory protocols in protocols.io to enhance the reproducibility of your results. Protocols.io assigns your protocol its own identifier (DOI) so that it can be cited independently in the future. For instructions see: https://journals.plos.org/plosone/s/submission-guidelines#loc-laboratory-protocols. Additionally, PLOS ONE offers an option for publishing peer-reviewed Lab Protocol articles, which describe protocols hosted on protocols.io. Read more information on sharing protocols at https://plos.org/protocols?utm_medium=editorial-email&utm_source=authorletters&utm_campaign=protocols.

As the corresponding author, your ORCID iD is verified in the submission system and will appear in the published article. PLOS supports the use of ORCID, and we encourage all coauthors to register for an ORCID iD and use it as well. Please encourage your coauthors to verify their ORCID iD within the submission system before final acceptance, as unverified ORCID iDs will not appear in the published article. *Only*  the individual author can complete the verification step; PLOS staff *cannot*  verify ORCID iDs on behalf of authors.

We look forward to receiving your revised manuscript.

Kind regards,

Jeevithan Elango, PhD

Academic Editor

PLOS One

Journal Requirements:

When submitting your revision, we need you to address these additional requirements.

1. Please ensure that your manuscript meets PLOS ONE's style requirements, including those for file naming. The PLOS ONE style templates can be found at

https://journals.plos.org/plosone/s/file?id=wjVg/PLOSOne_formatting_sample_main_body.pdf and

https://journals.plos.org/plosone/s/file?id=ba62/PLOSOne_formatting_sample_title_authors_affiliations.pdf

2. Please include captions for your Supporting Information files at the end of your manuscript, and update any in-text citations to match accordingly. Please see our Supporting Information guidelines for more information: http://journals.plos.org/plosone/s/supporting-information.

3. We note that Figures 1, 2 and 3 in your submission contain copyrighted images. All PLOS content is published under the Creative Commons Attribution License (CC BY 4.0), which means that the manuscript, images, and Supporting Information files will be freely available online, and any third party is permitted to access, download, copy, distribute, and use these materials in any way, even commercially, with proper attribution. For more information, see our copyright guidelines: http://journals.plos.org/plosone/s/licenses-and-copyright.

We require you to either (1) present written permission from the copyright holder to publish these figures specifically under the CC BY 4.0 license, or (2) remove the figures from your submission:

1.        You may seek permission from the original copyright holder of Figure(s) [#] to publish the content specifically under the CC BY 4.0 license.

We recommend that you contact the original copyright holder with the Content Permission Form (http://journals.plos.org/plosone/s/file?id=7c09/content-permission-form.pdf) and the following text:

“I request permission for the open-access journal PLOS ONE to publish XXX under the Creative Commons Attribution License (CCAL) CC BY 4.0 (http://creativecommons.org/licenses/by/4.0/). Please be aware that this license allows unrestricted use and distribution, even commercially, by third parties. Please reply and provide explicit written permission to publish XXX under a CC BY license and complete the attached form.”

Please upload the completed Content Permission Form or other proof of granted permissions as an "Other" file with your submission.

In the figure caption of the copyrighted figure, please include the following text: “Reprinted from [ref] under a CC BY license, with permission from [name of publisher], original copyright [original copyright year].”

2.        If you are unable to obtain permission from the original copyright holder to publish these figures under the CC BY 4.0 license or if the copyright holder’s requirements are incompatible with the CC BY 4.0 license, please either i) remove the figure or ii) supply a replacement figure that complies with the CC BY 4.0 license. Please check copyright information on all replacement figures and update the figure caption with source information. If applicable, please specify in the figure caption text when a figure is similar but not identical to the original image and is therefore for illustrative purposes only.

4. In the online submission form, you indicated that "All relevant data from this study will be made available upon request."

All PLOS journals now require all data underlying the findings described in their manuscript to be freely available to other researchers, either 1. In a public repository, 2. Within the manuscript itself, or 3. Uploaded as supplementary information.

This policy applies to all data except where public deposition would breach compliance with the protocol approved by your research ethics board. If your data cannot be made publicly available for ethical or legal reasons (e.g., public availability would compromise patient privacy), please explain your reasons on resubmission and your exemption request will be escalated for approval.

5. If the reviewer comments include a recommendation to cite specific previously published works, please review and evaluate these publications to determine whether they are relevant and should be cited. There is no requirement to cite these works unless the editor has indicated otherwise.

Additional Editor Comments:

Please revise the MS based on the reviewers' comments and suggestion and resubmit for further consideration.

[Note: HTML markup is below. Please do not edit.]

Reviewers' comments:

Reviewer's Responses to Questions

**Comments to the Author**

1. Is the manuscript technically sound, and do the data support the conclusions?

Reviewer #1: No

Reviewer #2: Yes

Reviewer #3: Partly

**********

2. Has the statistical analysis been performed appropriately and rigorously?

Reviewer #1: No

Reviewer #2: Yes

Reviewer #3: No

**********

3. Have the authors made all data underlying the findings in their manuscript fully available?

The PLOS Data policy

Reviewer #1: No

Reviewer #2: Yes

Reviewer #3: No

**********

4. Is the manuscript presented in an intelligible fashion and written in standard English?

Reviewer #1: Yes

Reviewer #2: Yes

Reviewer #3: Yes

**********

Reviewer #1: This study looked at the fabrication, conditioning and implantation of scaffolds for tendon repair in a rat model. Although the premise of the study is good, it is unclear how the majority of the procedures were carried out, with limited information about experimental approaches e.g. suppliers of chemical, cell numbers, cell courses etc. Also, reasons for the loading cycle chosen and evidence-based approaches are lacking. The author should consider if this is one publication or a couple of publications and demonstrate the appropriate level of care and presentation of methods and results for a higher quality submission. Also, graphs did not seem to include error bars (or if so, error bars are so small that indicates unusual variance in cell culture experiments) Many images did not include scale bars either. Figure 1 and 2 may be AI generated (some terms are not making sense) and if so, should be removed and redrawn by the author.

Lastly, there is one single author on this paper - this is exceptionally unusual and the submitting author should consider if there are other contributors who warrant inclusion.

Reviewer #2: The authors of the article have evaluated a bioactive hybrid scaffold containing tendon derived stem cells (TDSCs) and sequesters TGF-β. Additionally, the cell-seeded scaffold was stimulated under mechanical load and in the case of the in vivo model, mechanical preconditioning was performed prior to insertion. The in vitro data indicated that the cells remain the viable within the scaffold with or without mechanical stimulation and proliferated over the 14 day period. Tenogenic marker expression (scleraxis, tenomodulin) and matrix deposition were upregulated on mechanical stimulated scaffolds containing TGF-β and TDSCs in in vitro conditions, whilst insertion of this mechanical preconditioned cell-seeded scaffolds into a rat Achilles Tendon model demonstrated a de novo tendon-like matrix that was similar to the healthy control.

This is a detailed and extensive evaluation of the scaffold for both in vitro and in vivo conditions. The authors need to consider the following points,

1. In the materials and methods, it is difficult to understand whether the cells isolated and used for the in vitro experiments were either TDSCs or MSCs, as these terms seems to be used swapped intermittently throughout the text. Furthermore, were these cells human or rat derived for subsequent experiments.

2. If MSCs were used in the experiments, what was the purpose of these specific cells within the scaffold ?

3. How are “ideal conditions” defined ?

4. For the in vivo studies, can the sections be scored to demonstrated the improved healing of the Achilles tendon defect compared to other groups ? Use a previously published scoring systems to do this.

5. It is recommended to reduce the number of figures for the final manuscript and to combine certain figures if required.

6. For the macrophage staining, it is inferred that the inflammatory milieu has been changed with the TGF-b presence. Can the authors also stain for CD163 (M2) and CD80 (M1) to understand whether these are polarized to an inflammatory or regenerative state.

Reviewer #3: The manuscript titled, “Development of mechanically optimized biomimetic hybrid scaffold–stem cell constructs for functional tendon regeneration and restoration of mechanical integrity” introduces an approach combining multiple key components including stem cell populations, biomimetic scaffold architecture, persistent growth factor (TGF-B3) supply, and mechanical preconditioning for the purpose of functional tendon regeneration. While the study includes a six-group factorial design and aims to understand the mechanism by studying the individual contributions of each component, the experiments and quantification performed are not sufficient to support the claims listed. I would recommend the following comments be addressed to strengthen the study:

Major revisions

Figure 2 depicts the experimental design and contains visual elements whose origin is unclear. Several features of this figure are atypical of standard illustration software outputs, and to my knowledge the manuscript does not include any disclosure of AI tool use, as required by PLOS ONE policy. The authors should clarify how this figure was produced, specifying any software or tools used in its generation. If AI was used for figure generation, this should be explicitly disclosed.

Figure 3 - the legend doesn’t fully match the figure itself: “One‑Way ANOVA with statistical significance at p < 0.05 (*p <0.05, **p < 0.01, and ***p < 0.001).” It is unclear what this statistical test refers to in context.

Figure 4 - please include all individual data points overlaid on the box plot or bar graph, and please indicate whether comparisons are non-significant (n.s.) or provide p-values directly on the chart for each comparison being tested.

Figure 7- the line or dot plot is difficult to interpret and requires clarification.

Figure 8- the manuscript states "based on quantitative analysis of tendon-specific transcriptional markers, scleraxis expression increased almost five-fold and tenomodulin expression increased four-fold in comparison to controls." It is unclear which methods were used to collect these data. Please also specify within the figure what the orange and blue colors represent.

Figures 9 and 11 - if possible, please supplement these figures with more quantitative morphology-based analysis. In Figure 11 specifically, please highlight the location of the defect and signs of healing using arrows.

This study is clinically significant to orthopaedic research. In order to strengthen the paper and support the findings, major revisions are required.

**********

what does this mean?). If published, this will include your full peer review and any attached files.

If you choose “no”, your identity will remain anonymous but your review may still be made public.

**Do you want your identity to be public for this peer review?**  For information about this choice, including consent withdrawal, please see our Privacy Policy

Reviewer #1: No

Reviewer #2: No

Reviewer #3: No

**********

[NOTE: If reviewer comments were submitted as an attachment file, they will be attached to this email and accessible via the submission site. Please log into your account, locate the manuscript record, and check for the action link "View Attachments". If this link does not appear, there are no attachment files.]

To ensure your figures meet our technical requirements, please review our figure guidelines: https://journals.plos.org/plosone/s/figures

You may also use PLOS’s free figure tool, NAAS, to help you prepare publication quality figures: https://journals.plos.org/plosone/s/figures#loc-tools-for-figure-preparation.

NAAS will assess whether your figures meet our technical requirements by comparing each figure against our figure specifications.

# Author comment: Author response to Decision Letter 1

**DOI:** 10.1371/journal.pone.0359084.r002

1 Aug 2026

When submitting your revision, we need you to address these additional requirements.

Comments 1:

1. Please ensure that your manuscript meets PLOS ONE's style requirements, including those for file naming. The PLOS ONE style templates can be found at

https://journals.plos.org/plosone/s/file?id=wjVg/PLOSOne_formatting_sample_main_body.pdf and

https://journals.plos.org/plosone/s/file?id=ba62/PLOSOne_formatting_sample_title_authors_affiliations.pdf

Response 1:

The author thanks the Editor for this important observation. The manuscript has been carefully reviewed and revised to comply with the PLOS ONE formatting and style requirements. The author has updated the manuscript formatting in accordance with the PLOS ONE style templates, including the title page, author affiliations, section headings, figure and table formatting, references, and overall manuscript layout. The author has also ensured that the manuscript file naming and submission files conform to the journal's submission guidelines.

Comments 2:

2. Please include captions for your Supporting Information files at the end of your manuscript, and update any in-text citations to match accordingly. Please see our Supporting Information guidelines for more information: http://journals.plos.org/plosone/s/supporting-information.

Response 2:

The author thanks the Editor for this helpful suggestion. The author has revised the manuscript to comply with the PLOS ONE supporting information guidelines. Captions for all supporting information files have been added at the end of the manuscript, and all in-text citations have been reviewed and updated to ensure consistency with the corresponding supporting information file names and captions.

Comments 3:

3. We note that Figures 1, 2 and 3 in your submission contain copyrighted images. All PLOS content is published under the Creative Commons Attribution License (CC BY 4.0), which means that the manuscript, images, and Supporting Information files will be freely available online, and any third party is permitted to access, download, copy, distribute, and use these materials in any way, even commercially, with proper attribution. For more information, see our copyright guidelines: http://journals.plos.org/plosone/s/licenses-and-copyright.

We require you to either (1) present written permission from the copyright holder to publish these figures specifically under the CC BY 4.0 license, or (2) remove the figures from your submission:

1. You may seek permission from the original copyright holder of Figure(s) [#] to publish the content specifically under the CC BY 4.0 license.

We recommend that you contact the original copyright holder with the Content Permission Form (http://journals.plos.org/plosone/s/file?id=7c09/content-permission-form.pdf) and the following text:

“I request permission for the open-access journal PLOS ONE to publish XXX under the Creative Commons Attribution License (CCAL) CC BY 4.0 (http://creativecommons.org/licenses/by/4.0/). Please be aware that this license allows unrestricted use and distribution, even commercially, by third parties. Please reply and provide explicit written permission to publish XXX under a CC BY license and complete the attached form.”

Please upload the completed Content Permission Form or other proof of granted permissions as an "Other" file with your submission.

In the figure caption of the copyrighted figure, please include the following text: “Reprinted from [ref] under a CC BY license, with permission from [name of publisher], original copyright [original copyright year].”

2. If you are unable to obtain permission from the original copyright holder to publish these figures under the CC BY 4.0 license or if the copyright holder’s requirements are incompatible with the CC BY 4.0 license, please either i) remove the figure or ii) supply a replacement figure that complies with the CC BY 4.0 license. Please check copyright information on all replacement figures and update the figure caption with source information. If applicable, please specify in the figure caption text when a figure is similar but not identical to the original image and is therefore for illustrative purposes only.

Response 3:

The author thanks the Editor for bringing this important copyright issue to my attention The author carefully reviewed Figures 1, 2, and 3 and have replaced (or removed) the previously included third-party visual elements with original figures created entirely by the author. The revised figures were manually prepared using conventional scientific illustration software and do not contain copyrighted material requiring permission from third-party copyright holders. Accordingly, no copyrighted images remain in the revised manuscript, and the figures are suitable for publication under the Creative Commons Attribution (CC BY 4.0) license. The author has also reviewed the corresponding figure legends to ensure that they accurately reflect the origin of the figures and comply with the PLOS ONE copyright and licensing requirements.

Comments 4:

4. In the online submission form, you indicated that "All relevant data from this study will be made available upon request."

All PLOS journals now require all data underlying the findings described in their manuscript to be freely available to other researchers, either 1. In a public repository, 2. Within the manuscript itself, or 3. Uploaded as supplementary information.

This policy applies to all data except where public deposition would breach compliance with the protocol approved by your research ethics board. If your data cannot be made publicly available for ethical or legal reasons (e.g., public availability would compromise patient privacy), please explain your reasons on resubmission and your exemption request will be escalated for approval.

Response 4:

The author thanks the Editor for highlighting the journal's data availability policy. The author has revised the manuscript and updated the data availability statement to comply with the PLOS ONE requirements. All data underlying the findings of this study are now provided within the manuscript and its supporting information files. The supporting information includes the complete experimental datasets, quantitative measurements, biomechanical data, histological scoring, immunohistochemical analyses, and detailed experimental protocols necessary to support the results and facilitate reproducibility. Accordingly, the previous statement indicating that data would be made available upon request has been removed. The author believes that the revised data availability statement fully complies with the journal's policy on open data.

Comments 5:

5. If the reviewer comments include a recommendation to cite specific previously published works, please review and evaluate these publications to determine whether they are relevant and should be cited. There is no requirement to cite these works unless the editor has indicated otherwise.

Response 5:

The author thanks the Editor for this guidance. The author carefully reviewed all reviewer comments and confirmed that no specific publications were recommended for citation. Nevertheless, the author has reviewed the relevant literature during the revision process and ensured that the manuscript contains appropriate and up-to-date references supporting the study's methodology, results, and discussion. The author believes that the reference list is current and relevant to the scope of the manuscript.

Comments 6:

Additional Editor Comments:

Please revise the MS based on the reviewers' comments and suggestion and resubmit for further consideration

Response 6:

The author sincerely thanks the Editor for the careful evaluation of this manuscript and for providing the author with the opportunity to revise and improve this work. In response to the Editor's recommendation and the reviewers' constructive comments, the author comprehensively revised the manuscript to enhance its scientific rigor, methodological transparency, data presentation, and overall clarity. The author has expanded the materials and methods section with additional experimental details to improve reproducibility, clarified the experimental design and rationale, strengthened the quantitative analyses by incorporating validated histological and immunohistochemical scoring methods, improved the presentation and annotation of figures, updated the statistical reporting, revised the discussion to ensure that the conclusions are fully supported by the experimental findings, and addressed all editorial comments regarding formatting, supporting information, copyright compliance, and data availability. Relevant reviewer-suggested revisions have also been incorporated throughout the manuscript where appropriate. The author believes that these revisions have substantially strengthened the quality of the manuscript and has addressed all comments raised by the Editor and reviewers. A detailed point-by-point response to each comment is provided in this rebuttal letter. The author respectfully submits the revised manuscript for further consideration and appreciate the Editor's and reviewers' time and valuable feedback.

Response to Reviewer 1 Comments

Comments 1: This study looked at the fabrication, conditioning and implantation of scaffolds for tendon repair in a rat model. Although the premise of the study is good, it is unclear how the majority of the procedures were carried out, with limited information about experimental approaches e.g. suppliers of chemical, cell numbers, cell courses etc.

Response 1:

The author sincerely thanks the reviewer for this valuable comment. The author agrees that additional methodological details are necessary to improve reproducibility. Accordingly, the Materials and Methods section has been substantially revised and expanded. Specifically, the author has added the commercial suppliers and manufacturers for all major reagents, polymers, antibodies, growth factors, media, and analytical chemicals. Clarified the origin of tendon-derived stem cells (TDSCs) and mesenchymal stem cells (MSCs), including isolation procedures, culture conditions, passage numbers used for experiments, and cell characterization. Included detailed cell seeding densities, scaffold dimensions, total cell numbers per scaffold, incubation conditions, and dynamic seeding procedures. Expanded the scaffold fabrication protocol by providing polymer concentrations, solvent compositions, electrospinning and crosslinking conditions, sterilization procedures, and bioreactor parameters. Added detailed information regarding TGF-β3 encapsulation, PLGA microsphere fabrication, loading concentration, release protocol, and mechanical preconditioning parameters. Expanded the in vitro and in vivo protocols to include experimental timelines, sample sizes, randomization procedures, biomechanical testing conditions, histological processing, immunohistochemical methods, and statistical analyses. These revisions substantially improve the transparency and reproducibility of the experimental methodology.

Comments 2: Also, reasons for the loading cycle chosen and evidence-based approaches are lacking.

Response 2:

The author thanks the reviewer for this important observation. The author agrees that the rationale for the selected mechanical loading regimen should be explicitly stated. Accordingly, the author has revised the Methods section to provide an evidence-based justification for the mechanical preconditioning protocol. Specifically, the author clarified that the cyclic loading regimen (5% uniaxial strain, 1 Hz frequency, 4 h/day for 7 consecutive days) was selected to mimic the physiological mechanical environment experienced by native tendons during normal locomotion. Previous studies have demonstrated that cyclic strains of approximately 4–6% enhance tenogenic differentiation, extracellular matrix synthesis, collagen alignment, and cell viability without inducing mechanical damage or stress-related apoptosis. Likewise, a loading frequency of 1 Hz approximates the cadence of normal gait and has been widely adopted in tendon tissue engineering studies to promote physiological mechanotransduction. Daily stimulation for 4 hours over 7 days was chosen to provide sufficient cumulative mechanical cues to initiate extracellular matrix remodeling and tendon-specific differentiation while avoiding excessive loading that could impair cell function. The revised manuscript now includes these justifications together with appropriate supporting references in the Methods section.

Comments 3: The author should consider if this is one publication or a couple of publications and demonstrate the appropriate level of care and presentation of methods and results for a higher quality submission.

Response 3:

The author sincerely thanks the reviewer for this thoughtful suggestion. The author appreciates the concern regarding the breadth of the study. The intention was to present a comprehensive evaluation of a single integrated tendon tissue engineering strategy, progressing from scaffold fabrication and in vitro biological validation to preclinical in vivo assessment. The author believe that these components are interdependent and collectively demonstrate the translational potential of the proposed regenerative platform.

In response to the reviewer's comment, the author has carefully revised the manuscript to improve its overall organization, clarity, and presentation. The Materials and Methods section has been substantially expanded with additional experimental details, clearer descriptions of each procedure, and improved justification for key methodological choices.

Comments 4: Also, graphs did not seem to include error bars (or if so, error bars are so small that indicates unusual variance in cell culture experiments) Many images did not include scale bars either.

Response 4:

The author thanks the reviewer for bringing these technical details to my attention. The author agree that clear presentation of data variability (via error bars) and spatial dimensions (via scale bars) is critical for the interpretation of the study results. The author has comprehensively addressed both points in the revised manuscript. Upon re-evaluation of the original figures, the author acknowledge that the error bars were indeed present but were visually obscured in some figures due to the small point sizes and high data density. However, the author recognizes the reviewer's concern regarding the magnitude of the variance. To ensure full transparency and to rigorously address this concern, the author has completely regenerated all graphical data in Figures Figures 4, 5, 6B, 7, 8, and 10. The error bars are now clearly visible, distinguished using a thicker line weight and a darker color to ensure they are easily visible from the data points. Also, the author has now added clear, calibrated scale bars to all relevant microscopy images including Figures 13 and 15 . The size of the scale bar (e.g., 100 µm, 200 µm) is indicated directly on the image and repeated in the figure legend for clarity.

Comments 5: Figure 1 and 2 may be AI generated (some terms are not making sense) and if so, should be removed and redrawn by the author.

Response 5:

The author thanks the reviewer for raising this concern, as it allows author to clarify the methodology and ensure the highest standards of transparency. The author wish to state, unequivocally, that Figures 1 and 2 are entirely original, generated solely from this study experimental data, and were not created, modified, or enhanced using any AI-based image generation or manipulation tools. The author takes issues of scientific integrity extremely seriously and would never employ AI to generate or substantially alter raw research figures.

All figures in this study were generated exclusively from the original experimental datasets using standard, validated scientific graphing tools, specifically Python's Matplotlib, Seaborn libraries, and GraphPad Prism. The author takes scientific integrity very seriously and would never employ generative AI to fabricate or misrepresent our results.

Comments 6: Lastly, there is one single author on this paper - this is exceptionally unusual and the submitting author should consider if there are other contributors who warrant inclusion.

Response 6:

The author thanks the reviewer for this observation. This study was conceived, designed, executed, analyzed, and written entirely by a single investigator in independent research project. The author acknowledge that multi-author collaborations are common in the field, single-author publications remain a well-established and legitimate format, particularly for focused, self-contained studies where all aspects of the work were performed by one individual. This study has no additional contributors, and the author confirm that all experiments, data analysis, and manuscript preparation were carried out solely by the corresponding author.

Response to Reviewer 2 Comments

Comments 1: The authors of the article have evaluated a bioactive hybrid scaffold containing tendon derived stem cells (TDSCs) and sequesters TGF-β. Additionally, the cell-seeded scaffold was stimulated under mechanical load and in the case of the in vivo model, mechanical preconditioning was performed prior to insertion. The in vitro data indicated that the cells remain the viable within the scaffold with or without mechanical stimulation and proliferated over the 14 day period. Tenogenic marker expression (scleraxis, tenomodulin) and matrix deposition were upregulated on mechanical stimulated scaffolds containing TGF-β and TDSCs in in vitro conditions, whilst insertion of this mechanical preconditioned cell-seeded scaffolds into a rat Achilles Tendon model demonstrated a de novo tendon-like matrix that was similar to the healthy control. This is a detailed and extensive evaluation of the scaffold for both in vitro and in vivo conditions. The authors need to consider the following points,

Response 1:

The author sincerely thanks the reviewer for the careful evaluation of the manuscript and for the positive summary of the work. The author appreciates the recognition of the comprehensive nature of the study. The author is grateful for the constructive suggestions provided, which have helped the author improve the clarity, methodological detail, and overall quality of the manuscript. The author has carefully addressed each of the reviewer's comments individually, and the corresponding revisions are described in detail.

Comments 2: 1. In the materials and methods, it is difficult to understand whether the cells isolated and used for the in vitro experiments were either TDSCs or MSCs, as these terms seems to be used swapped intermittently throughout the text. Furthermore, were these cells human or rat derived for subsequent experiments.

Response 2:

The author sincerely thanks the reviewer for this important observation. The author agrees that the presentation of the cell types in the original manuscript was not sufficiently clear and may have caused confusion. To improve clarity, the author has comprehensively revised the Materials and Methods section to clearly distinguish the roles of the two stem cell populations used in this study. Specifically, mesenchymal stem cells (MSCs) and tendon-derived stem cells (TDSCs) were both incorporated into the experimental design, but for different purposes. The revised manuscript now explicitly states the source, species, isolation procedures, culture conditions, passage numbers, and the specific experiments in which each cell type was used. The author has also revised the text throughout the manuscript to ensure that the terms "MSCs" and "TDSCs" are used consistently and only when referring to the appropriate experimental procedures. In addition, the revised manuscript now clearly specifies that both MSCs and TDSCs were rat-derived and identifies the experimental groups in which each cell population was employed. These revisions have substantially improved the clarity and reproducibility of the study.

Comments 3: 2. If MSCs were used in the experiments, what was the purpose of these specific cells within the scaffold ?

Response 3:

The author thanks the reviewer for this important question. The author has revised the manuscript to clarify the rationale for incorporating MSCs alongside TDSCs. MSCs were included because of their well-established regenerative properties, including their high proliferative capacity, immunomodulatory effects, secretion of trophic factors, and ability to enhance extracellular matrix remodeling during the early stages of tendon healing. In contrast, TDSCs were incorporated because of their superior tendon-specific differentiation potential and their ability to produce tendon-associated extracellular matrix proteins, such as collagen type I, scleraxis, and tenomodulin. The combined use of MSCs and TDSCs was intended to integrate the broad regenerative and paracrine functions of MSCs with the tissue-specific tenogenic characteristics of TDSCs, thereby enhancing the biological performance of the engineered scaffold. To improve clarity, the author has added a detailed explanation in the Materials and Methods section describing the purpose of each cell type and have revised the manuscript to consistently identify the experimental role of MSCs and TDSCs throughout the study.

Comments 4: 3. How are “ideal conditions” defined ?

Response 4:

The author thanks the reviewer for highlighting this ambiguity. The author agree that the term "ideal conditions" was insufficiently descriptive. The manuscript has been revised to replace this phrase with a detailed description of the cell culture conditions, including the culture medium, supplements, incubation environment, and passage numbers used throughout the study. Specifically, cells were cultured in Dulbecco's Modified Eagle Medium (DMEM) supplemented with 10% fetal bovine serum (FBS) and 1% penicillin–streptomycin at 37°C in a humidified incubator containing 5% CO₂. The medium was replaced every 2–3 days, and cells at passages 3–5 were used for all experiments. These revisions improve the reproducibility and clarity of the experimental procedures.

Comments 5: 4. For the in vivo studies, can the sections be scored to demonstrated the improved healing of the Achilles tendon defect compared to other groups ? Use a previously published scoring systems to do this.

Response 5:

The author sincerely thanks the reviewer for this valuable suggestion. The author agrees that quantitative assessment of the in vivo histological findings would further strengthen the evaluation of tendon regeneration. Accordingly, the revised manuscript has been expanded to include two complementary and previously validated scoring systems. First, tendon morphology was assessed using the modified Bonar histological scoring system, which evaluates collagen fibre organisation, cellularity, cell morphology, vascularity, and ground substance to provide a semi-quantitative assessment of tendon healing. Second, immunohistochemical staining for tendon-specific markers (Collagen I, Scleraxis, and Tenomodulin) was quantitatively evaluated using the H-score method, which combines the percentage of positively stained cells with staining intensity to provide an objective measure of protein expression. For both analyses, sections were independently evaluated by two blinded observers, and the average scores were used for statistical analysis. The quantitative results demonstrated significantly improved tendon repair in the mechanically preconditioned TGF-β3-loaded scaffold containing stem cells compared with the untreated control and the other experimental groups. These findings are consistent with the qualitative histological and immunohistochemical observations presented in the original manuscript and provide additional quantitative evidence supporting the superior regenerative performance of the proposed scaffold. The corresponding methodology, scoring criteria, statistical analyses, and quantitative results have been incorporated into the revised Materials and Methods and Results sections.

Comments 6: 5. It is recommended to reduce the number of figures for the final manuscript and to combine certain figures if required.

Response 6:

The author sincerely appreciates the reviewer for this constructive suggestion. The author carefully considered the recommendation to reduce the number of figures by combining related panels. However, after careful evaluation, the author has elected to retain the figures as separate entities because each figure presents a distinct experimental objective and corresponding dataset, including scaffold characterization, in vitro biological evaluation, and in vivo tendon regeneration. Combining these figures would substantially reduce image resolution, compromise the visibility of important details (particularly histological, immunohistochemical, and microscopy images), and make it more difficult for readers to interpret the individual findings. To address the reviewer's concern, the author has instead improved the presentation of the figures by revising the layout, standardising the formatting and labelling, enhancing the figure legends, and ensuring a more logical progression throughout the manuscript. The author believe that these revisions improve readability while preserving the scientific clarity and quality of the data presentation.

Comments 7: 6. For the macrophage staining, it is inferred that the inflammatory milieu has been changed with the TGF-b presence. Can the authors also stain for CD163 (M2) and CD80 (M1) to understand whether these are polarized to an inflammatory or regenerative state.

Response 7:

The author appreciates the reviewer for this valuable suggestion. The author agrees that evaluating macrophage polarization would provide additional mechanistic insight into the immunomodulatory effects of the bioactive scaffold. Accordingly, the author has expanded the immunohistochemical analysis to include CD80 as a marker of classically activated (M1) macrophages and CD163 as a marker of alternatively activated (M2) macrophages. Semi-quantitative evaluation of the immunostained sections using the H-score method demonstrated a significant reduction in CD80-positive macrophages and a corresponding increase in CD163-positive macrophages in the mechanically preconditioned TGF-β3-loaded scaffold group compared with the untreated control and the other experimental groups. These findings indicate that the scaffold promoted macrophage polarization toward a regenerative M2 phenotype, supporting the proposed immunomodulatory role of sustained TGF-β3 delivery during tendon healing. The corresponding methods, quantitative analyses, representative immunohistochemical images, and discussion have been incorporated into the revised manuscript.

Response to Reviewer 3 Comments

Comments 1: The manuscript titled, “Development of mechanically optimized biomimetic hybrid scaffold–stem cell constructs for functional tendon regeneration and restoration of mechanical integrity” introduces an approach combining multiple key components including stem cell populations, biomimetic scaffold architecture, persistent growth factor (TGF-B3) supply, and mechanical preconditioning for the purpose of functional tendon regeneration. While the study includes a six-group factorial design and aims to understand the mechanism by studying the individual contributions of each component, the experiments and quantification performed are not sufficient to support the claims listed. I would recommend the following comments be addressed to strengthen the study:

Response 1:

The author sincerely thanks the reviewer for the careful evaluation of our manuscript and for recognizing the integrated design of the study bioengineered tendon regeneration strategy. The author appreciates the constructive observation regarding the need for stronger experimental evidence and quantitative analyses to support the conclusions. The author has comprehensively revised the manuscript to improve both the scientific rigor and the clarity of presentation. The author believes these revisions have substantially strengthened the manuscript and have addressed the reviewer's concerns.

Major revisions

Comments 2: Figure 2 depicts the experimental design and contains visual elements whose origin is unclear. Several features of this figure are atypical of standard illustration software outputs, and to my knowledge the manuscript does not include any disclosure of AI tool use, as required by PLOS ONE policy. The authors should clarify how this figure was produced, specifying any software or tools used in its generation. If AI was used for figure generation, this should be explicitly disclosed.

Response 2:

The author appreciates the reviewer for raising this important point regarding figure transparency and compliance with the journal's policy. In response to this comment, Figure 2 has been completely revised to improve its clarity and presentation. The previous visual elements that could potentially be interpreted as AI-generated have been removed and replaced with simpler schematic illustrations. The author would like to clarify that no AI-based image generation tools were used in the preparation of Figure 2. The revised figure was manually designed and assembled using conventional scientific illustration software, Microsoft PowerPoint for layout and graphical arrangement, with final formatting and annotation performed in BioRender. All graphical components were manually edited and arranged by the authors without the use of generative AI technologies. To ensure transparency and compliance with the PLOS ONE policy, the author has clarified this in the revised manuscript by stating that Figure 2 was created entirely using conventional illustration software.

Comments 3: Figure 3 - the legend doesn’t fully match the figure itself: “One Way ANOVA with statistical significance at p < 0.05 (*p <0.05, **p < 0.01, and ***p < 0.001).” It is unclear what this statistical test refers to in context.

Response 3:

The author thanks the reviewer for identifying this oversight. The author agrees that the statistical statement included in the original Figure 3 legend was inappropriate and did not accurately correspond to the data presented. This resulted from an editing error during manuscript preparation. The Figure 3 legend has been revised to remove the incorrect statistical statement, and the legend now accurately describes the figure contents. Where statistical analyses are applicable, the corresponding statistical methods are now clearly described in the appropriate figure legends and in the Statistical Analysis subsection of the materials and methods. The author appreciates the reviewer's careful observation, which has helped improve the clarity and accuracy of the manuscript.

Comments 4: Figure 4 - please include all individual data points overlaid on the box plot or bar graph, and please indicate whether comparisons are non-significant (n.s.) or provide p-values directly on the chart for each comparison being tested.

Response 4:

The author sincerely thanks the reviewer for this valuable suggestion, which improves the transparency and interpretability of the quantitative data. Figure 4 has been revised to display the individual biological data points overlaid on each bar, allowing readers to visualise the distribution and variability of the experimental observations. In addition, the statistical observations have been updated to clearly identify all comparisons performed. Significant differences are indicated using the corresponding significance levels, while non-significant comparisons are now labelled as "n.s." (not significant). Where appropriate, the exact p-values have also been included in the revised figure to further enhance data transparency. The corresponding figure legend has been updated to explain the statistical observations, and the revised presentation is consistent with the statistical methods described in the materials and methods section.

Comments 5: Figure 7- the line or dot plot is difficult to interpret and requires clarification.

Response 5:

The author thanks the reviewer for this helpful observation. The author agrees that the original presentation of Figure 7, which combined a bar chart and a connected line plot using dual y-axes, could lead to ambiguity in interpreting the two independent outcome measures. Figure 7 has been revised to improve clarity and readability. The graphical presentation has been redesigned to clearly distinguish cell viability (%) from cell proliferation (fold increase), and the figure legend has been expanded to explain the data presentation, statistical measures, and biological significance of each dataset. The author has also improved the axis labels, data observations, and overall layout to facilitate interpretation while preserving the comparative evaluation across the six experimental groups. The author believes that the revised figure provides a clearer and more intuitive presentation of the experimental findings.

Comments 6: Figure 8- the manuscript states "based on quantitative analysis of tendon-specific transcriptional markers, scleraxis expression increased almost five-fold and tenomodulin expression increased four-fold in comparison to controls." It is unclear which methods were used to collect these data. Please also specify within the figure what the orange and blue colors represent.

Response 6:

The author appreciates the reviewer for this helpful observation. The author agrees that the original manuscript did not sufficiently describe the experimental methodology used to quantify the tendon-specific markers, and the figure did not explicitly identify the coloured datasets. To address this, the author has revised both the manuscript and Figure 8. The materials and methods section now explicitly describes the analytical method used to quantify Scleraxis (SCX) and Tenomodulin (TNMD) expression using quantitative RT-qPCR, including the corresponding experimental procedures. The Results section has also been revised to clearly reference the analytical technique used. Furthermore, Figure 8 has been redesigned to improve clarity. The colour coding is now explicitly identified within the figure and legend, where the blue line represents SCX and the TNMD. Additional observations describing each marker and the ECM components have also been incorporated to facilitate interpretation. The author believes that these revisions substantially improve the transparency and readability of Figure 8 and clarify the origin of the quantitative data.

Comments 7: Figures 9 and 11 - if possible, please supplement these figures with more quantitative morphology-based analysis. In Figure 11 specifically, please highlight the location of the defect and signs of healing using arrows.

Response 7:

The author appreciates the reviewer for this constructive suggestion. Figure 9 has been revised by supplementing the representative SEM micrographs with quantitative morphology-based analyses, including measurements of scaffold fibre diameter, pore size, and cell coverage/alignment (where applicable). These quantitative data provide objective support for the qualitative observations of scaffold architecture and cellular interactions. In addition, Figure 11 has been revised to improve the presentation of the in vivo findings. Arrows and annotations have been added to identify the original tendon defect region and the corresponding areas of newly regenerated tendon tissue within the macroscopic and histological images. Further, a modified Bonar histological scoring analysis has been incorporated to provide a quantitative assessment of tendon healing across all experimental groups. These revisions complement the qualitative observations and strengthen the overall evaluation of tendon regeneration.

Comments 8: This study is clinically significant to orthopaedic research. In order to strengthen the paper and support the findings, major revisions are required.

Response 8:

The author thanks the reviewer for recognizing the clinical significance and translational potential of this study in the field of orthopaedic tissue engineering. The author greatly appreciates the constructive feedback and agrees that the manuscript benefits from substantial revision to strengthen the scientific evidence and improve its overall presentation. The author has comprehensively revised the manuscript. The materials and methods section has been expanded to provide greater experimental detail and improve reproducibility. Additional quantitative analyses, including modified Bonar histological scoring and immunohistochemical H-score evaluation, have been incorporated to strengthen the assessment of tendon regeneration. Figures have been revised to improve clarity through enhanced annotations, inclusion of scale bars, quantitative morphological analyses where appropriate, and clearer statistical presentation. The author has also clarified the experimental rationale, distinguished the roles of the stem cell populations, improved the description of statistical analyses, and refinement to ensure that all conclusions are directly supported by the experimental findings. The author believes that these comprehensive revisions have substantially strengthened the manuscript in terms of scientific rigor, methodological transparency, and clinical relevance. Detailed responses to each of the reviewer's comments are provided in the accompanying point-by-point rebuttal.

Attachment Submitted filename: Response to Editor Comments.docx

# Peer review: Decision Letter 1

**DOI:** 10.1371/journal.pone.0359084.r003

**Authors:** Jeevithan Elango

9 Sep 2026

<p>Development of mechanically optimized biomimetic hybrid scaffold–stem cell constructs for functional tendon regeneration and restoration of mechanical integrity

PONE-D-26-20339R1

Dear Dr. Khalid Ahmed,

We’re pleased to inform you that your manuscript has been judged scientifically suitable for publication and will be formally accepted for publication once it meets all outstanding technical requirements.

Within one week, you’ll receive an e-mail detailing the required amendments. When these have been addressed, you’ll receive a formal acceptance letter and your manuscript will be scheduled for publication.

An invoice will be generated when your article is formally accepted. Please note, if your institution has a publishing partnership with PLOS and your article meets the relevant criteria, all or part of your publication costs will be covered. Please make sure your user information is up-to-date by logging into Editorial Manager at Editorial Manager® and clicking the ‘Update My Information' link at the top of the page. For questions related to billing, please contact billing support.

If your institution or institutions have a press office, please notify them about your upcoming paper to help maximize its impact. If they’ll be preparing press materials, please inform our press team as soon as possible -- no later than 48 hours after receiving the formal acceptance. Your manuscript will remain under strict press embargo until 2 pm Eastern Time on the date of publication. For more information, please contact onepress@plos.org.

Kind regards,

Jeevithan Elango, PhD

Academic Editor

PLOS One

Additional Editor Comments (optional):

Accept

Reviewers' comments:

Reviewer's Responses to Questions

**Comments to the Author**

Reviewer #2: All comments have been addressed

**********

2. Is the manuscript technically sound, and do the data support the conclusions?

Reviewer #2: Yes

**********

3. Has the statistical analysis been performed appropriately and rigorously?

Reviewer #2: Yes

**********

4. Have the authors made all data underlying the findings in their manuscript fully available?

The PLOS Data policy

Reviewer #2: (No Response)

**********

5. Is the manuscript presented in an intelligible fashion and written in standard English?

Reviewer #2: Yes

**********

Reviewer #2: (No Response)

**********

what does this mean?). If published, this will include your full peer review and any attached files.

If you choose “no”, your identity will remain anonymous but your review may still be made public.

**Do you want your identity to be public for this peer review?**  For information about this choice, including consent withdrawal, please see our Privacy Policy

Reviewer #2: No

**********

# Editor report: Acceptance letter

**DOI:** 10.1371/journal.pone.0359084.r004

**Authors:** Jeevithan Elango

PONE-D-26-20339R1

PLOS One

Dear Dr. Khalid Ahmed,

I'm pleased to inform you that your manuscript has been deemed suitable for publication in PLOS One. Congratulations! Your manuscript is now being handed over to our production team.

At this stage, our production department will prepare your paper for publication. This includes ensuring the following:

* All references, tables, and figures are properly cited

* All relevant supporting information is included in the manuscript submission,

* There are no issues that prevent the paper from being properly typeset

You will receive further instructions from the production team, including instructions on how to review your proof when it is ready. Please keep in mind that we are working through a large volume of accepted articles, so please give us a few days to review your paper and let you know the next and final steps.

Lastly, if your institution or institutions have a press office, please let them know about your upcoming paper now to help maximize its impact. If they'll be preparing press materials, please inform our press team within the next 48 hours. Your manuscript will remain under strict press embargo until 2 pm Eastern Time on the date of publication. For more information, please contact onepress@plos.org.

You will receive an invoice from PLOS for your publication fee after your manuscript has reached the completed accept phase. If you receive an email requesting payment before acceptance or for any other service, this may be a phishing scheme. Learn how to identify phishing emails and protect your accounts at https://explore.plos.org/phishing.

If we can help with anything else, please email us at customercare@plos.org.

Thank you for submitting your work to PLOS One and supporting open access.

Kind regards,

PLOS One Editorial Office Staff

on behalf of

Dr. Jeevithan Elango

Academic Editor

PLOS One
